# An optimal adaptive wavelet method for first order system least squares

**DOI:** 10.1007/s00211-018-0961-7

**Published:** 2018-03-24

**Authors:** Nikolaos Rekatsinas, Rob Stevenson

**Affiliations:** Korteweg-de Vries Institute for Mathematics, University of Amsterdam, P.O. Box 94248, 1090 GE Amsterdam, The Netherlands

**Keywords:** 41A25, 42C40, 47J25, 65J15, 65N12, 65T60, 65N30, 76D05

## Abstract

In this paper, it is shown that *any* well-posed 2nd order PDE can be reformulated as a well-posed first order least squares system. This system will be solved by an adaptive wavelet solver in optimal computational complexity. The applications that are considered are second order elliptic PDEs with general inhomogeneous boundary conditions, and the stationary Navier–Stokes equations.

## Introduction

In this paper, a wavelet method is constructed for the optimal adaptive solution of *stationary* PDEs. We develop a general procedure to write *any* well-posed 2nd order PDE as a well-posed first order least squares system. The (natural) least squares formulations contain dual norms, that, however, impose no difficulties for a wavelet solver. The advantages of the first order least squares system formulation are twofold.

Firstly, regardless of the original problem, the least squares problem is symmetric and positive definite, which opens the possibility to develop an optimal adaptive solver. The obvious use of the least-squares functional as an a posteriori error estimator, however, is not known to yield a convergent method (see, however, [16] for an alternative for Poisson’s problem). As we will see, the use of the (approximate) residual in wavelet coordinates as an a posteriori error estimator does give rise to an optimal adaptive solver.

Secondly, as we will discuss in more detail in the following subsections, the optimal application of a wavelet solver to a first order system reformulation allows for a simpler, and quantitatively more efficient approximate residual evaluation than with the standard formulation of second order. Moreover, it applies equally well to semi-linear equations, as e.g. the stationary Navier–Stokes equations, and it applies to wavelets that have only *one* vanishing moment.

The approach to apply the wavelet solver to a well-posed first order least squares system reformulation also applies to *time-dependent* PDEs in simultaneous space-time variational formulations, as parabolic problems or instationary Navier–Stokes equations. With those problems, the wavelet bases consist of tensor products of temporal and spatial wavelets. Consequently, they require a different procedure for the approximate evaluation of the residual in wavelet coordinates, which will be the topic of a forthcoming work.

### Adaptive wavelet schemes, and the approximate residual evaluation

Adaptive wavelet schemes can solve well-posed linear and nonlinear operator equations at the best possible rate allowed by the basis in linear complexity [7–9, 29, 31, 32, 34]. Schemes with those properties will be called *optimal*. The schemes can be applied to PDEs, which we study in this work, as well as to integral equations [18].

There are two kinds of adaptive wavelet schemes. One approach is to apply some convergent iterative method to the infinite system in wavelet coordinates, with decreasing tolerances for the inexact evaluations of residuals [8, 9]. These schemes rely on the application of coarsening to achieve optimal rates.

The other approach is to solve a sequence of Galerkin approximations from spans of nested sets of wavelets. The (approximate) residual in wavelet coordinates of the current approximation is used as an a posteriori error estimator to make an optimal selection of the wavelets to be added to form the next set [7]. With this scheme, that is studied in the current work, the application of coarsening can be avoided [23, 34], and it turns out to be quantitatively more efficient. This approach is restricted to PDOs whose Fréchet derivatives are symmetric and positive definite (compact perturbations can be added though, see [22]).

A key computational ingredient of both schemes is the approximate evaluation of the residual in wavelet coordinates. Let us discuss this for a linear operator equation $A u =f$, with, for some separable Hilbert spaces $\mathscr {H}$ and $\mathscr {K}$, for convenience over $\mathbb {R}$, $f \in \mathscr {K}'$ and $A \in \mathcal {L}\mathrm {is}(\mathscr {H},\mathscr {K}')$ (i.e., $A \in \mathcal {L}(\mathscr {H},\mathscr {K}')$ and $A^{-1} \in \mathcal {L}(\mathscr {K}',\mathscr {H})$).

Equipping $\mathscr {H}$ and $\mathscr {K}$ with *Riesz bases* $\Psi ^\mathscr {H}$, $\Psi ^\mathscr {K}$, formally viewed as column vectors, $A u =f$ can be equivalently written as a bi-infinite system of coupled scalar equations $\mathbf{A} \mathbf{u}=\mathbf{f}$, where $\mathbf{f}=f(\Psi ^\mathscr {K})$ is the infinite ‘load vector’, $\mathbf{A}=(A \Psi ^\mathscr {H})(\Psi ^\mathscr {K})$ is the infinite ‘stiffness’ or system matrix, and $u=\mathbf{u}^\top \Psi ^\mathscr {H}$.

Here we made use of following notations:

#### Notation 1.1

For countable collections of functions $\Sigma$ and $\Upsilon$, we write $g(\Sigma )=[g(\sigma )]_{\sigma \in \Sigma }$, $M(\Sigma )(\Upsilon )=[M(\sigma )(\upsilon )]_{\upsilon \in \Upsilon , \sigma \in \Sigma }$, and $\langle \Upsilon , \Sigma \rangle = [\langle \upsilon , \sigma \rangle ]_{\upsilon \in \Upsilon , \sigma \in \Sigma }$, assuming *g*, *M*, and $\langle \,,\,\rangle$ are such that the expressions at the right-hand sides are well-defined.

The space of square summable vectors of reals indexed over a countable index set $\vee$ will be denoted as $\ell _2(\vee )$ or simply as $\ell _2$. The norm on this space will be simply denoted as $\Vert \cdot \Vert$.

As a consequence of $A \in \mathcal {L}\mathrm {is}(\mathscr {H},\mathscr {K}')$, we have that $\mathbf{A} \in \mathcal {L}\mathrm {is}(\ell _2,\ell _2)$. For the moment, let us additionally assume that $\mathbf{A}$ is symmetric and positive definite, as when $\mathscr {K}=\mathscr {H}$, $(Au)(v)= (Av)(u)$ and $(Au)(u) \gtrsim \Vert u\Vert _\mathscr {H}^2$ ($u,v \in \mathscr {H}$). If this is not the case, then the following can be applied to the normal equations $\mathbf{A}^\top \mathbf{A} \mathbf{u}=\mathbf{A}^\top \mathbf{f}$.

For the finitely supported approximations ${\tilde{\mathbf{u}}}$ to $\mathbf{u}$ that are generated inside the adaptive wavelet scheme, the residual $\mathbf{r}=\mathbf{f} -\mathbf{A} {\tilde{\mathbf{u}}}$ has to be approximated within a sufficiently small relative tolerance. The resulting scheme has been shown to converge with the best possible rate: Whenever $\mathbf{u}$ *can* be approximated at rate *s*, i.e. $\mathbf{u} \in \mathcal {A}^s$, meaning that for any $N \in \mathbb {N}$ there *exists* a vector of length *N* that approximates $\mathbf{u}$ within tolerance ${\mathcal {O}}(N^{-s})$, the approximations produced by the scheme converge with this rate *s*. Moreover, the scheme has *linear computational complexity* under the *cost condition* that

1.1 $$\begin{aligned}&the \,approximate \, residual \,evaluation \, within \,an \,(absolute) \,tolerance \, \varepsilon \gtrsim \Vert \mathbf{r}\Vert \,\nonumber \\&\quad requires \,not \,more \,than \, {\mathcal {O}}(\varepsilon ^{-1/s}+\#\,\mathrm{supp}\,{\tilde{\mathbf{u}}})\, operations. \end{aligned}$$

The lower bound on $\varepsilon$ reflects the fact that inside the adaptive scheme, it is never needed to approximate a residual more accurately than within a sufficiently small, but fixed relative tolerance. The validity of (1.1) will require additional properties of $\Psi ^\mathscr {H}$ and $\Psi ^\mathscr {K}$ in addition to being Riesz bases. For that reason we consider wavelet bases.

The *standard way* to approximate the residual within tolerance $\varepsilon$ is to approximate both $\mathbf{f}$ and $\mathbf{A} {\tilde{\mathbf{u}}}$ *separately* within tolerance $\varepsilon /2$. Under reasonable assumptions, $\mathbf{f}$ can be approximated within tolerance $\varepsilon /2$ by a vector of length ${\mathcal {O}}(\varepsilon ^{-1/s})$.

For the approximation of $\mathbf{A} {\tilde{\mathbf{u}}}$, it is used that, thanks to the properties of the wavelets as having vanishing moments, each column of $\mathbf{A}$, although generally infinitely supported, can be well approximated by finitely supported vectors. In the approximate matrix-vector multiplication routine introduced in [7], known as the **APPLY**-routine, the accuracy with which a column is approximated is judiciously chosen *depending* on the size of the corresponding entry in the input vector ${\tilde{\mathbf{u}}}$. It has been shown to realise a tolerance $\varepsilon /2$ at cost ${\mathcal {O}}(\varepsilon ^{-1/s}|{\tilde{\mathbf{u}}}|_{\mathcal {A}^{s}}^{1/s}+\#\,\mathrm{supp}\,{\tilde{\mathbf{u}}})$, for any *s* in some range $(0,s^*]$. For wavelets that have *sufficiently many* vanishing moments, this range was shown to include the range of $s \in (0,s_{\max }]$ for which, in view of the order of the wavelets, $\mathbf{u} \in \mathcal {A}^s$ can possibly be expected (cf. [28]). Using that for the approximations ${\tilde{\mathbf{u}}}$ to $\mathbf{u}$ that are generated inside the adaptive wavelet scheme, it holds that $|{\tilde{\mathbf{u}}}|_{\mathcal {A}^{s}} \lesssim |\mathbf{u}|_{\mathcal {A}^{s}}$, in those cases the cost condition is satisfied, and so the adaptive wavelet scheme is optimal.

The **APPLY**-routine, however, is quite difficult to implement. Note, in particular, that its outcome depends *nonlinearly* on the input vector ${\tilde{\mathbf{u}}}$. Furthermore, in experiments, the routine turns out to be quantitatively expensive. Finally, although it has been generalized to certain classes of semi-linear PDEs, in those cases it has not been shown that $s^* \ge s_{\max }$, meaning that for nonlinear problems the issue of optimality is actually open.

### An alternative for the **APPLY** routine

A main goal of this paper is to develop a quantitatively efficient alternative for the **APPLY**-routine, that, moreover, gives rise to provable optimal adaptive wavelet schemes for classes of *semi-linear* PDEs, and applies to wavelets with only *one vanishing moment*. As an introduction, we consider the model problem of Poisson’s equation in one space dimension $$ \left\{ \begin{array}{ll} -\,u''=f &{}\text { on } (0,1),\\ u=0 &{}\text { at }\{0,1\}, \end{array} \right. $$ that, in standard variational form, reads as finding $u \in \mathscr {H}:=H^1_0(0,1)$ such that

$$\begin{aligned} \langle u', v' \rangle _{L_2(0,1)}= \langle f, v \rangle _{L_2(0,1)} ,\quad (v \in \mathscr {K}:=H^1_0(0,1)), \end{aligned}$$

where, by identifying $L_2(0,1)'$ with $L_2(0,1)$ and using that $H_0^1(0,1) \hookrightarrow L_2(0,1)$ is dense, $\langle \,,\, \rangle _{L_2(0,1)}$ is also used to denote the duality pairing on $H^{-1}(0,1)\times H^1_0(0,1)$. We consider piecewise polynomial, locally supported wavelet Riesz bases $\Psi ^\mathscr {H}$ and $\Psi ^\mathscr {K}$ for $H^1_0(0,1)$. Let us exclusively consider *admissible* approximations ${\tilde{\mathbf{u}}}$ to $\mathbf{u}$ in the sense that their finite supports form *trees*, meaning that if $\lambda \in \mathrm{supp}\,{\tilde{\mathbf{u}}}$, then then there exists a $\mu \in \mathrm{supp}\,{\tilde{\mathbf{u}}}$, whose level is one less than the level of $\lambda$, and ${{\mathrm{meas}}}(\mathrm{supp}\,\psi ^\mathscr {H}_\lambda \cap \mathrm{supp}\,\psi ^\mathscr {H}_\mu )>0$. It is known that the approximation classes $\mathcal {A}^s$ become only ‘slightly’ smaller by this restriction to tree approximation compared to unconstrained approximation (cf. [11]). What is more, the restriction to tree approximation seems mandatory anyway to construct an optimal algorithm for nonlinear operators. The benefit of tree approximation is that $\tilde{u}:={\tilde{\mathbf{u}}}^\top \Psi ^\mathscr {H}$ has an alternative, ‘single-scale’ representation as a piecewise polynomial w.r.t. a partition ${\mathcal {T}}_1$ of (0, 1) with $\# {\mathcal {T}}_1 \lesssim \#{\mathrm{supp}\,{\tilde{\mathbf{u}}}}$.

For the moment, let us make the *additional assumption* that $\Psi ^\mathscr {H}$ is selected inside $H^2(0,1)$. Then, for an admissible ${\tilde{\mathbf{u}}}$, with its support denoted as $\Lambda ^\mathscr {H}$, integration-by-parts shows that

$$\begin{aligned} \mathbf{r}:=\mathbf{f}-\mathbf{A} {\tilde{\mathbf{u}}}=\langle \Psi ^\mathscr {K},f+\tilde{u}'' \rangle _{L_2(0,1)}, \end{aligned}$$

where $\tilde{u}''$ is piecewise polynomial w.r.t. ${\mathcal {T}}_1$. If $\mathbf{u} \in \mathcal {A}^s$, then for any $\varepsilon >0$ there exists a piecewise polynomial $f_\varepsilon$ w.r.t. a partition $\mathcal {T}_2$ of (0,1) into ${\mathcal {O}}(\varepsilon ^{-1/s})$ subintervals such that $\Vert f-f_\varepsilon \Vert _{H^{-1}(0,1)} \le \varepsilon$.^1^ The term $f-f_\varepsilon$ is commonly referred to as *data oscillation*.

The function $f_\varepsilon +\tilde{u}''$ is piecewise polynomial w.r.t. the smallest common refinement $\mathcal {T}$ of $\mathcal {T}_1$ and $\mathcal {T}_2$. Thanks to this piecewise smoothness of $f_\varepsilon +\tilde{u}''$ w.r.t. $\mathcal {T}$, and the property of $\psi _\lambda ^\mathscr {K}$ having *one* vanishing moment, $|\langle \psi _\lambda ^\mathscr {K}, f_\varepsilon +\tilde{u}''\rangle _{L_2(0,1)}|$ is decreasing as function of the minimal difference of the *level* of $\psi _\lambda ^\mathscr {K}$ and that of any subinterval in $\mathcal {T}$ that has non-empty intersection with $\mathrm{supp}\,\psi _\lambda ^\mathscr {K}$. Here with the level of $\psi _\lambda ^\mathscr {K}$ or that of an interval $\omega$, we mean an $\ell \in \mathbb {N}_0$ such that $2^{-\ell } \eqsim {{\mathrm{diam}}}(\mathrm{supp}\,\psi _\lambda ^\mathscr {K})$ or $2^{-\ell } \eqsim {{\mathrm{diam}}}\,\omega$, respectively. In particular, given a constant $\varsigma >0$, there exists a constant *k*, such that by dropping all $\lambda$ for which the aforementioned minimal level difference exceeds *k*, the remaining indices form a tree $\Lambda ^\mathscr {K}$ with $\# \Lambda ^\mathscr {K}\lesssim \# \mathcal {T}\lesssim \varepsilon ^{-1/s}+\# \Lambda ^\mathscr {H}$ (dependent on *k*), and (see Proposition A.1)

$$\begin{aligned} \Vert \mathbf{f}_\varepsilon -\mathbf{A} {\tilde{\mathbf{u}}}-(\mathbf{f}_\varepsilon -\mathbf{A} {\tilde{\mathbf{u}}})|_{\Lambda ^\mathscr {K}}\Vert \le \varsigma \Vert f_\varepsilon +\tilde{u}''\Vert _{H^{-1}(0,1)}&\le \varsigma \Vert f+\tilde{u}''\Vert _{H^{-1}(0,1)}+\varsigma \varepsilon \\&\lesssim \varsigma \Vert u-\tilde{u}\Vert _{H^1(0,1)}+\varsigma \varepsilon , \end{aligned}$$

and so, using $\Vert \mathbf{r}\Vert \eqsim \Vert u-\tilde{u}\Vert _{H^1(0,1)}$ and $\Vert \mathbf{f}-\mathbf{f}_\varepsilon \Vert \eqsim \Vert f-f_\varepsilon \Vert _{H^{-1}(0,1)}$,

$$\begin{aligned} \Vert \mathbf{r}-\mathbf{r}|_{\Lambda ^\mathscr {K}}\Vert \lesssim \varsigma \Vert \mathbf{r}\Vert +\varepsilon . \end{aligned}$$

Note that for $\varsigma$ being sufficiently small, and so *k* sufficiently large, by taking $\varepsilon$ suitably the approximate residual will meet any accuracy that is required in the cost Condition (1.1).

By selecting ‘single scale’ collections $\Phi ^\mathscr {H}$ and $\Phi ^\mathscr {K}$ with ${{\mathrm{span}}}\, \Phi ^\mathscr {H}\supseteq {{\mathrm{span}}}\,\Psi ^\mathscr {H}|_{\Lambda ^\mathscr {H}}$ and ${{\mathrm{span}}}\,\Phi ^\mathscr {K}\supseteq {{\mathrm{span}}}\,\Psi ^\mathscr {K}|_{\Lambda ^\mathscr {K}}$, and $\# \Phi ^\mathscr {H}\lesssim \# \Lambda ^\mathscr {H}$ and $\# \Phi ^\mathscr {K}\lesssim \# \Lambda ^\mathscr {K}$, this approximate residual $\mathbf{r}|_{\Lambda ^\mathscr {K}}$ can be computed in ${\mathcal {O}}(\Lambda ^\mathscr {K})$ operations as follows: First express $\tilde{u}$ in terms of $\Phi ^\mathscr {H}$ by applying a multi-to-single scale transformation to ${\tilde{\mathbf{u}}}$, then apply to this representation the sparse stiffness matrix $\langle (\Phi ^\mathscr {K})',(\Phi ^\mathscr {H})' \rangle _{L_2(0,1)}$, subtract $\langle \Phi ^\mathscr {K},f \rangle _{L_2(0,1)}$, and finally apply the transpose of the multi-to-single scale transformation involving $\Psi ^\mathscr {K}|_{\Lambda ^\mathscr {K}}$ and $\Phi ^\mathscr {K}$. This approximate residual evaluation thus satisfies the cost condition for optimality, it is relatively easy to implement, and it is observed to be quantitatively much more efficient.

It furthermore generalizes to semi-linear operators, in any case for nonlinear terms that are multivariate polynomials in *u* and derivatives of *u*. Indeed, as an example, suppose that instead of $-u''=f$ the equation reads as $-u'' +u^3=f$. Then the residual is given by $\langle \Psi ^\mathscr {K},f+\tilde{u}''-\tilde{u}^3 \rangle _{L_2(0,1)}$. Since $f_\varepsilon +\tilde{u}''-\tilde{u}^3$ is a piecewise polynomial w.r.t. $\mathcal {T}$, the same arguments shows that $\langle \Psi ^\mathscr {K},f+\tilde{u}''-\tilde{u}^3 \rangle _{L_2(0,1)}\big |_{\Lambda ^\mathscr {K}}$ is a valid approximate residual.

The essential idea behind our approximate residual evaluation is that, after the replacement of *f* by $f_\varepsilon$, the different terms that constitute the residual are expressed in a *common* dictionary, before the residual, *as a whole*, is integrated against $\Psi ^\mathscr {K}$. In our simple one-dimensional example this was possible by selecting $\Psi ^\mathscr {H}\subset H^2(0,1)$, so that the operator could be applied to the wavelets in strong, or more precisely, mild sense, meaning that the result of the application lands in $L_2(0,1)$. It required piecewise smooth, globally $C^1$-wavelets. Although the same approach applies in more dimensions, there, except on product domains, the construction of $C^1$-wavelet bases is cumbersome. For that reason, our approach will be to write a PDE of second order as a *system of PDEs of first order*. It will turn out that there are several possibilities to do so.

### A common first order system least squares formulation

To introduce ideas, let us again consider the model problem of Poisson’s equation in one dimension. By introducing the additional unknown $\theta =u'$, for given $f \in L_2(0,1)$ this PDE can be written as the first order system of finding $(u,\theta ) \in H^1_0(0,1) \times H^1(0,1)$ such that

$$\begin{aligned} \vec {H}(u,\theta ):=(-\theta '-f,-u'+\theta )=\vec {0} \quad \text {in } L_2(0,1)\times L_2(0,1). \end{aligned}$$

The corresponding homogeneous operator^2^ $\vec {H}^\mathrm{h}:=(v,\eta )\mapsto (-\eta ',-v'+\eta )$ is in $\mathcal {L}\mathrm {is}(H^1_0(0,1) \times H^1(0,1), L_2(0,1) \times L_2(0,1))$ (cf. [30, (proof of) Thm. 3.1]). To arrive at a symmetric and positive definite system, we consider the least squares problem of solving

$$\begin{aligned} \mathop {{{\mathrm{argmin}}}}\limits _{(u,\theta ) \in H^1_0(0,1) \times H^1(0,1)} \Vert \vec {H}(u,\theta )\Vert _{L_2(0,1)\times L_2(0,1)}^2. \end{aligned}$$

Its solution solves the Euler–Lagrange equations

$$\begin{aligned} \langle \vec {H}^h(v,\eta ),\vec {H}(u,\theta )\rangle _{L_2(0,1) \times L_2(0,1)}=0 \quad ((v,\eta ) \in H^1_0(0,1) \times H^1(0,1)). \end{aligned}$$

which in this setting are known as the *normal equations*.

To these equations we apply the adaptive wavelet scheme, so with ‘$\mathscr {H}$’$=$‘$\mathscr {K}$’$=H^1_0(0,1) \times H^1(0,1)$, (‘*A*’$(u,\theta ))(v,\eta ):=\langle \vec {H}^h(v,\eta ),\vec {H}^h(u,\theta )\rangle _{L_2(0,1) \times L_2(0,1)}$ and right-hand side ‘*f*’$(v,\eta ):=\langle f, -\eta ' \rangle _{L_2(0,1)}$. From $\vec {H}^\mathrm{h}$ being a homeomorphism with its range, i.e.,

$$\begin{aligned} \Vert \vec {H}^\mathrm{h}(v,\eta )\Vert _{L_2(0,1) \times L_2(0,1)} \eqsim \Vert (v,\eta )\Vert _{H^1_0(0,1) \times H^1(0,1)}, \end{aligned}$$

being a consequence of $\vec {H}^\mathrm{h}$ being even boundedly invertible between the full spaces, it follows that the bilinear form is bounded, symmetric, and coercive. After equipping $H_0^1(0,1)$ and $H^1(0,1)$ with wavelet Riesz bases $\Psi ^{H^1_0}$ and $\Psi ^{H^1}$, for admissible ${\tilde{\mathbf{u}}}$ and $\varvec{\tilde{\theta }}$, with $\tilde{u}:={\tilde{\mathbf{u}}}^\top \Psi ^{H^1_0}$ and $\tilde{\theta }:=\varvec{\tilde{\theta }}^\top \Psi ^{H^1}$ the residual reads as

1.2 $$\begin{aligned} \mathbf{r}=\left[ \begin{array}{l} \mathbf{r}_1 \\ \mathbf{r}_2 \end{array} \right] = \left[ \begin{array}{l} \langle (\Psi ^{H^1_0})',\tilde{u}'-\tilde{\theta }\rangle _{L_2(0,1)} \\ \langle (\Psi ^{H^1})',\tilde{\theta }'+f\rangle _{L_2(0,1)} +\langle \Psi ^{H^1},\tilde{\theta }-\tilde{u}'\rangle _{L_2(0,1)} \end{array} \right] . \end{aligned}$$

The construction of an approximate residual follows the same lines as described before for the standard variational formulation.^3^ The functions $\tilde{u}',\,\tilde{\theta },\,\tilde{\theta }'$ are piecewise polynomials w.r.t. a partition $\mathcal {T}_1$ of (0, 1) into ${\mathcal {O}}(\#\,\mathrm{supp}\,{\tilde{\mathbf{u}}}+\#\,\mathrm{supp}\,\varvec{\tilde{\theta }})$ subintervals. If $(\mathbf{u},\varvec{\theta }) \in \mathcal {A}^s$, then there exists a piecewise polynomial $f_\varepsilon$ w.r.t. a partition $\mathcal {T}_2$ of (0, 1) into ${\mathcal {O}}(\varepsilon ^{-1/s})$ subintervals such that $\Vert f-f_\varepsilon \Vert _{L_2(0,1)} \le \varepsilon$. Thanks to the piecewise smoothness of $\tilde{u}'-\tilde{\theta }$ and $\tilde{\theta }'+f_\varepsilon$, there exist trees $\Lambda ^{H^1_0}$ and $\Lambda ^{H^1}$, with $\# \Lambda ^{H^1_0}+ \# \Lambda ^{H^1} \lesssim \#\mathcal {T}_1+\#\mathcal {T}_2$ (dependent on $\varsigma$), such that

$$\begin{aligned} \left\| \mathbf{r}- \left[ \begin{array}{l} \mathbf{r}_1|_{\Lambda ^{H^1_0}} \\ \mathbf{r}_2|_{\Lambda ^{H^1}} \end{array}\right] \right\| \lesssim \varsigma (\Vert \tilde{u}'-\tilde{\theta }\Vert _{L_2(0,1)}+\Vert \tilde{\theta }'+f_\varepsilon \Vert _{L_2(0,1)}) +\varepsilon \lesssim \varsigma \Vert \mathbf{r}\Vert +\varepsilon . \end{aligned}$$

Since the approximate residual can be evaluated in ${\mathcal {O}}(\# \Lambda ^{H^1_0} \cup \Lambda ^{H^1})$ operations, we conclude that it satisfies the cost Condition (1.1) for optimality of the adaptive wavelet scheme.

#### Remark 1.2

Recall that, as always with least squares formulations, the same results are valid when lower order, possibly *non-symmetric* terms are added to the second order PDE, as long as the standard variational formulation remains well-posed. Furthermore, as we will discuss, least squares formulations allows to handle *inhomogeneous boundary conditions*. Finally, as we will see, the approach of reformulating a 2nd order PDE as a first order least squares problem, and then optimally solving the normal equations applies to *any* well-posed PDE, not necessarily being elliptic.

In [17] we applied the adaptive wavelet scheme to a least squares formulation of the current, common type. *Disadvantages* of this formulation are that (i) it requires that $f \in L_2(0,1)$, instead of $f \in H^{-1}(0,1)$ as allowed in the standard variational formulation. Related to that, and more importantly, for a semi-linear equation $-u''+N(u)=f$, (ii) it is needed that *N* maps $H^1_0(0,1)$ into $L_2(0,1)$, instead of into $H^{-1}(0,1)$. Finally, with the generalization of this least squares formulation to more than one space dimensions, (iii) the space $H^1(0,1)$ for $\theta$ reads as $H({{\mathrm{div}}};\Omega )$. In [17], for two-dimensional connected polygonal domains $\Omega$, we managed to construct a wavelet Riesz basis for $H({{\mathrm{div}}};\Omega )$. This construction, however, relied on the fact that, in two dimensions, any divergence-free function is the curl of an $H^1$-function. To the best of our knowledge, wavelet Riesz bases for $H({{\mathrm{div}}};\Omega )$ for non-product domains in three and more dimensions have not been constructed.

In the next subsection, we describe a prototype of a least-squares formulation with which these disadvantages (i)–(iii) are avoided.

### A seemingly unpractical least squares formulation

The first order system least squares formulation that will be studied in this paper reads, for the model problem, as follows: again we introduce $\theta =u'$, but now consider the first order system of finding $(u,\theta ) \in H^1_0(0,1) \times L_2(0,1)$ such that

$$\begin{aligned} \vec {H}(u,\theta ):=(D'\theta +f,-u'+\theta )=\vec {0} \quad \text {in } H^{-1}(0,1)\times L_2(0,1). \end{aligned}$$

where $(D'\theta )(v):=-\langle \theta ,v'\rangle _{L_2(0,1)}$, i.e., $D'\theta$ is the distributional derivative of $\theta$.

In ‘primal’ mixed form, this system reads as

$$\begin{aligned} \langle \theta ,v'\rangle _{L_2(0,1)}+\langle \theta -u',\eta \rangle _{L_2(0,1)}=\langle f,v \rangle _{L_2(0,1)} \quad ((v,\eta ) \in H^1_0(0,1)\times L_2(0,1)). \end{aligned}$$

The corresponding homogeneous operator $\vec {H}^\mathrm{h}$ is in $\mathcal {L}\mathrm {is}(H^1_0(0,1) \times L_2(0,1), H^{-1}(0,1) \times L_2(0,1))$, and the least squares problem reads as solving

1.3 $$\begin{aligned} \mathop {{{\mathrm{argmin}}}}\limits _{(u,\theta ) \in H^1_0(0,1) \times L_2(0,1)} \Vert \vec {H}(u,\theta )\Vert _{H^{-1}(0,1)\times L_2(0,1)}^2, \end{aligned}$$

with normal equations reading as

1.4 $$\begin{aligned} \begin{aligned} 0&=\langle \vec {H}^h(v,\eta ),\vec {H}(u,\theta )\rangle _{H^{-1}(0,1) \times L_2(0,1)}\\&= \langle D'\eta ,D'\theta -f \rangle _{H^{-1}(0,1)}+\langle -v'+\theta ,-u'+\theta \rangle _{L_2(0,1)} \end{aligned} \end{aligned}$$

($$(v,\eta ) \in H^1_0(0,1) \times L_2(0,1)$$).

In the terminology from [3], our current least squares problem is identified as being unpractical because of the appearance of the dual norm. To deal with this, as in [19], we select *some* wavelet Riesz basis $\Psi ^{\hat{H}^1_0}$ for $H^1_0(0,1)$, and replace the norm on $H^{-1}(0,1)$ in (1.3) by the *equivalent* norm defined by $\Vert g(\Psi ^{\hat{H}^1_0})\Vert$ for $g \in H^{-1}(\Omega )$. Correspondingly, in (1.4) we replace the inner product $\langle g,h\rangle _{H^{-1}(0,1)}$ by $g(\Psi ^{\hat{H}^1_0})^\top h(\Psi ^{\hat{H}^1_0})$, so that the resulting normal equations read as finding $(u,\theta ) \in H^1_0(0,1) \times L_2(0,1)$ such that

$$\begin{aligned}&\langle \eta , (\Psi ^{\hat{H}^1_0})'\rangle _{L_2(0,1)}\big \{\langle (\Psi ^{\hat{H}^1_0})',\theta \rangle _{L_2(0,1)}-\langle \Psi ^{\hat{H}^1_0},f\rangle _{L_2(0,1)}\big \}\\&\quad +\,\langle -v'+\eta ,-u'+\theta \rangle _{L_2(0,1)}=0 \end{aligned}$$

for all $$(v,\eta ) \in H^1_0(0,1) \times L_2(0,1)$$.

To apply the adaptive wavelet scheme to these normal equations, we equip $H^1_0(0,1)$ and $L_2(0,1)$ with wavelet Riesz bases $\Psi ^{H^1_0}$ and $\Psi ^{L_2}$, respectively. When these bases have order $p+1$ and *p*, the best possible convergence rate $s_{\max }$ will be equal to *p* (*p* / *n* on an *n*-dimensional domain). Note that the order of the basis $\Psi ^{\hat{H}^1_0}$ is irrelevant.

For approximations $(\tilde{u},\tilde{\theta })=({\tilde{\mathbf{u}}}^\top \Psi ^{H^1},\varvec{\tilde{\theta }}^\top \Psi ^{L_2})$ for admissible ${\tilde{\mathbf{u}}}$ and $\varvec{\tilde{\theta }}$, the residual $\mathbf{r}$ of $({\tilde{\mathbf{u}}},\varvec{\tilde{\eta }})$ reads as

$$\begin{aligned} \left[ \begin{array}{l} \langle (\Psi ^{H^1_0})',\tilde{u}'-\tilde{\theta } \rangle _{L_2(0,1)}\\ \langle \Psi ^{L_2}, (\Psi ^{\hat{H}^1_0})'\rangle _{L_2(0,1)} \big \{\langle (\Psi ^{\hat{H}^1_0})',\tilde{\theta } \rangle _{L_2(0,1)}- \langle \Psi ^{\hat{H}^1_0},f\rangle _{L_2(0,1)} \big \}+\langle \Psi ^{L_2},\tilde{\theta }-\tilde{u}'\rangle _{L_2(0,1)} \end{array}\right] \end{aligned}$$

that, under the *additional condition* that $\Psi ^{L_2} \subset H^1(0,1)$, and thus $\tilde{\theta } \in H^1(0,1)$, is equal to

1.5 $$\begin{aligned} \left[ \begin{array}{l} \mathbf{r}_1 \\ \mathbf{r}_2 \end{array}\right] =\left[ \begin{array}{@{}l@{}} \langle (\Psi ^{H^1_0})',\tilde{u}'-\tilde{\theta }\rangle _{L_2(0,1)}\\ -\langle \Psi ^{L_2}, (\Psi ^{\hat{H}^1_0})'\rangle _{L_2(0,1)} \langle \Psi ^{\hat{H}^1_0},\tilde{\theta }'+f\rangle _{L_2(0,1)}+\langle \Psi ^{L_2},\tilde{\theta }-\tilde{u}'\rangle _{L_2(0,1)} \end{array}\right] . \end{aligned}$$

This last step is essential because it allows us, after the replacement of *f* by a piecewise polynomial $f_\varepsilon$, to express $\tilde{\theta }'+f_\varepsilon$ in a common dictionary. The additional condition is satisfied by piecewise polynomial, globally *continuous* wavelets, which are available on general domains in multiple dimensions.

In view of the previous discussions, to describe the approximate residual evaluation, it suffices to consider the term $\langle \Psi ^{L_2}, \mathbf{z}^\top (\Psi ^{\hat{H}^1_0})'\rangle _{L_2(0,1)}$ with $\mathbf{z}:= \langle \Psi ^{\hat{H}^1_0},f+\tilde{\theta }'\rangle _{L_2(0,1)}$. The by now familiar approach is applied twice: the function $\tilde{\theta }'$ is piecewise polynomial w.r.t. a partition $\mathcal {T}_1$ into ${\mathcal {O}}(\#\,\mathrm{supp}\,\varvec{\tilde{\theta }})$ subintervals. If $(\mathbf{u},\varvec{\theta }) \in \mathcal {A}^s$, then there exists a piecewise polynomial $f_\varepsilon$ w.r.t. a partition $\mathcal {T}_2$ of (0,1) into ${\mathcal {O}}(\varepsilon ^{-1/s})$ subintervals such that $\Vert f-f_\varepsilon \Vert _{H^{-1}(0,1)} \le \varepsilon$. Consequently, there exists a tree $\Lambda ^{\hat{H}^1_0}$ with $\# \Lambda ^{\hat{H}^1_0} \lesssim \#\,\mathrm{supp}\,\varvec{\tilde{\theta }}+\varepsilon ^{-1/s}$ (dependent on $\varsigma$) such that $\Vert \mathbf{z}-\mathbf{z}|_{\Lambda ^{\hat{H}^1_0}}\Vert \lesssim \varsigma \Vert f+\tilde{\theta }'\Vert _{H^{-1}(0,1)}+\varepsilon$. The function $\tilde{z}:=\mathbf{z}|_{\Lambda ^{\hat{H}^1_0}}^\top \Psi ^{\hat{H}^1_0}$ is piecewise polynomial w.r.t. a partition $\mathcal {T}_2$ of (0,1) into ${\mathcal {O}}(\# \Lambda ^{\hat{H}^1_0})$ subintervals. Consequently, exists a tree $\Lambda _1^{L_2}$ with $\# \Lambda _1^{L_2} \lesssim \# \Lambda ^{\hat{H}^1_0}$ (dependent on $\varsigma$) such that $\Vert \langle \Psi ^{L_2}, \tilde{z}'\rangle _{L_2(0,1)}-\langle \Psi ^{L_2}, \tilde{z}'\rangle _{L_2(0,1)}\big |_{\Lambda _1^{L_2}}\Vert \le \varsigma \Vert \tilde{z}\Vert _{H^1(0,1)} \lesssim \varsigma (\Vert \mathbf{z}\Vert +\Vert \mathbf{z}-\mathbf{z}|_{\Lambda ^{\hat{H}^1_0}}\Vert ) \lesssim \varsigma (\Vert f+\tilde{\theta }'\Vert _{H^{-1}(0,1)}+\varepsilon )$. Combining this with the approximations for the other two terms that constitute the residual, we infer that there exist trees $\Lambda ^{H^1_0}$ and $\Lambda ^{L_2}$ with $\# \Lambda ^{H^1_0}+\# \Lambda ^{L_2} \lesssim \#\,\mathrm{supp}\,\# {\tilde{\mathbf{u}}}+\#\,\mathrm{supp}\,\varvec{\tilde{\theta }} +\varepsilon ^{-1/s}$ (dependent on $\varsigma$), such that

$$\begin{aligned} \left\| \mathbf{r}-\left[ \begin{array}{l} \mathbf{r}_1|_{\Lambda ^{H^1_0}} \\ \tilde{\mathbf{r}}_2|_{\Lambda ^{L_2}} \end{array}\right] \right\| \lesssim \varsigma (\Vert \tilde{u}'-\tilde{\theta }\Vert _{L_2(0,1)}+\Vert \tilde{\theta }'+f_\varepsilon \Vert _{H^{-1}(0,1)}) +\varepsilon \lesssim \varsigma \Vert \mathbf{r}\Vert +\varepsilon , \end{aligned}$$

where $\tilde{\mathbf{r}}_2$ is constructed from $\mathbf{r}_2$ by replacing $\mathbf{z}$ by $\mathbf{z}|_{\Lambda ^{\hat{H}^1_0}}$. This approximate residual evaluation satisfies the cost Condition (1.1) for optimality.

As we will see in Sect. 2, the advantage of the current construction of a first order system least squares problem is that it applies to *any* well-posed (semi-linear) second order PDE. The two instances of the spaces $H^1_0(0,1)$ represent the trial and test spaces in the standard variational formulation, and well-posedness of the latter implies well-posedness of the least squares formulation. The additional space $L_2(0,1)$ reads in general as an $L_2$-type space. So, in particular, with this formulation, $H({{\mathrm{div}}})$-spaces do not enter. The price to be paid is that (1.5) is somewhat more complicated than (1.2), and that therefore its approximation is somewhat more costly to compute.

#### Remark 1.3

The more popular ‘dual’ mixed formulation of our model problem reads as finding $(u,\theta ) \in L_2(0,1) \times H^1(0,1)$ such that $\langle -\theta ',v\rangle _{L_2(0,1)}+\langle \theta ,\eta \rangle _{L_2(0,1)}+\langle u,\eta '\rangle _{L_2(0,1)}=\langle f,v\rangle _{L_2(0,1)}$ ($(v,\eta ) \in L_2(0,1) \times H^1(0,1)$). The resulting least squares formulation has the combined disadvantages of both other formulations that we considered. It requires $f \in L_2(0,1)$, possibly nonlinear terms should map into $L_2(0,1)$, in more than one dimension the space $H^1(0,1)$ reads as an $H({{\mathrm{div}}})$-space, and one of the norms involved in the least squares minimalisation is a dual norm.

#### Remark 1.4

With the aim to avoid both a dual norm in the least squares minimalisation, and $H({{\mathrm{div}}})$ or other vectorial Sobolev spaces as trial spaces, in our first investigations of this least squares approach in [31], we considered the ‘extended div-grad’ first order system least squares formulation studied in [14]. A sufficient and necessary [30], but restrictive condition for its well-posedness is $H^2$-regularity of the homogeneous boundary value problem.

### Layout of the paper

In Sect. 2, a general procedure is given to reformulate *any* well-posed semi-linear 2nd order PDE as a well-posed first order least squares problem. As we will see, this procedure gives an effortless derivation of well-posed first order least squares formulations of elliptic 2nd order PDEs, and that of the stationary Navier–Stokes equations. The arising dual norm can be replaced by the equivalent $\ell _2$-norm of a functional in wavelet coordinates.

In Sect. 3, we recall properties of the adaptive wavelet Galerkin method (**awgm**). Operator equations of the form $F(z)=0$, where, for some Hilbert space $\mathscr {H}$, $F:\mathscr {H}\rightarrow \mathscr {H}'$ and *DF*(*z*) is symmetric and positive definite, are solved by the **awgm** at the best possible rate from a Riesz basis for $\mathscr {H}$. Furthermore, under a condition on the cost of the approximate residual evaluations, the method has optimal computational complexity.

In the short Sect. 4, it is shown that the **awgm** applies to the normal equations that result from the first order least squares problems as derived in Sect. 2.

In Sect. 5, we apply the **awgm** to a first order least squares formulation of a semi-linear 2nd order elliptic PDE with general inhomogeneous boundary conditions. Under a mild condition on the wavelet basis for the trial space, the efficient approximate residual evaluation that was outlined in Sect. 1.4 applies, and it satisfies the cost condition, so that the **awgm** is optimal. Wavelet bases that satisfy the assumptions are available on general polygonal domains. Some technical results needed for this section are given in “Appendix A”.

In Sect. 6 the findings from Sect. 5 are illustrated by numerical results.

In Sect. 7, we consider the so-called velocity–pressure–velocity gradient and the velocity–pressure–vorticity first order system formulations of the stationary Navier–Stokes equations. Results analogously to those demonstrated for the elliptic problem will be shown to be valid here as well.

## Reformulation of a semi-linear second order PDE as a first order system least squares problem

In an abstract framework, we give a procedure to write semi-linear second order PDEs, that have well-posed standard variational formulations, as a well-posed first order system least squares problems. A particular instance of this approach has been discussed in Sect. 1.4.

For some separable Hilbert spaces $\mathscr {U}$ and $\mathscr {V}$, for convenience over $\mathbb {R}$, consider a differentiable mapping

$$\begin{aligned} G:\mathscr {U}\supset \mathrm {dom}(G) \rightarrow \mathscr {V}'. \end{aligned}$$

### Remark 2.1

In applications *G* is the operator associated to a variational formulation of a PDO with trial space $\mathscr {U}$ and test space $\mathscr {V}$.

For $\mathscr {T}$ being another separable Hilbert space, let

2.1 $$\begin{aligned} G=G_0+G_1 G_2,\text { where } G_1 \in \mathcal {L}(\mathscr {T},\mathscr {V}'),\,G_2 \in \mathcal {L}(\mathscr {U},\mathscr {T}), \end{aligned}$$

i.e., $G_1$ and $G_2$ are bounded linear operators.

### Remark 2.2

In applications, as those discussed in Sects. 5 and  7, $G_1 G_2$ will be a factorization of the leading second order part of the PDO (possibly modulo terms that vanish at the solution, cf. Sect. 7.2) into a product of first order PDOs.

Obviously, *u* solves 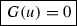 if and only if it is the first component of the solution $(u,\theta )$ of 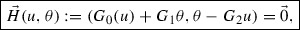 where $$\vec {H}:\mathscr {U}\times \mathscr {T}\supset \mathrm {dom}(G)\times \mathscr {T}=\mathrm {dom}(\vec {H}) \rightarrow \mathscr {V}' \times \mathscr {T}$$.

The following lemma shows that well-posedness of the original formulation implies that of the reformulation as a system.

### Lemma 2.3

Let $DG(u) \in \mathcal {L}(\mathscr {U},\mathscr {V}')$ be a homeomorphism with its range, i.e., $\Vert DG(u) v\Vert _{\mathscr {V}'} \eqsim \Vert v\Vert _\mathscr {U}$ $(v \in \mathscr {U})$. Then

$$\begin{aligned} D\vec {H}(u,\theta )=\left[ \begin{array}{@{}ll@{}} DG_0(u) &{} G_1 \\ -\,G_2 &{} I \end{array} \right] \in \mathcal {L}(\mathscr {U}\times \mathscr {T},\mathscr {V}'\times \mathscr {T}) \end{aligned}$$

is a homeomorphism with its range, being $\{(f,g) \in \mathscr {V}'\times \mathscr {T}:f-G_1g \in \mathrm{ran}\,DG(u)\}$. In particular, with $r, R>0$ such that $r \Vert v\Vert _\mathscr {U}\le \Vert DG(u)(v)\Vert _{\mathscr {V}'} \le R \Vert v\Vert _\mathscr {U}$, it holds that

2.2 $$\begin{aligned}&\Vert (v,\eta )\Vert _{\mathscr {U}\times \mathscr {T}}^2 \le \Big [\Big ({\textstyle \frac{1+\Vert G_2\Vert }{r}}\Big )^2+\Big (1+{\textstyle \frac{1+\Vert G_1\Vert (1+\Vert G_2\Vert )}{r}}\Big )^2\Big ] \Big \Vert D\vec {H}(u,\theta ) \left[ \begin{array}{@{}l@{}} v \\ \eta \end{array} \right] \Big \Vert _{\mathscr {V}' \times \mathscr {T}}^2, \end{aligned}$$

2.3 $$\begin{aligned}&\Big \Vert D\vec {H}(u,\theta ) \left[ \begin{array}{@{}l@{}} v \\ \eta \end{array} \right] \Big \Vert _{\mathscr {V}' \times \mathscr {T}}^2 \le \big ((R+(1+\Vert G_1\Vert )\Vert G_2\Vert )^2+(1+\Vert G_1\Vert )^2\big )\Vert (v,\eta )\Vert _{\mathscr {U}\times \mathscr {T}}^2. \end{aligned}$$

### Remark 2.4

Since $\mathrm{ran}\,DG(u)=\mathscr {V}'$ iff $\mathrm{ran}\,D\vec {H}(u,\theta )=\mathscr {V}'\times \mathscr {T}$, in particular we have that $DG(u) \in \mathcal {L}\mathrm {is}(\mathscr {U},\mathscr {V}')$ implies that $D\vec {H}(u,\theta ) \in \mathcal {L}\mathrm {is}(\mathscr {U}\times \mathscr {T},\mathscr {V}' \times \mathscr {T})$.

### Proof

We have $$D\vec {H}(u,\theta )\left[ \begin{array}{@{}l@{}} v \\ \eta \end{array} \right] =\left[ \begin{array}{@{}l@{}} DG_0(u)v+G_1\eta \\ \eta -G_2 v \end{array} \right] =\left[ \begin{array}{@{}l@{}} DG(u) v+G_1(\eta -G_2v) \\ \eta -G_2 v \end{array} \right] $$ by $DG_0(\cdot )=DG(\cdot )-G_1 G_2$. So

2.4 $$\begin{aligned} \mathrm{ran}\,D\vec {H}(u,\theta ) \subseteq \{(f,g) \in \mathscr {V}' \times \mathscr {T}:f-G_1g \in \mathrm{ran}\,DG(u)\}. \end{aligned}$$

By estimating $$\Vert D\vec {H}(u,\theta ) \left[ \begin{array}{@{}l@{}} v \\ \eta \end{array} \right] \Vert _{\mathscr {V}' \times \mathscr {T}} \le \Vert DG(u) v+G_1(\eta -G_2v)\Vert _{\mathscr {V}'}+\Vert \eta -G_2 v\Vert _\mathscr {T}$$, one easily arrives at (2.3).

For (*f*, *g*) being in the set at the right-hand side in (2.4), consider the system

$$\begin{aligned} D\vec {H}(u,\theta ) \left[ \begin{array}{@{}l@{}} v \\ \eta \end{array} \right] =\left[ \begin{array}{@{}l@{}} f \\ g \end{array} \right] \Longleftrightarrow \left\{ \begin{array}{@{}r@{}l@{}l@{}} DG_0(u) v+G_1 \eta \,=\, f \\ \eta -G_2 v \,=\, g \end{array} \right. \Longleftrightarrow \left\{ \begin{array}{@{}r@{}l@{}l@{}} DG(u) v \,=\, f-G_1 g \\ \eta \,=\, g+G_2 v \end{array} \right. . \end{aligned}$$

This system has a unique solution, so that the $\subseteq$-symbol in (2.4) reads as an equality sign, and $r\Vert v\Vert _\mathscr {U}\le \Vert f\Vert _{\mathscr {V}'}+\Vert G_1\Vert \Vert g\Vert _{\mathscr {T}}$ and $\Vert \eta \Vert _\mathscr {T}\le \Vert g\Vert _\mathscr {T}+\Vert G_2\Vert \Vert v\Vert _\mathscr {U}$. By estimating $\Vert (v,\eta )\Vert _{\mathscr {U}\times \mathscr {T}} \le \Vert v\Vert _\mathscr {U}+\Vert \eta \Vert _\mathscr {T}$ one easily arrives at (2.2). $\square$

In the following, we will always assume that

(i) there exists a solution *u* of $G(u)=0$;
(ii) *G* is two times continuously Fréchet differentiable in a neighborhood of *u*;
(iii) $DG(u) \in \mathcal {L}(\mathscr {U},\mathscr {V}')$ is a homeomorphism with its range.

Then

- $$(u,\theta )=(u,G_2 u)$$ solves $$\vec {H}(u,\theta )=\vec {0}$$;
- $\vec {H}$ is two times continuously Fréchet differentiable in a neighborhood of $(u,\theta )$;
- $D\vec {H}(u,\theta ) \in \mathcal {L}(\mathscr {U}\times \mathscr {T},\mathscr {V}' \times \mathscr {T})$ is a homeomorphism with its range,

the latter by Lemma 2.3. In summary, when the equation $G(u)=0$ is *well-posed* [(i)–(iii) are valid], then so is $\vec {H}(u,\theta )=\vec {0}$ [(a)–(c) are valid], and solving one equation is equivalent to solving the other.

### Remark 2.5

Actually, one might dispute whether these equations should be called well-posed when $\mathrm{ran}\,DG(u) \subsetneq \mathscr {V}'$ and so $\mathrm{ran}\,D\vec {H}(u,\theta ) \subsetneq \mathscr {V}' \times \mathscr {T}$. In any case, under conditions (i)–(iii), and so (a)–(c), the corresponding least-squares problems and resulting (nonlinear) normal equations *are* well-posed, as we will see next.

A solution $(u,\theta )$ of $\vec {H}(u,\theta )=\vec {0}$ is a minimizer of the least squares functional 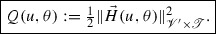 In particular, it holds that

### Lemma 2.6

For $\vec {H}:\mathscr {U}\times \mathscr {T}\supset \mathrm {dom}(\vec {H}) \rightarrow \mathscr {V}' \times \mathscr {T}$, and $\vec {H}$ being Fréchet differentiable at a root $(u,\theta )$, property (c) is equivalent to the property that for $(\tilde{u},\tilde{\theta }) \in \mathscr {U}\times \mathscr {V}$ in a neighbourhood of $(u,\theta )$,

$$\begin{aligned} Q(\tilde{u},\tilde{\theta }) \eqsim \Vert u-\tilde{u}\Vert ^2_{\mathscr {U}}+\Vert \theta -\tilde{\theta }\Vert ^2_{\mathscr {T}}. \end{aligned}$$

### Proof

This is a consequence of $\vec {H}(\tilde{u},\tilde{\theta })=D\vec {H}(u,\theta ) (\tilde{u}-u,\tilde{\theta }-\theta )+o(\Vert \tilde{u}-u\Vert _\mathscr {U}+\Vert \tilde{\theta }-\theta \Vert _\mathscr {T})$. $\square$

A minimizer $(u,\theta )$ of *Q* is a solution of the Euler-Lagrange equations 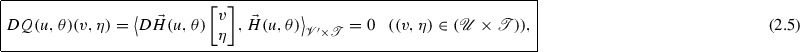
that, in this setting, are usually called (nonlinear) *normal equations*. Using (a)–(b), one computes that

$$\begin{aligned} D^2 Q(u,\theta )(v_1,\eta _1)(v_2,\eta _2)=\big \langle D\vec {H}(u,\theta ) \left[ \begin{array}{@{}l@{}} v_1 \\ \eta _1 \end{array} \right] , D\vec {H}(u,\theta ) \left[ \begin{array}{@{}l@{}} v_2 \\ \eta _2 \end{array} \right] \big \rangle _{\mathscr {V}' \times \mathscr {T}}. \end{aligned}$$

We conclude the following: Under the assumptions (a)–(c), it holds that

1. *DQ* is a mapping from a subset of a separable Hilbert space, viz. $\mathscr {U}\times \mathscr {T}$, to its dual;
2. there exists a solution of $DQ(u,\theta )=0$ (viz. any solution of $\vec {H}(u,\theta )=0$);
3. *DQ* is continuously Fréchet differentiable in a neighborhood of $(u,\theta )$;
4. $$0<D^2 Q(u,\theta )= D^2 Q(u,\theta )' \in \mathcal {L}\mathrm {is}(\mathscr {U}\times \mathscr {T},(\mathscr {U}\times \mathscr {T})')$$.

As a consequence of the last property, one infers that in a neighborhood of $(u,\theta )$, $DQ(u,\theta )=0$ has exactly one solution.

In view of the above findings, in order to solve $G(u)=0$, for a *G* that satisfies (i)–(iii), we are going to solve the (nonlinear) normal equations $DQ(u,\theta )=0$. A major advantage of *DQ* over *G* is that its derivative is symmetric and coercive.

A concern, however, is whether, for given $(u,\theta ), (v,\eta ) \in \mathscr {U}\times \mathscr {T}$, $DQ(u,\theta )(v,\eta )$ as given by (2.5) is evaluable. We will think of the inner product on $\mathscr {T}$ as being evaluable. In our applications, $\mathscr {T}$ will be of the form $L_2(\Omega )^N$. To deal with the dual norm on $\mathscr {V}'$, we equip $\mathscr {V}$ with a *Riesz basis*

$$\begin{aligned} \Psi ^\mathscr {V}=\{\psi _\lambda ^{\mathscr {V}}:\lambda \in \vee _\mathscr {V}\}, \end{aligned}$$

meaning that the *analysis operator*

$$\begin{aligned} \mathcal {F}_\mathscr {V}:g \mapsto [g(\psi ^\mathscr {V}_\lambda )]_{\lambda \in \vee _\mathscr {V}} \in \mathcal {L}\mathrm {is}(\mathscr {V}',\ell _2(\vee _\mathscr {V})), \end{aligned}$$

and so its adjoint, known as the *synthesis operator*,

$$\begin{aligned} \mathcal {F}_\mathscr {V}':\mathbf{v} \mapsto \mathbf{v}^\top \Psi ^\mathscr {V}:=\sum _{\lambda \in \vee _\mathscr {V}} v_\lambda \psi ^\mathscr {V}_\lambda \in \mathcal {L}\mathrm {is}(\ell _2(\vee _\mathscr {V}),\mathscr {V}). \end{aligned}$$

In the definition of the least squares functional *Q*, and consequently in that of *DQ*, we now *replace* the standard dual norm on $\mathscr {V}'$ by the *equivalent* norm $\Vert \mathcal {F}_\mathscr {V}\cdot \Vert _{\ell _2(\vee _\mathscr {V})}$. Then in view of the definition of $\vec {H}$ and the expression for $D\vec {H}$, we obtain that 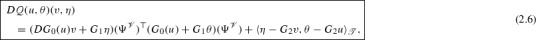 where (1)–(4) are still valid.

### Remark 2.7

We refer to [4] for an alternative approach to solve least square problems that involves dual norms.

To solve the obtained (nonlinear) normal equations $DQ(u,\theta )=0$ we are going to apply the adaptive wavelet Galerkin method (**awgm**). Note that the definition of $DQ(u,\theta )(v,\eta )$ still involves an infinite sum over $\vee _\mathscr {V}$ that later, inside the solution process, is going to be replaced by a finite one.

## The adaptive wavelet Galerkin method (**awgm**)

In this section, we summarize findings about the **awgm** from [17, 31]. Let

(I) $F:\mathscr {H}\supset \mathrm {dom}(F) \rightarrow \mathscr {H}'$, with $\mathscr {H}$ being a separable Hilbert space;
(II) $$F(z)=0$$;
(III) *F* be continuously differentiable in a neighborhood of *z*;
(IV) $$0<DF(z)=DF(z)' \in \mathcal {L}\mathrm {is}(\mathscr {H},\mathscr {H}')$$.

In our applications, the triple $(F,\mathscr {H},z)$ will read as $(DQ,\mathscr {U}\times \mathscr {T},(u,\theta ))$, so that (I)–(IV) are guaranteed by (1)–(4).

Let $\Psi =\{\psi _\lambda :\lambda \in \vee \}$ be a *Riesz basis* for $\mathscr {H}$, with analysis operator $\mathcal {F}:g \mapsto [g(\psi _\lambda )]_{\lambda \in \vee } \in \mathcal {L}\mathrm {is}(\mathscr {H},\ell _2(\vee ))$, and so synthesis operator $\mathcal {F}':\mathbf{v} \mapsto \mathbf{v}^\top \Psi :=\sum _{\lambda \in \vee } v_\lambda \psi _\lambda \in \mathcal {L}\mathrm {is}(\ell _2(\vee ),\mathscr {H}')$. For any $\Lambda \subset \vee$, we set

$$\begin{aligned} \ell _2(\Lambda ):=\{\mathbf{v}\in \ell _2(\vee ):\mathrm{supp}\,\mathbf{v} \subset \Lambda \}. \end{aligned}$$

For satisfying the forthcoming Condition 3.5 that concerns the computational cost, it will be relevant that $\Psi$ is a basis of *wavelet* type.

Writing $z=\mathcal {F}' \mathbf{z}$, and with

$$\begin{aligned} \mathbf{F}:=\mathcal {F}F \mathcal {F}':\ell _2(\vee ) \rightarrow \ell _2(\vee ), \end{aligned}$$

an *equivalent* formulation of $F(z)=0$ is given by

$$\begin{aligned} \mathbf{F}(\mathbf{z})=0. \end{aligned}$$

We are going to approximate $\mathbf{z}$, and so *z*, by a sequence of Galerkin approximations from the spans of increasingly larger sets of wavelets, which sets are created by an adaptive process. Given $\Lambda \subset \vee$, the Galerkin approximation $\mathbf{z}_\Lambda$, or equivalently, $z_\Lambda :=\mathbf{z}_\Lambda ^\top \Psi$, are the solutions of $\langle \mathbf{F}(\mathbf{z}_\Lambda ),\mathbf{v}_\Lambda \rangle _{\ell _2(\vee )}=0$ ($\mathbf{v}_\Lambda \in \ell _2(\Lambda )$), i.e., $\mathbf{F}(\mathbf{z}_\Lambda )|_{\Lambda }=0$, and $F(z_\Lambda )(v_\Lambda )=0$ ($v_\Lambda \in {{\mathrm{span}}}\{\psi _\lambda :\lambda \in \Lambda \}$), respectively. These solutions exist uniquely when $\inf _{{\tilde{\mathbf{z}}}_\Lambda \in \ell _2(\Lambda )} \Vert \mathbf{z}-{\tilde{\mathbf{z}}}_\Lambda \Vert$ is sufficiently small [26, 31].

In order to be able to construct efficient algorithms, in particular when *F* is non-affine, it will be needed to consider only sets $\Lambda$ from a certain subset of all finite subsets of $\vee$. In our applications, this collection of so-called *admissible* $\Lambda$ will consist of (Cartesian products of) finite *trees*. For the moment, it suffices when the collection of admissible sets is such that the union of any two admissible sets is again admissible.

To provide a benchmark to evaluate our adaptive algorithm, for $s>0$, we define the nonlinear *approximation class*

3.1 $$\begin{aligned} \begin{aligned}&\mathcal {A}^s:=\Big \{\mathbf{z} \in \ell _2(\vee ):\Vert \mathbf{z}\Vert _{\mathcal {A}^s}\\&\quad :=\sup _{\varepsilon >0} \varepsilon \times \min \big \{(\# \Lambda )^s:\Lambda \text { is admissible, }\inf _{{\tilde{\mathbf{z}}} \in \ell _2(\Lambda )} \Vert \mathbf{z}-{\tilde{\mathbf{z}}}\Vert \le \varepsilon \big \}<\infty \Big \}. \end{aligned} \end{aligned}$$

A vector $\mathbf{z}$ is in $\mathcal {A}^s$ if and only if there exists a sequence of admissible $(\Lambda _i)_i$, with $\lim _{i \rightarrow \infty } \#\Lambda _i=\infty$, such that $\sup _i \inf _{\mathbf{z}_i \in \ell _2(\Lambda _i)} (\#\Lambda _i)^s \Vert \mathbf{z}-\mathbf{z}_i\Vert <\infty$. This means that $\mathbf{z}$ *can* be approximated in $\ell _2(\vee )$ at rate *s* by vectors supported on admissible sets, or, equivalently, *z* can be approximated in $\mathscr {H}$ at rate *s* from spaces of type ${{\mathrm{span}}}\{\psi _\lambda :\lambda \in \Lambda ,\,\Lambda \text { is admissible}\}$.

The adaptive wavelet Galerkin method (**awgm**) defined below produces a sequence of increasingly more accurate Galerkin approximations $\mathbf{z}_\Lambda$ to $\mathbf{z}$. The, generally, infinite residual $\mathbf{F}(\mathbf{z}_\Lambda )$ is used as an a posteriori error estimator. A motivation for the latter is given by the following result.

### Lemma 3.1

For $\Vert \mathbf{z}-{\tilde{\mathbf{z}}}\Vert$ sufficiently small, it holds that $\Vert \mathbf{F}({\tilde{\mathbf{z}}})\Vert \eqsim \Vert \mathbf{z}-{\tilde{\mathbf{z}}}\Vert$.

### Proof

With $\tilde{z}={\tilde{\mathbf{z}}}^\top \Psi$, it holds that $\Vert \mathbf{F}({\tilde{\mathbf{z}}})\Vert \eqsim \Vert F(\tilde{z})\Vert _{\mathscr {H}'}$. From (II)–(III), we have $F(\tilde{z})=DF(z)(\tilde{z}-z)+o(\Vert \tilde{z}-z\Vert _\mathscr {H})$. The proof is completed by $\Vert DF(z)(\tilde{z}-z)\Vert _{\mathscr {H}'} \eqsim \Vert \tilde{z}-z\Vert _\mathscr {H}$ by (IV). $\square$

This a posteriori error estimator guides an appropriate enlargement of the current set $\Lambda$ using a bulk chasing strategy, so that the sequence of approximations converge with the best possible rate to $\mathbf{z}$. To arrive at an implementable method, that is even of optimal computational complexity, both the Galerkin solution and its residual are allowed to be computed inexactly within sufficiently small relative tolerances.

### Algorithm 3.2

(awgm) 
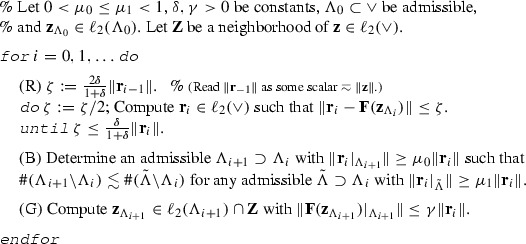


In step (R), by means of a loop in which an absolute tolerance is decreased, the true *residual* $\mathbf{F}(\mathbf{z}_{\Lambda _i})$ is approximated within a relative tolerance $\delta$. In step (B), *bulk chasing* is performed on the approximate residual. The idea is to find a smallest admissible $\Lambda _{i+1} \supset \Lambda _i$ with $\Vert \mathbf{r}_i|_{\Lambda _{i+1}}\Vert \ge \mu _0 \Vert \mathbf{r}_i\Vert$. In order to be able to find an implementation that is of linear complexity, the condition of having a truly smallest $\Lambda _{i+1}$ has been relaxed. Finally, in step (G), a sufficiently accurate approximation of the *Galerkin* solution w.r.t. the new set $\Lambda _{i+1}$ is determined.

*Convergence* of the adaptive wavelet Galerkin method, with the *best possible rate*, is stated in the following theorem.

### Theorem 3.3

[31, Thm. 3.9] Let $\mu _1, \gamma , \delta$, $\inf _{\mathbf{v}_{\Lambda _0} \in \ell _2(\Lambda _0)}\Vert \mathbf{z}-\mathbf{v}_{\Lambda _0}\Vert$, $\Vert \mathbf{F}(\mathbf{z}_{\Lambda _0})|_{\Lambda _0}\Vert$, and the neighborhood $\mathbf{Z}$ of the solution $\mathbf{z}$ all be sufficiently small. Then, for some $\alpha =\alpha [\mu _0]<1$, the sequence $(\mathbf{z}_{\Lambda _i})_i$ produced by **awgm** satisfies

$$\begin{aligned} \Vert \mathbf{z}-\mathbf{z}_{\Lambda _{i}}\Vert \lesssim \alpha ^{i} \Vert \mathbf{z}-\mathbf{z}_{\Lambda _0}\Vert . \end{aligned}$$

If, for whatever $s>0$, $\mathbf{z} \in \mathcal {A}^s$, then $\# (\Lambda _{i+1} {\setminus } \Lambda _0) \lesssim \Vert \mathbf{z}-\mathbf{z}_{\Lambda _{i}}\Vert ^{-1/s}$.

The *computation* of the approximate Galerkin solution $\mathbf{z}_{\Lambda _{i+1}}$ can be implemented by performing the simple fixed point iteration

3.2 $$\begin{aligned} \mathbf{z}_{\Lambda _{i+1}}^{(j+1)}=\mathbf{z}_{\Lambda _{i+1}}^{(j)}-\omega \mathbf{F}(\mathbf{z}_{\Lambda _{i+1}}^{(j)})|_{\Lambda _{i+1}}. \end{aligned}$$

Taking $\omega >0$ to be a sufficiently small constant and starting with $\mathbf{z}_{\Lambda _{i+1}}^{(0)}=\mathbf{z}_{\Lambda _{i}}$, a fixed number of iterations suffices to meet the condition $\Vert \mathbf{F}(\mathbf{z}^{(j+1)}_{\Lambda _{i+1}})|_{\Lambda _{i+1}}\Vert \le \gamma \Vert \mathbf{r}_i\Vert$. This holds also true when each of the $\mathbf{F}()|_{\Lambda _{i+1}}$ evaluations is performed within an absolute tolerance that is a sufficiently small fixed multiple of $\Vert \mathbf{r}_i\Vert$.

Optimal *computational* complexity of the **awgm**—meaning that the work to obtain an approximation within a given tolerance $\varepsilon >0$ can be bounded on some constant multiple of the bound on its support length from Theorem 3.3—is guaranteed under the following two conditions concerning the cost of the “bulk chasing” process, and that of the approximate residual evaluation, respectively. Indeed, apart from some obvious computations, these are the only two tasks that have to be performed in **awgm**.

### Condition 3.4

The determination of $\Lambda _{i+1}$ in Algorithm 3.2 is performed in ${\mathcal {O}}(\#\,\mathrm{supp}\,\mathbf{r}_i+\# \Lambda _i)$ operations.

In case of unconstrained approximation, i.e., any finite $\Lambda \subset \vee$ is admissible, this condition is satisfied by collecting the largest entries in modulus of $\mathbf{r}_i$, where, to avoid a suboptimal complexity, an exact sorting should be replaced by an approximate sorting based on binning. With tree approximation, the condition is satisfied by the application of the so-called *Thresholding Second Algorithm* from [2]. We refer to [31, §3.4] for a discussion.

To understand the second condition, that in the introduction was referred to as the cost Condition (1.1), note that inside the **awgm** it is never needed to approximate a residual more accurately than within a sufficiently small, but fixed relative tolerance.

### Condition 3.5

For a sufficiently small, fixed $\varsigma >0$, there exists a neighborhood $\mathbf{Z}$ of the solution $\mathbf{z}$ of $\mathbf{F}(\mathbf{z})=0$, such that for all admissible $\Lambda \subset \vee$, ${\tilde{\mathbf{z}}}\in \ell _2(\Lambda ) \cap \mathbf{Z}$, and any $\varepsilon >0$, there exists an $\mathbf{r} \in \ell _2(\vee )$ with

$$\begin{aligned} \Vert \mathbf{F}({\tilde{\mathbf{z}}})-\mathbf{r}\Vert \le \varsigma \Vert \mathbf{F}({\tilde{\mathbf{z}}})\Vert + \varepsilon , \end{aligned}$$

that one can compute in ${\mathcal {O}}(\varepsilon ^{-1/s}+\#\Lambda )$ operations. Here $s>0$ is such that $\mathbf{z} \in \mathcal {A}^s$.

Under both conditions, the **awgm** has optimal computational complexity:

### Theorem 3.6

In the setting of Theorem 3.3, and under Conditions 3.4 and 3.5, not only $\# \mathbf{z}_{\Lambda _i}$, but also the number of arithmetic operations required by **awgm** for the computation of $\mathbf{z}_{\Lambda _i}$ is ${\mathcal {O}}(\Vert \mathbf{z}-\mathbf{z}_{\Lambda _i}\Vert ^{-1/s})$.

## Application to normal equations

As discussed in Sect. 2, we will apply the **awgm** to the (nonlinear) normal equations $DQ(u,\theta )=0$, with *DQ* from (2.6). That is, we apply the findings collected in the previous section for the general triple $(F,\mathscr {H},z)$ now reading as $(DQ,\mathscr {U}\times \mathscr {T},(u,\theta ))$.

For $\Psi ^\mathscr {U}=\{\psi _\lambda ^\mathscr {U}:\lambda \in \vee _\mathscr {U}\}$ and $\Psi ^\mathscr {T}=\{\psi _\lambda ^\mathscr {T}:\lambda \in \vee _\mathscr {T}\}$ being Riesz bases for $\mathscr {U}$ and $\mathscr {T}$, respectively, we equip $\mathscr {U}\times \mathscr {T}$ with Riesz basis

4.1 $$\begin{aligned} \Psi =\{\psi _\lambda :\lambda \in \vee :=\vee _\mathscr {U}\cup \vee _\mathscr {T}\}:=(\Psi ^\mathscr {U},0_\mathscr {T}) \cup (0_\mathscr {U},\Psi ^\mathscr {T}) \end{aligned}$$

(w.l.o.g. we assume that $\vee _\mathscr {U}\cap \vee _\mathscr {T}=\emptyset$). With $\mathcal {F}\in \mathcal {L}\mathrm {is}(\mathscr {U}\times \mathscr {T},\ell _2(\vee ))$ being the corresponding analysis operator, and $D\mathbf{Q}:=\mathcal {F}DQ \mathcal {F}'$, for $[{\tilde{\mathbf{u}}}^\top ,\varvec{\tilde{\theta }}^\top ]^\top \in \ell _2(\vee )$, and with $(\tilde{u},\tilde{\theta }):=[{\tilde{\mathbf{u}}}^\top ,\varvec{\tilde{\theta }}^\top ]\Psi$, we have

4.2 $$\begin{aligned} \begin{aligned} D\mathbf{Q}([{\tilde{\mathbf{u}}}^\top ,\varvec{\tilde{\theta }}^\top ]^\top ) =&\left[ \begin{array}{@{}l@{}} DG_0(\tilde{u}) (\Psi ^\mathscr {U})(\Psi ^\mathscr {V})^\top \\ G_1 (\Psi ^\mathscr {T})(\Psi ^\mathscr {V})^\top \end{array} \right] \big (G_0(\tilde{u})+ G_1 \tilde{\theta } \big ) (\Psi ^\mathscr {V})\\&+\,\left\langle \left[ \begin{array}{@{}l@{}} - G_2(\Psi ^\mathscr {U}) \\ \Psi ^\mathscr {T}\end{array} \right] ,\tilde{\theta }-G_2 \tilde{u}\right\rangle _\mathscr {T}. \end{aligned} \end{aligned}$$

In this setting, using Lemma 3.1, Condition 3.5 can be reformulated as follows:

### Condition 3.5*

For a sufficiently small, fixed $\varsigma >0$, there exists a neighborhood of the solution $(u,\theta )$ of $DQ(u,\theta )=0$, such that for all admissible $\Lambda \subset \vee$, all $[{\tilde{\mathbf{u}}}^\top ,\varvec{\tilde{\theta }}^\top ]^\top \in \ell _2(\Lambda )$, with $(\tilde{u},\tilde{\theta }):=[{\tilde{\mathbf{u}}}^\top ,\varvec{\tilde{\theta }}^\top ] \Psi$ being in this neighborhood, and any $\varepsilon >0$, there exists an $\mathbf{r} \in \ell _2(\vee )$ with

$$\begin{aligned} \Vert D\mathbf{Q}([{\tilde{\mathbf{u}}}^\top ,\varvec{\tilde{\theta }}^\top ]^\top )-\mathbf{r}\Vert \le \varsigma (\Vert u-\tilde{u}\Vert _\mathscr {U}+\Vert \theta -\tilde{\theta }\Vert _\mathscr {T}) + \varepsilon , \end{aligned}$$

that one can compute in ${\mathcal {O}}(\varepsilon ^{-1/s}+\#\Lambda )$ operations, where $s>0$ is such that $[\mathbf{u}^\top , \mathbf{\theta }^\top ]^\top \in \mathcal {A}^s$.

To verify this condition, we will use the additional property, i.e. on top of (1)–(4), that $\Vert u-\tilde{u}\Vert _\mathscr {U}+\Vert \theta -\tilde{\theta }\Vert _\mathscr {T}\eqsim \Vert G_0(\tilde{u})-G_1\tilde{\theta }\Vert _{\mathscr {V}'}+\Vert \tilde{\theta }-G_2\tilde{u}\Vert _{\mathscr {T}}$, which is provided by Lemma 2.6.

## Semi-linear 2nd order elliptic PDE

We apply the solution method outlined in Sects. 2, 3 and 4 to the example of a semi-linear 2nd order elliptic PDE with general (inhomogeneous) boundary conditions. The main task will be to verify Condition 3.5*.

### Reformulation as a first order system least squares problem

Let $\Omega \subset \mathbb {R}^n$ be a bounded domain, $\Gamma _N \cup \Gamma _D=\partial \Omega$ with ${{\mathrm{meas}}}(\Gamma _N \cap \Gamma _D)=0$, ${{\mathrm{meas}}}(\Gamma _D)>0$ when $\Gamma _D \ne \emptyset$, and $A:\Omega \rightarrow \mathbb {R}^{n\times n}_\mathrm{symm}$ with $\xi ^\top A(\cdot ) \xi \eqsim \Vert \xi \Vert ^2$ ($\xi \in \mathbb {R}^n$, a.e.). We set 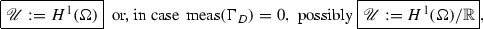
and 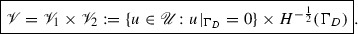
For $N:\mathscr {U}\supset \mathrm {dom}(N) \rightarrow \mathscr {V}_1'$, $f \in \mathscr {V}_1'$, $g \in H^{\frac{1}{2}}(\Gamma _D)$, and $h \in H^{-\frac{1}{2}}(\Gamma _N)$, we consider the semi-linear boundary value problem5.1that in standard variational form reads as finding $u \in \mathscr {U}$ such that

$$\begin{aligned} (Gu)(v) :=\int _\Omega A \nabla u \cdot \nabla v_1 + (N(u)-f) v_1 dx-\int _{\Gamma _N} h v_1 \,ds +\int _{\Gamma _D} (u-g)v_2 \,ds=0 \end{aligned}$$

$$(v=(v_1,v_2) \in \mathscr {V})$$.

We assume that this variational problem has a solution *u*, and that *G*, i.e., *N*, is two times continuously Fréchet differentiable in a neighborhood of *u*, and $DG(u) \in \mathcal {L}(\mathscr {U},\mathscr {V}')$ is a homeomorphism with its range, i.e., we *assume* that

$$\begin{aligned} G:\mathscr {U}\supset \mathrm {dom}(G) \rightarrow \mathscr {V}' \text { satisfies } \text { (i)}{-}\text { (iii)} \end{aligned}$$

formulated in Sect. 2.

#### Remarks 5.1

Because $\mathscr {U}\rightarrow H^{\frac{1}{2}}(\Gamma _D):u \mapsto u|_{\partial \Omega }$ is surjective, from [30, Thm. 2.1] it follows that condition (iii) is satisfied when

$$\begin{aligned} L:= w \mapsto \Big (v_1 \mapsto \int _\Omega A \nabla w \cdot \nabla v_1+ DN(u)(w) v_1\,dx\Big ) \in \mathcal {L}\mathrm {is}(\mathscr {V}_1,\mathscr {V}_1') \end{aligned}$$

(actually, *L* being a homeomorphism with its range is already sufficient).

By writing $g=u_0|_{\Gamma _D}$ for some $u_0 \in \mathscr {U}$, one infers that for linear *N*, existence of a (unique) solution *u*, i.e. (i), follows from $L \in \mathcal {L}\mathrm {is}(\mathscr {V}_1,\mathscr {V}_1')$. For $g=0$, the conditions of *N* being monotone and locally Lipschitz are sufficient for having a (unique) solution *u*. Relaxed conditions on *N* suffice to have a (locally unique) solution. We refer to [5].

Using the framework outlined in Sect. 2, we write this second order elliptic PDE as a first order system least squares problem. Putting 
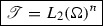
, we define

$$\begin{aligned} G_1 \in \mathcal {L}(\mathscr {T},\mathscr {V}'),\qquad G_2 \in \mathcal {L}(\mathscr {U},\mathscr {T}), \end{aligned}$$

by

$$\begin{aligned} G_2 u=A \nabla u,\qquad (G_1 \vec {\theta })(v_1,v_2)=\int _\Omega \vec {\theta } \cdot \nabla v_1 \,d x. \end{aligned}$$

The results from Sect. 2 show that the solution *u* can be found as the first component of the minimizer $(u,\vec {\theta }) \in \mathscr {U}\times \mathscr {T}$ of

5.2 $$\begin{aligned} \begin{aligned} Q(u,\vec {\theta })&:={\textstyle \frac{1}{2}}\Big (\big \Vert v_1 \mapsto \int _\Omega \vec {\theta } \cdot \nabla v_1 +(N(u)-f)v_1\,dx -\int _{\Gamma _N} v_1 h \,ds\big \Vert _{\mathscr {V}_1'}^2\\&\qquad +\,\Vert \vec {\theta }-A \nabla u\Vert _{L_2(\Omega )^n}^2+\Vert u-g\Vert ^2_{\mathscr {V}_2'}\Big ), \end{aligned} \end{aligned}$$

being the solution of the normal equations $DQ(u,\vec {\theta })=0$, and furthermore, that these normal equations are well-posed in the sense that they satisfy (1)–(4).

To deal with the ‘unpractical’ norm on $\mathscr {V}'$, as in Sect. 1.4, at the end of Sect. 2, and in Sect. 4, we equip $\mathscr {V}_1$ and $\mathscr {V}_2$ with wavelet *Riesz bases*

$$\begin{aligned} \Psi ^{\mathscr {V}_1}=\{\psi _\lambda ^{\mathscr {V}_1}:\lambda \in \vee _{\mathscr {V}_1}\}, \quad \Psi ^{\mathscr {V}_2}=\{\psi _\lambda ^{\mathscr {V}_2}:\lambda \in \vee _{\mathscr {V}_2}\}, \end{aligned}$$

and replace, in the definition of *Q*, the norms on their duals by the equivalent norms defined by $\Vert g(\Psi ^{\mathscr {V}_1})\Vert$ or $\Vert g(\Psi ^{\mathscr {V}_2})\Vert$, for $g \in \mathscr {V}_1'$ or $g \in \mathscr {V}_2'$, respectively.

Next, after equiping $\mathscr {U}$ and $\mathscr {T}$ with *Riesz bases*

$$\begin{aligned} \Psi ^{\mathscr {U}}=\{\psi _\lambda ^{\mathscr {U}}:\lambda \in \vee _{\mathscr {U}}\}, \quad \Psi ^{\mathscr {T}}=\{\psi _\lambda ^{\mathscr {T}}:\lambda \in \vee _{\mathscr {T}}\}, \end{aligned}$$

and so $\mathscr {U}\times \mathscr {T}$ with $\Psi =(\Psi ^{\mathscr {U}},0_{\mathscr {T}}) \cup (0_{\mathscr {U}},\Psi ^{\mathscr {T}})$, we apply the **awgm** to the resulting system

$$\begin{aligned} D\mathbf{Q}([\mathbf{{u}}^\top ,\varvec{{\theta }}^\top ]^\top )= & {} \left\langle \left[ \begin{array}{@{}l@{}} A \nabla \Psi ^{\mathscr {U}}\\ -\Psi ^{\mathscr {T}} \end{array} \right] ,A \nabla {u} -\vec {{\theta }}\right\rangle _{L_2(\Omega )^n} \\&+\,\left[ \begin{array}{@{}l@{}} \langle \Psi ^{\mathscr {U}}, \Psi ^{\mathscr {V}_2} \rangle _{L_2(\Gamma _D)}\\ 0_{\vee _\mathscr {T}}\end{array} \right] \langle \Psi ^{\mathscr {V}_2}, {u}-g\rangle _{L_2(\Gamma _D)}\\&+\,\left[ \begin{array}{@{}l@{}} \langle DN({u}) \Psi ^{\mathscr {U}}, \Psi ^{\mathscr {V}_1} \rangle _{L_2(\Omega )}\\ \langle \Psi ^{\mathscr {T}},\nabla \Psi ^{\mathscr {V}_1} \rangle _{L_2(\Omega )^n} \end{array} \right] \\&\Big \{\big \langle \Psi ^{\mathscr {V}_1}, N({u})-f\big \rangle _{L_2(\Omega )}-\big \langle \Psi ^{\mathscr {V}_1},h \big \rangle _{L_2(\Gamma _N)} +\langle \nabla \Psi ^{\mathscr {V}_1}, \vec {{\theta }} \rangle _{L_2(\Omega )^n} \Big \}=0, \end{aligned}$$

where $$(u,\vec {{\theta }}):=[\mathbf{{u}}^\top ,\varvec{{\theta }}^\top ] \Psi $$.

To express the three terms in $v\mapsto \langle v, N({u})-f\rangle _{L_2(\Omega )}-\langle v,h \rangle _{L_2(\Gamma _N)} +\langle \nabla v, \vec {{\theta }} \rangle _{L_2(\Omega )^n} \in \mathscr {V}_1'$ w.r.t. one dictionary of functions on $\Omega$ and one dictionary of functions on $\Gamma _N$, similarly to Sect. 1.4 we impose the additional, but in applications easily realisable condition that

5.3 $$\begin{aligned} \Psi ^{\mathscr {T}} \subset H({{\mathrm{div}}};\Omega ). \end{aligned}$$

Then for finitely supported approximations $[{\tilde{\mathbf{u}}}^\top ,\varvec{\tilde{\theta }}^\top ]^\top$ to $[\mathbf{{u}}^\top ,\varvec{{\theta }}^\top ]^\top$, for $(\tilde{u},\vec {\tilde{\theta }}):=[{\tilde{\mathbf{u}}}^\top ,\varvec{\tilde{\theta }}^\top ] \Psi \in \mathscr {U}\times H({{\mathrm{div}}};\Omega )$, we have 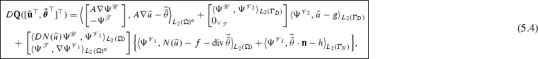
where we used the vanishing traces of $v \in \mathscr {V}_1$ at $\Gamma _D$, to write $\langle \nabla v, \vec {\tilde{\theta }}\rangle _{L_2(\Omega )^n}$ as $\langle v,- {{\mathrm{div}}}\vec {\tilde{\theta }}\rangle _{L_2(\Omega )}+\langle v, \vec {\tilde{\theta }}\cdot \mathbf{n}\rangle _{L_2(\Gamma _N)}$.

Each of the terms $A \nabla {\tilde{u}} -\vec {\tilde{\theta }}$, ${\tilde{u}}-g$, $N({\tilde{u}})-f-{{\mathrm{div}}}\vec {\tilde{\theta }}$, and $\vec {\tilde{\theta }}\cdot \mathbf{n}-h$ correspond, in strong form, to a term of the least squares functional, and therefore their norms can be bounded by a multiple of the norm of the residual, which is the basis of our approximate residual evaluation. In order to verify Condition 3.5*, we have to collect some assumptions on the wavelets, which will be done in the next subsection.

#### Remark 5.2

If $\Gamma _D=\emptyset$, then obviously (5.4) should be read without the second term involving $\Psi ^{\mathscr {V}_2}$. If $\Gamma _D\ne \emptyset$ and *homogeneous* Dirichlet boundary conditions are prescribed on $\Gamma _D$, i.e., $g=0$, it is simpler to select $\mathscr {U}=\mathscr {V}_1=\{u \in H^1(\Omega ):u|_{\Gamma _D}=0\}$, and to omit integral over $\Gamma _D$ in the definition of *G*, so that again (5.4) should be read without the second term involving $\Psi ^{\mathscr {V}_2}$.

### Wavelet assumptions and definitions

We formulate conditions on $\Psi ^{\mathscr {V}_1}$, $\Psi ^{\mathscr {V}_2}$, $\Psi ^{\mathscr {U}}$, and $\Psi ^{\mathscr {T}}$, in addition to being Riesz bases for $\mathscr {V}_1$, $\mathscr {V}_2$, $\mathscr {U}$, and $\mathscr {T}$, respectively.

Recalling that $\mathscr {T}=\mathscr {T}_1\times \cdots \times \mathscr {T}_n$, we select $\Psi ^\mathscr {T}$ of canonical form

$$\begin{aligned} (\Psi ^{\mathscr {T}_1},0_{\mathscr {T}_2},\ldots ,0_{\mathscr {T}_n}) \cup \cdots \cup (0_{\mathscr {T}_1},\ldots ,0_{\mathscr {T}_{n-1}},\Psi ^{\mathscr {T}_n}), \end{aligned}$$

where $\Psi ^{\mathscr {T}_q}=\{\psi ^{\mathscr {T}_q}_\lambda :\lambda \in \vee _{\mathscr {T}_q}\}$ is a Riesz basis for $\mathscr {T}_q$ (with $\vee _{\mathscr {T}_{q'}} \cap \vee _{\mathscr {T}_{q''}} =\emptyset$ when $q'\ne q''$).

For $*\in \{\mathscr {U},\mathscr {T}_1,\ldots ,\mathscr {T}_n,\mathscr {V}_1\}$, we collect a number of (standard) assumptions, ($w_{1}$)–($w_{4}$), on the scalar-valued wavelet collections $\Psi ^*=\{\psi _\lambda ^*:\lambda \in \vee _*\}$ on $\Omega$. Corresponding assumptions on the wavelets $\Psi ^{\mathscr {V}_2}$ on $\Gamma _D$ will be formulated at the end of this subsection. To each $\lambda \in \vee _*$, we associate a value $|\lambda | \in \mathbb {N}_0$, which is called the *level* of $\lambda$. We will assume that the elements of $\Psi ^*$ have *one vanishing moment*, and are *locally supported, piecewise polynomial* of some degree *m*, w.r.t. *dyadically nested partitions* in the following sense:

($$w_{1}$$)
There exists a collection $\mathcal {O}_\Omega :=\{\omega :\omega \in \mathcal {O}_\Omega \}$ of closed polytopes, such that, with $|\omega |\in \mathbb {N}_0$ being the *level* of $\omega$, ${{\mathrm{meas}}}(\omega \cap \omega ')=0$ when $|\omega |=|\omega '|$ and $\omega \ne \omega '$; for any $\ell \in \mathbb {N}_0$, $\bar{\Omega }=\cup _{|\omega |=\ell } \omega$; $\mathrm{diam}\,\omega \eqsim 2^{-|\omega |}$; and $\omega$ is the union of $\omega '$ for some $\omega '$ with $|\omega '|=|\omega |+1$. We call $\omega$ the *parent* of its *children* $\omega '$. Moreover, we assume that the $\omega \in \mathcal {O}_\Omega$ are uniformly *shape regular*, in the sense that they satisfy a uniform Lipschitz condition, and ${{\mathrm{meas}}}(F_\omega )\eqsim {{\mathrm{meas}}}(\omega )^{\frac{n-1}{n}}$ for $F_\omega$ being any facet of $\omega$.
($$w_{2}$$)
$\mathrm{supp}\,\psi ^*_\lambda$ is contained in a connected union of a uniformly bounded number of $\omega$’s with $|\omega |=|\lambda |$, and restricted to each of these $\omega$’s is $\psi ^*_\lambda$ a polynomial of degree *m*.
($$w_{3}$$)
Each $\omega$ is intersected by the supports of a uniformly bounded number of $\psi ^*_\lambda$’s with $|\lambda |=|\omega |$.
($$w_{4}$$)
$\int _\Omega \psi ^*_\lambda \,dx =0$, possibly with the exception of those $\lambda$ with $\mathrm{dist}(\mathrm{supp}\,\psi ^*_\lambda ,\Gamma _D) \lesssim 2^{-|\lambda |}$, or with $|\lambda |=0$.

Generally, the polynomial degree *m* will be different for the different bases, but otherwise fixed. The collection $\mathcal {O}_\Omega$ is shared among all bases. Note that the conditions of $\Psi ^\mathscr {U}$ being a basis for $\mathscr {U}$, and to consist of piecewise polynomials, implies that $\mathscr {U}\subset C(\bar{\Omega })$. Wavelets of in principle arbitrary order that satisfy these assumptions can be found in e.g. [20, 25].

#### Definition 5.3

A collection ${\mathcal {T}} \subset \mathcal {O}_\Omega$ such that $\overline{\Omega }=\cup _{\omega \in {\mathcal {T}}} \omega$, and for $\omega _1 \ne \omega _2 \in {\mathcal {T}}$, ${{\mathrm{meas}}}(\omega _1 \cap \omega _2)=0$ will be called a *tiling*. With $\mathcal {P}_m(\mathcal {T})$, we denote the space of piecewise polynomials of degree *m* w.r.t. $\mathcal {T}$. The smallest common refinement of tilings ${\mathcal {T}}_1$ and ${\mathcal {T}}_2$ is denoted as ${\mathcal {T}}_1 \oplus {\mathcal {T}}_2$.

To be able to find, in linear complexity, a representation of a function, given as linear combination of wavelets, as a piecewise polynomial w.r.t. a tiling—mandatory for an efficient evaluation of nonlinear terms, we will impose a tree constraint on the underlying set of wavelet indices. A similar approach was followed earlier in [6, 10, 21, 33, 35].

#### Definition 5.4

To each $\lambda \in \vee _*$ with $|\lambda |>0$, we associate one $\mu \in \vee _*$ with $|\mu |=|\lambda |-1$ and ${{\mathrm{meas}}}(\mathrm{supp}\,\psi ^*_\lambda \cap \mathrm{supp}\,\psi ^*_\mu )>0$. We call $\mu$ the *parent* of $\lambda$, and so $\lambda$ a *child* of $\mu$.

To each $\lambda \in \vee _*$, we associate some neighbourhood $\mathcal {S}(\psi ^*_\lambda )$ of $\mathrm{supp}\,\psi ^*_\lambda$, with diameter $\lesssim 2^{-|\lambda |}$, such that $\mathcal {S}(\psi ^*_\lambda ) \subset \mathcal {S}(\psi ^*_\mu )$ when $\lambda$ is a child of $\mu$.

We call a finite $\Lambda \subset \vee _*$ a *tree*, if it contains all $\lambda \in \vee _*$ with $|\lambda |=0$, as well as the parent of any $\lambda \in \Lambda$ with $|\lambda |>0$.

Note that we now have tree structures on the set $\mathcal {O}_\Omega$ of polytopes, and as well as on the wavelet index sets $\vee _*$. We trust that no confusion will arise when we speak about parents or children.

For some collections of wavelets, as the Haar or more generally, Alpert wavelets [1], it suffices to take $\mathcal {S}(\psi ^*_\lambda ):=\mathrm{supp}\,\psi ^*_\lambda$. The next result shows that, thanks to ($w_{1}$)-($w_{2}$), a suitable neighbourhood $\mathcal {S}(\psi ^*_\lambda )$ as meant in Definition 5.4 always exists.

#### Lemma 5.5

With $C:=\sup _{\lambda \in \vee _*} 2^{|\lambda |} {{\mathrm{diam}}}\, \mathrm{supp}\,\psi ^*_\lambda$, a valid choice of $\mathcal {S}(\psi ^*_\lambda )$ is given by $\{x \in \Omega :{{\mathrm{dist}}}(x,\mathrm{supp}\,\psi _\lambda ^*) \le C 2^{-|\lambda |}\}$.

#### Proof

For $\mu ,\lambda \in \vee _*$ with $|\mu |=|\lambda |-1$ and ${{\mathrm{meas}}}(\mathrm{supp}\,\psi ^*_\lambda \cap \mathrm{supp}\,\psi ^*_\mu )>0$, and $x \in \Omega$ with ${{\mathrm{dist}}}(x,\mathrm{supp}\,\psi _\lambda ^*) \le C 2^{-|\lambda |}$, it holds that ${{\mathrm{dist}}}(x,\mathrm{supp}\,\psi _\mu ^*) \le {{\mathrm{dist}}}(x,\mathrm{supp}\,\psi _\lambda ^*)+{{\mathrm{diam}}}(\mathrm{supp}\,\psi ^*_\lambda ) \le C2^{-|\mu |}$. $\square$

A proof of the following proposition, as well as an algorithm to apply the multi-to-single-scale transformation that is mentioned, is given in [31, §4.3].

#### Proposition 5.6

Given a tree $\Lambda \subset \vee _*$, there exists a tiling ${\mathcal {T}}({\Lambda }) \subset \mathcal {O}_\Omega$ with $\# {\mathcal {T}}({\Lambda }) \lesssim \# \Lambda$ such that ${{\mathrm{span}}}\{ \psi ^*_\lambda :\lambda \in \Lambda \}\subset \mathcal {P}_{m}({\mathcal {T}}({\Lambda }))$. Moreover, equipping $\mathcal {P}_{m}(\mathcal {T}(\Lambda ))$ with a basis of functions, each of which supported in $\omega$ for one $\omega \in \mathcal {T}(\Lambda )$, the representation of this embedding, known as the multi- to single-scale transform, can be applied in ${\mathcal {O}}(\#\Lambda )$ operations.

The benefit of the definition of $\mathcal {S}(\psi _\lambda ^*)$ appears from the following lemma.

#### Lemma 5.7

Let $$\overline{\Omega }=\Sigma _0 \supseteq \Sigma _1 \supseteq \cdots $$. Then

$$\begin{aligned} \Lambda ^*:=\big \{\lambda \in \vee _* :{{\mathrm{meas}}}(\mathcal {S}(\psi _\lambda ^*) \cap \Sigma _{|\lambda |})>0\big \} \end{aligned}$$

is a tree.

#### Proof

The set $\Lambda ^*$ contains all $\lambda \in \vee _*$ with $|\lambda |=0$, as well as, by definition of $\mathcal {S}(\cdot )$, the parent of any $\lambda \in \Lambda ^*$ with $|\lambda |>0$. $\square$

In Proposition 5.6 we saw that for each tree $\Lambda$ there exists a tiling ${\mathcal {T}}(\Lambda )$, with $\# {\mathcal {T}}(\Lambda ) \lesssim \#\Lambda$, such that ${{\mathrm{span}}}\{ \psi ^*_\lambda :\lambda \in \Lambda \}\subset \mathcal {P}_{m}({\mathcal {T}}({\Lambda }))$. Conversely, in the following, given a tiling ${\mathcal {T}}$, and a constant $k \in \mathbb {N}_0$, we construct a tree $\Lambda ^*({{\mathcal {T}},k})$ with $\# \Lambda ^*({{\mathcal {T}},k}) \lesssim \# {\mathcal {T}}$ (dependent on *k*) such that a kind of reversed statements hold: In “Appendix A”, statements of type $\lim _{k \rightarrow \infty } \sup _{0 \ne g \in \mathcal {P}_{m}({\mathcal {T}})} \frac{\Vert \langle \Psi ^*,g\rangle _{L_2(\Omega )}|_{\vee _*{\setminus } \Lambda ^*({{\mathcal {T}},k})}\Vert }{\Vert g\Vert _{*'}} = 0$ will be shown, meaning that for any tolerance there exist a *k* such that for any $g \in \mathcal {P}_{m}({\mathcal {T}})$ the relative error in dual norm in the best approximation from the span of the corresponding dual wavelets with indices in $\Lambda ^*({{\mathcal {T}},k})$ is less than this tolerance.

#### Definition 5.8

Given a tiling ${\mathcal {T}} \subset \mathcal {O}_\Omega$, let $t({\mathcal {T}}) \subset \mathcal {O}_\Omega$ be its enlargement by adding all ancestors of all $\omega \in {\mathcal {T}}$. Given a $k \in \mathbb {N}_0$, we set

$$\begin{aligned} \Lambda ^*({{\mathcal {T}},k}):=\bigg \{\lambda \in \vee _* :{{\mathrm{meas}}}\left( \mathcal {S}(\psi _\lambda ^*) \cap \bigcup _{\{\omega \in t({\mathcal {T}}):|\omega |=\max (|\lambda |-k,0)\}} \omega \right) >0\bigg \}. \end{aligned}$$

#### Proposition 5.9

The set $\Lambda ^*({{\mathcal {T}},k})$ is a tree, and $\# \Lambda ^*({{\mathcal {T}},k}) \lesssim \# {\mathcal {T}}$ (dependent on $k \in \mathbb {N}_0$).

#### Proof

The first statement follows from Lemma 5.7. Since the number of children of any $\omega \in \mathcal {O}_\Omega$ is uniformly bounded, it holds that $\# t({\mathcal {T}})\lesssim \# {\mathcal {T}}$, and so $\# \Lambda ^*({{\mathcal {T}},k}) \lesssim \# {\mathcal {T}}$ as a consequence of the wavelets being locally supported. $\square$

#### Example 5.10

Let $\Psi =\{\psi _\lambda :\lambda \in \vee \}$ be the collection of Haar wavelets on $\Omega =(0,1)$, i.e., the union of the function $\psi _{0,0} \equiv 1$, and, for $\ell \in \mathbb {N}$ and $k=0,\ldots ,2^{\ell -1}-1$, the functions $\psi _{\ell ,k}:=2^{\frac{\ell -1}{2}}\psi (2^{\ell -1}\cdot -k)$, where $\psi \equiv 1$ on $[0,\frac{1}{2}]$ and $\psi \equiv -1$ on $(\frac{1}{2},1]$. Writing $\lambda =(\ell ,k)$, we set $|\lambda |=\ell$. The parent of $\lambda$ with $|\lambda |>0$ is $\mu$ with $|\mu |=|\lambda |-1$ and $\mathrm{supp}\,\psi _\lambda \subset \mathrm{supp}\,\psi _\mu$, and $\mathcal {S}(\psi _\lambda ):=\mathrm{supp}\,\psi _\lambda$.

Let $\mathcal {O}_{\Omega }$ be the union, for $\ell \in \mathbb {N}_0$ and $k=0,\ldots ,2^\ell -1$, of the intervals $2^{\ell }[k,k+1]$ to which we assign the level $\ell$.

**Fig. 1:**
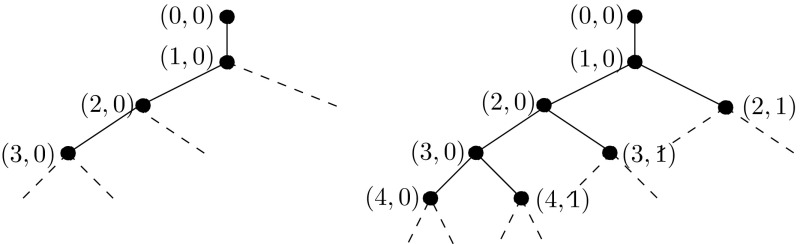
The index set of the Haar basis given as an infinite binary tree, and its subsets $\Lambda =\{(0,0),(1,0),(2,0),(3,0)\}$ (left) and $\Lambda (\mathcal {T}(\Lambda ),1)$ (right)

Now as an example let $\Lambda \subset \vee$ be the set $\{(0,0),(1,0),(2,0),(3,0)\}$, which is a tree in the sense of Definition 5.4. It corresponds to the solid parts in the left picture in Fig. 1.

The (minimal) tiling ${\mathcal {T}}(\Lambda )$ as defined in Proposition 5.6 is given by $\{[0,\frac{1}{8}],[\frac{1}{8},\frac{1}{4}], [\frac{1}{4},\frac{1}{2}], [\frac{1}{2},1]\}$.

Conversely, taking ${\mathcal {T}}:={\mathcal {T}}(\Lambda )$, the set $\Lambda (\mathcal {T},1) \subset \vee$ as defined in Definition 5.8 is given by $\{(0,0),(1,0),(2,0),(2,1),(3,0),(3,1), (4,0), (4,1)\}$ and is illustrated in the right picture in Fig. 1.

#### Definition 5.11

Recalling from (4.1) and the first lines of this subsection that the Riesz basis $\Psi$ for $\mathscr {U}\times \mathscr {T}$ is of canonical form

$$\begin{aligned} \Psi= & {} (\Psi ^{\mathscr {U}},0_{\mathscr {T}_1},\ldots ,0_{\mathscr {T}_n}) \cup (0_\mathscr {U},\Psi ^{\mathscr {T}_1},0_{\mathscr {T}_2},\ldots ,0_{\mathscr {T}_n}) \cup \cdots \\&\cup (0_\mathscr {U},0_{\mathscr {T}_1},\ldots ,0_{\mathscr {T}_{n-1}},\Psi ^{\mathscr {T}_n}), \end{aligned}$$

with index set $\vee :=\vee _{\mathscr {U}} \cup \vee _{\mathscr {T}_1} \cup \cdots \cup \vee _{\mathscr {T}_n}$, we call $\Lambda \subset \vee$ *admissible* when each of $\Lambda \cap \vee _{\mathscr {U}},\Lambda \cap \vee _{\mathscr {T}_1},\,\ldots ,\Lambda \cap \vee _{\mathscr {T}_n}$ are trees. The tiling ${\mathcal {T}}(\Lambda )$ is defined as the smallest common refinement ${\mathcal {T}}(\Lambda \cap \vee _{\mathscr {U}}) \oplus {\mathcal {T}}(\Lambda \cap \vee _{\mathscr {T}_1})\oplus \cdots \oplus {\mathcal {T}}(\Lambda \cap \vee _{\mathscr {T}_n})$. Conversely, given a tiling ${\mathcal {T}} \subset \mathcal {O}_\Omega$ and a $k \in \mathbb {N}_0$, we define the admissible set $\Lambda ({\mathcal {T}},k) \subset \vee$ by $\Lambda ^\mathscr {U}({\mathcal {T}},k)\cup \Lambda ^{\mathscr {T}_1}({\mathcal {T}},k) \cup \cdots \cup \Lambda ^{\mathscr {T}_n}({\mathcal {T}},k)$.

#### Remark 5.12

Let $\Psi ^{\mathscr {U}}$ be a wavelet basis for $\mathscr {U}$ of order $d_\mathscr {U}>1$ (i.e., all wavelets $\psi _\lambda ^{\mathscr {U}}$ up to level $\ell$ span all piecewise polynomials in $\mathscr {U}$ of degree $d_\mathscr {U}-1$ w.r.t. $\{\omega :\omega \in \mathcal {O}_\Omega ,\,|\omega |=\ell \}$), and similarly, for $1 \le q \le n$, let $\Psi ^{\mathscr {T}_q}$ be a wavelet basis for $\mathscr {T}_q$ of order $d_\mathscr {T}>0$. Recalling the definition of an approximation class given in (3.1), a sufficiently smooth solution $(u,\vec {\theta })$ is in $\mathcal {A}^s$ for $s=s_{\max }:=\min (\frac{d_\mathscr {U}-1}{n},\frac{d_\mathscr {T}}{n})$, whereas on the other hand membership of $\mathcal {A}^s$ for $s>s_{\max }$ cannot be expected under whatever smoothness condition.

For $s \le s_{\max }$, a sufficient and ‘nearly’ necessary condition for $(u,\vec {\theta })\in \mathcal {A}^s$ is that $(u,\vec {\theta }) \in B^{s n+1}_{p,\tau }(\Omega ) \times B^{s n}_{p,\tau }(\Omega )^n$ for $\frac{1}{p}<s+\frac{1}{2}$ and arbitrary $\tau >0$, see [11]. This mild smoothness condition in the ‘Besov scale’ has to be compared to the condition $(u,\vec {\theta }) \in H^{s n+1}(\Omega ) \times H^{s n}(\Omega )^n$ that is necessary to obtain a rate *s* with approximation from the spaces of type ${{\mathrm{span}}}\{\psi ^\mathscr {U}_\lambda :|\lambda |\le L\} \times \prod _{q=1}^n {{\mathrm{span}}}\{\psi ^{\mathscr {T}_q}_\lambda :|\lambda |\le L\}$.

We pause to add *three more assumptions* on our PDE: We assume that w.r.t. the coarsest possible tiling $\{\omega :\omega \in \mathcal {O}_\Omega ,\,|\omega |=0\}$ of $\bar{\Omega }$,

5.5 $$\begin{aligned}&A \text { is piecewise polynomial,} \end{aligned}$$

5.6 $$\begin{aligned}&N(u) \text { is a partial differential operator of at most first order, with coefficients that}\nonumber \\&\quad \text {are piecewise polynomials in } u \text { and } x, \text { and}\end{aligned}$$

5.7 $$\begin{aligned}&\overline{\Gamma }_{N}, \text { and so } \overline{\Gamma }_D, \text { is the union of facets of } \omega \in \mathcal {O}_\Omega \text { with } |\omega |=0. \end{aligned}$$

#### Remark 5.13

The subsequent analysis can easily be generalized to *A* being piecewise smooth. With some more efforts other nonlinear terms *N* can be handled as well. For example, for *N*(*u*) of the form $n_1(u) u$, it will be needed that for some $m \in \mathbb {N}$, for each $\omega \in \mathcal {O}_\Omega$, there exists a subspace $V_\omega \subset H^1_0(\omega )$ with $\Vert \cdot \Vert _{H^1(\omega )} \lesssim 2^{|\omega |} \Vert \cdot \Vert _{L_2(\omega )}$ on $V_\omega$, and

$$\begin{aligned} \Vert N(p_1) +p_2\Vert _{L_2(\omega )} \lesssim \sup _{0 \ne v \in V_\omega } \frac{\langle N(p_1) +p_2,v\rangle _{L_2(\omega )}}{\Vert v\Vert _{L_2(\omega )}} \quad (p_1, p_2 \in \mathcal {P}_m(\omega )) \end{aligned}$$

(cf. proof of Lemma A.2).

Finally in this subsection we formulate our assumptions on the wavelet Riesz basis $\Psi ^{\mathscr {V}_2}=\{\psi ^{\mathscr {V}_2}_\lambda :\lambda \in \vee _{\mathscr {V}_2}\}$ for $H^{-\frac{1}{2}}(\Gamma _D)$. We assume that it satisfies the assumptions ($w_{2}$) and ($w_{3}$) with $\mathcal {O}_\Omega$ reading as

$$\begin{aligned} \mathcal {O}_{\Gamma _D}=\{\omega \cap \Gamma _D:\omega \in \mathcal {O}_\Omega ,\,{{\mathrm{meas}}}_{n-1}(\omega \cap \Gamma _D)>0\}. \end{aligned}$$

Furthermore, we impose that

5.8 $$\begin{aligned} \Vert \psi ^{\mathscr {V}_2}_\lambda \Vert _{H^{-1}(\Gamma _D)} \lesssim 2^{-|\lambda |/2}. \end{aligned}$$

which, for biorthogonal wavelets, is essentially is ($w_{4}$) (cf. [20, lines following (A.2)]). (To relax the smoothness conditions on $\Gamma _D$ needed for the definition of $H^{-1}(\Gamma _D)$, one can replace (5.8) by $\Vert \psi ^{\mathscr {V}_2}_\lambda \Vert _{H^s(\Gamma _D)} \lesssim 2^{|\lambda |(s+\frac{1}{2})}$ for some $s \in [-1,-\frac{1}{2})$).

The definition of a boundary tiling ${\mathcal {T}}_{\Gamma _D} \subset \mathcal {O}_{\Gamma _D}$ is similar to Definition 5.3. Also similar to the corresponding preceding definitions are that of a tree $\Lambda \subset \vee _{\mathscr {V}_2}$, and of the boundary tiling ${\mathcal {T}}_{\Gamma _D}(\Lambda ) \subset \mathcal {O}_{\Gamma _D}$ for $\Lambda \subset \vee _{\mathscr {V}_2}$ being a tree. Conversely, for a boundary tiling ${\mathcal {T}}_{\Gamma _D}\subset \mathcal {O}_{\Gamma _D}$ and $k \in \mathbb {N}_0$, for $*\in \{\mathscr {U},\mathscr {V}_2\}$ we define the tree

$$\begin{aligned} \Lambda ^*({{\mathcal {T}}_{\Gamma _D},k}):= \bigg \{\lambda \in \vee _*:{{\mathrm{meas}}}_{n-1}\left( \mathcal {S}(\psi _\lambda ^*) \cap \bigcup _{\{\omega \in \bar{\mathcal {T}}_{\Gamma _D}:|\omega |= \max (|\lambda |-k,0)\}} \omega \right) >0\bigg \}. \end{aligned}$$

### An appropriate approximate residual evaluation

Given an admissible $\Lambda \subset \vee$, $[{\tilde{\mathbf{u}}}^\top ,\varvec{\tilde{\theta }}^\top ]^\top \in \ell _2(\Lambda )$ with $(\tilde{u},\vec {\tilde{\theta }}):=[{\tilde{\mathbf{u}}}^\top ,\varvec{\tilde{\theta }}^\top ] \Psi$ sufficiently close to $(u,\vec {\theta })$, and an $\varepsilon >0$, our approximate evaluation of $D\mathbf{Q}([{\tilde{\mathbf{u}}}^\top ,\varvec{\tilde{\theta }}^\top ]^\top )$, given in (5.4), is built in the following steps, where $k \in \mathbb {N}_0$ is a sufficiently large constant:

1. Find a tiling ${\mathcal {T}}(\varepsilon ) \subset \mathcal {O}_\Omega$, such that
  $$\begin{aligned}&\inf _{(g_\varepsilon ,f_\varepsilon ,\vec {h}_\varepsilon ) \in \mathcal {P}_m({\mathcal {T}}(\varepsilon )\cap \Gamma _D) \cap C(\Gamma _D) \times \mathcal {P}_m({\mathcal {T}}(\varepsilon )) \times \mathcal {P}_m({\mathcal {T}}(\varepsilon ))^n} \left( \Vert g-g_\varepsilon \Vert _{H^{\frac{1}{2}}(\Gamma _D)} \right. \\&\left. \quad +\,\Vert v_1 \mapsto \int _\Omega (f-f_\varepsilon ) v_1\,dx+\int _{\Gamma _N}(h-\vec {h}_\varepsilon \cdot \mathbf{n}) v_1 \,ds\Vert _{\mathscr {V}_1'}\right) \le \varepsilon . \end{aligned}$$
  Set $${\mathcal {T}}(\Lambda ,\varepsilon ):={\mathcal {T}}(\Lambda ) \oplus {\mathcal {T}}(\varepsilon )$$.
2.
  - Approximate
    $$\begin{aligned} \mathbf{r}^{(\frac{1}{2})}_1:=\langle \Psi ^{\mathscr {V}_1}, N(\tilde{u})-f- {{\mathrm{div}}}\vec {\tilde{\theta }}\rangle _{L_2(\Omega )}+\langle \Psi ^{\mathscr {V}_1}, \vec {\tilde{\theta }}\cdot \mathbf{n}-h\rangle _{L_2(\Gamma _N)} \end{aligned}$$
    by
    $$\begin{aligned} {\tilde{\mathbf{r}}}^{\left( \frac{1}{2}\right) }_1:=\mathbf{r}^{\left( \frac{1}{2}\right) }_1|_{\Lambda ^{\mathscr {V}_1}({\mathcal {T}}(\Lambda ,\varepsilon ),k)}. \end{aligned}$$
  - With $$\tilde{r}_1^{(\frac{1}{2})}:=({\tilde{\mathbf{r}}}^{(\frac{1}{2})}_1)^\top \Psi ^{\mathscr {V}_1}$$, approximate
    $$\begin{aligned} \mathbf{r}_1=\left[ \begin{array}{@{}l@{}} \mathbf{r}_{11} \\ \mathbf{r}_{12}\end{array} \right] :=\left[ \begin{array}{@{}l@{}} \langle DN(\tilde{u}) \Psi ^{\mathscr {U}}, \tilde{r}_1^{(\frac{1}{2})} \rangle _{L_2(\Omega )}\\ \langle \Psi ^{\mathscr {T}},\nabla \tilde{r}_1^{(\frac{1}{2})} \rangle _{L_2(\Omega )^n} \end{array} \right] \end{aligned}$$
    by $${\tilde{\mathbf{r}}}_1= \left[ \begin{array}{@{}l@{}} {\tilde{\mathbf{r}}}_{11} \\ {\tilde{\mathbf{r}}}_{12}\end{array} \right] := \mathbf{r}_1|_{\Lambda ({\mathcal {T}}(\Lambda ^{\mathscr {V}_1}({\mathcal {T}}(\Lambda ,\varepsilon ),k)),k)}$$.
3. Approximate
  $$\begin{aligned} \mathbf{r}_2:=\left\langle \left[ \begin{array}{@{}l@{}} -\,A \nabla \Psi ^{\mathscr {U}}\\ \Psi ^{\mathscr {T}} \end{array} \right] ,\vec {\tilde{\theta }}-A \nabla \tilde{u} \right\rangle _{L_2(\Omega )^n} \end{aligned}$$
  by $${\tilde{\mathbf{r}}}_2:=\mathbf{r}_2|_{\Lambda ({\mathcal {T}}(\Lambda ),k)}$$.
4.
  - Approximate $$\mathbf{r}^{(\frac{1}{2})}_3:=\langle \Psi ^{\mathscr {V}_2}, \tilde{u}-g\rangle _{L_2(\Gamma _D)}$$ by
    $$\begin{aligned} {\tilde{\mathbf{r}}}^{\left( \frac{1}{2}\right) }_3:=\mathbf{r}^{\left( \frac{1}{2}\right) }_3|_{\Lambda ^{\mathscr {V}_2}({\mathcal {T}}(\Lambda ,\varepsilon )\cap \Gamma _D,k)}. \end{aligned}$$
  - With $\tilde{r}_3^{\left( \frac{1}{2}\right) }:=({\tilde{\mathbf{r}}}^{(\frac{1}{2})}_3)^\top \Psi ^{\mathscr {V}_2}$, approximate $$\mathbf{r}_3:=\left[ \begin{array}{@{}l@{}} \langle \Psi ^\mathscr {U},\tilde{r}_3^{(\frac{1}{2})}\rangle _{L_2(\Gamma _D)}\\ 0_{\vee _\mathscr {T}} \end{array}\right] $$ by
    $$\begin{aligned} {\tilde{\mathbf{r}}}_3:=\mathbf{r}_3|_{\Lambda ({\mathcal {T}}_{\Gamma _D}(\Lambda ^{\mathscr {V}_2}({\mathcal {T}}(\Lambda ,\varepsilon )\cap \Gamma _D,k)),k)}. \end{aligned}$$

Although (s2)–(s4) may look involved at first glance, the same kind of approximation is used at all instances. Each term in (5.4) consists essentially of a wavelet basis that is integrated against a piecewise polynomial, or more precisely, a function that can be sufficiently accurately approximated by a piecewise polynomial thanks to the control of the data oscillation by the refinement of the partition performed in (s1). In each of these terms, all wavelets are neglected whose levels exceed locally the level of the partition plus a constant *k*.

In the next theorem it is shown that this approximate residual evaluation satisfies the condition for optimality of the adaptive wavelet Galerkin method.

#### Theorem 5.14

For an admissible $\Lambda \subset \vee$, $[{\tilde{\mathbf{u}}}^\top ,\varvec{\tilde{\theta }}^\top ]^\top \in \ell _2(\Lambda )$ with $(\tilde{u},\vec {\tilde{\theta }}):=[{\tilde{\mathbf{u}}}^\top ,\varvec{\tilde{\theta }}^\top ] \Psi$ sufficiently close to $(u,\vec {\theta })$, and an $\varepsilon >0$, consider the steps (s1)–(s4). With $s>0$ such that $[\mathbf{u}^\top ,\varvec{\theta }^\top ]^\top \in \mathcal {A}^s$, let ${\mathcal {T}}(\varepsilon )$ from (s1) satisfy $\# {\mathcal {T}}(\varepsilon ) \lesssim \varepsilon ^{-1/s}$. Then

$$\begin{aligned} \Vert D\mathbf{Q}([{\tilde{\mathbf{u}}}^\top ,\varvec{\tilde{\theta }}^\top ]^\top )-({\tilde{\mathbf{r}}}_1+{\tilde{\mathbf{r}}}_2+{\tilde{\mathbf{r}}}_3)\Vert \lesssim 2^{-k/2} (\Vert u-\tilde{u}\Vert _\mathscr {U}+\Vert \vec {\theta }-\vec {\tilde{\theta }}\Vert _\mathscr {T})+\varepsilon , \end{aligned}$$

where the computation of ${\tilde{\mathbf{r}}}_1+{\tilde{\mathbf{r}}}_2+{\tilde{\mathbf{r}}}_3$ requires ${\mathcal {O}}(\# \Lambda +\varepsilon ^{-1/s})$ operations. So by taking *k* sufficiently large, Condition 3.5* is satisfied.

Before giving the proof of this theorem, let us discuss matters related to step (s1). First of all, *existence* of tilings ${\mathcal {T}}(\varepsilon )$ as mentioned in the theorem is guaranteed. Indeed, because of $[\mathbf{u}^\top ,\varvec{\theta }^\top ]^\top \in \mathcal {A}^s$, given $C>0$, for any $\varepsilon >0$ there exists a $[{\tilde{\mathbf{u}}}_\varepsilon ^\top ,\varvec{\tilde{\theta }}_\varepsilon ^\top ]^\top \in \ell _2(\Lambda _\varepsilon )$, where $\Lambda _\varepsilon$ is admissible and $\#\Lambda _\varepsilon \lesssim \varepsilon ^{-1/s}$, such that $\Vert [\mathbf{u}^\top ,\varvec{\theta }^\top ]^\top -[{\tilde{\mathbf{u}}}_\varepsilon ^\top ,\varvec{\tilde{\theta }}_\varepsilon ^\top ]^\top \Vert \le \varepsilon /C$. By taking a suitable *C*, from Lemma 2.6, (5.2) and (5.3) we infer that with $(\tilde{u}_\varepsilon ,\vec {\tilde{\theta }}_\varepsilon ):=[{\tilde{\mathbf{u}}}_\varepsilon ^\top ,\varvec{\tilde{\theta }}_\varepsilon ^\top ] \Psi$,

$$\begin{aligned}&\Vert \tilde{u}_\varepsilon -g\Vert _{H^{\frac{1}{2}}(\Gamma _D)}+\Vert v_1\mapsto \int _\Omega (N(\tilde{u}_\varepsilon )-{{\mathrm{div}}}\vec {\tilde{\theta }}_\varepsilon -f)v_1\,dx\\&\quad +\,\int _{\Gamma _N} (\vec {\tilde{\theta }}_\varepsilon \cdot \mathbf{n}-h) v_1 \,ds\Vert _{\mathscr {V}_1'} \le \varepsilon . \end{aligned}$$

Since $\tilde{u}_\varepsilon \in \mathcal {P}_m(\mathcal {T}(\Lambda _\varepsilon )) \cap C(\Omega )$, $N(\tilde{u}_\varepsilon )-{{\mathrm{div}}}\vec {\tilde{\theta }}_\varepsilon \in \mathcal {P}_m(\mathcal {T}(\Lambda _\varepsilon ))$, $\vec {\tilde{\theta }}_\varepsilon \in \mathcal {P}_m(\mathcal {T}(\Lambda _\varepsilon ))^n$, and $\# \mathcal {T}(\Lambda _\varepsilon ) \lesssim \# \Lambda _\varepsilon$, the tiling ${\mathcal {T}}(\varepsilon ):= \mathcal {T}(\Lambda _\varepsilon )$ satisfies the assumptions.

Loosely speaking, this result can be rephrased by saying that if the solution of $D\mathbf{Q}([\mathbf{u}^\top ,\varvec{\theta }^\top ]^\top )=0$ is in $\mathcal {A}^s$, then so is the forcing function (*f*, *g*, *h*). This is not automatically true, cf. [12] for a discussion in the adaptive finite element context, but in the current setting it is a consequence of the fact that, thanks to assumption (5.3), the first order partial differential operators apply to the wavelet bases $\Psi ^*$ for $*\in \{\mathscr {U},\mathscr {T}_1,\ldots ,\mathscr {T}_n,\mathscr {V}_1,\mathscr {V}_2\}$ in ‘mild’ sense (the result of the application of each of these operators lands in $L_2$-space).

Knowing that a suitable $\mathcal {T}(\varepsilon )$ exists is different from knowing how to construct it. For our convenience thinking of $g=h=0$, and so $\mathscr {U}=\mathscr {V}_1=H^1_0(\Omega )$, assuming that $f \in L_2(\Omega )$ one has $\inf _{f_\varepsilon \in \mathcal {P}_m(\mathcal {T})} \Vert f-f_\varepsilon \Vert ^2_{H^{-1}(\Omega )} \lesssim \mathrm {osc}(f,\mathcal {T})^2:=\sum _{\omega \in \mathcal {T}} {{\mathrm{diam}}}(\omega )^2 \inf _{f_\omega \in \mathcal {P}_m(\omega )} \Vert f-f_\omega \Vert ^2_{L_2(\omega )}$. Ignoring quadrature issues, for any partition $\mathcal {T}$, $\mathrm {osc}(f,\mathcal {T})$ is computable. A quasi-minimal partition $\mathcal {T}(\varepsilon )$ such that $\mathrm {osc}(f,\mathcal {T}(\varepsilon )) \lesssim \varepsilon$ can be computed using the Thresholding Second Algorithm from [2]. Now the assumption to be added to Theorem 5.14 is that for such a partition, $\# \mathcal {T}(\varepsilon ) \lesssim \varepsilon ^{-1/s}$.

Note that it is nowhere needed to explicitly approximate the forcing functions by approximating their wavelet expansions.

#### Proof of Theorem 5.14

By construction we have

$$\begin{aligned}&D\mathbf{Q}([{\tilde{\mathbf{u}}}^\top ,\varvec{\tilde{\theta }}^\top ]^\top )-({\tilde{\mathbf{r}}}_1+{\tilde{\mathbf{r}}}_2+{\tilde{\mathbf{r}}}_3) =\left[ \begin{array}{@{}l@{}} \langle DN(\tilde{u}) \Psi ^{\mathscr {U}}, \Psi ^{\mathscr {V}_1} \rangle _{L_2(\Omega )}\\ \langle \Psi ^{\mathscr {T}},\nabla \Psi ^{\mathscr {V}_1} \rangle _{L_2(\Omega )^n} \end{array} \right] \left( \mathbf{r}^{\left( \frac{1}{2}\right) }_1-{\tilde{\mathbf{r}}}^{\left( \frac{1}{2}\right) }_1\right) \\&\quad +\,\mathbf{r}_1-{\tilde{\mathbf{r}}}_1 + \mathbf{r}_2-{\tilde{\mathbf{r}}}_2+ \left[ \begin{array}{@{}l@{}} \langle \Psi ^\mathscr {U},\Psi ^{\mathscr {V}_2}\rangle _{L_2(\Gamma _D)}\\ 0_{\vee _\mathscr {T}} \end{array}\right] \left( \mathbf{r}^{\left( \frac{1}{2}\right) }_3-{\tilde{\mathbf{r}}}_3^{\left( \frac{1}{2}\right) }\right) +\mathbf{r}_3-{\tilde{\mathbf{r}}}_3. \end{aligned}$$

From $\Psi ^{\mathscr {U}}$, $\Psi ^{\mathscr {T}}$, and $\Psi ^{\mathscr {V}_1}$ being Riesz bases, and $N:\mathscr {U}\supset \mathrm {dom}(N) \rightarrow \mathscr {V}_1'$ being continuously differentiable at *u*, one infers that $$ \left[ \begin{array}{@{}l@{}} \langle DN(\tilde{u}) \Psi ^{\mathscr {U}}, \Psi ^{\mathscr {V}_1} \rangle _{L_2(\Omega )}\\ \langle \Psi ^{\mathscr {T}},\nabla \Psi ^{\mathscr {V}_1} \rangle _{L_2(\Omega )^n} \end{array} \right] \in \mathcal {L}(\ell _2(\vee _{\mathscr {V}_1}),\ell _2(\vee ))$$, with a norm that is bounded uniformly in $\tilde{u}$ from a neighbourhood of *u*. Similarly, from $u \mapsto u|_{\Gamma _D} \in \mathcal {L}(\mathscr {U},H^{\frac{1}{2}}(\Gamma _D))$, and $\Psi ^\mathscr {U}$ and $\Psi ^{\mathscr {V}_2}$ being Riesz bases for $\mathscr {U}$ and $H^{\frac{1}{2}}(\Gamma _D)'$, respectively, we infer that $$\left[ \begin{array}{@{}l@{}} \langle \Psi ^\mathscr {U},\Psi ^{\mathscr {V}_2}\rangle _{L_2(\Gamma _D)}\\ 0 \end{array}\right] \in \mathcal {L}(\ell _2(\vee _{\mathscr {V}_2}),\ell _2(\vee ))$$. We conclude that

5.9 $$\begin{aligned} \begin{aligned}&\Vert D\mathbf{Q}([{\tilde{\mathbf{u}}}^\top ,\varvec{\tilde{\theta }}^\top ]^\top )-({\tilde{\mathbf{r}}}_1+{\tilde{\mathbf{r}}}_2+{\tilde{\mathbf{r}}}_3)\Vert \\&\quad \lesssim \Vert \mathbf{r}^{\left( \frac{1}{2}\right) }_1-{\tilde{\mathbf{r}}}^{\left( \frac{1}{2}\right) }_1\Vert + \Vert \mathbf{r}_1-{\tilde{\mathbf{r}}}_1\Vert + \Vert \mathbf{r}_2-{\tilde{\mathbf{r}}}_2\Vert +\Vert \mathbf{r}^{\left( \frac{1}{2}\right) }_3-{\tilde{\mathbf{r}}}^{\left( \frac{1}{2}\right) }_3\Vert + \Vert \mathbf{r}_3-{\tilde{\mathbf{r}}}_3\Vert . \end{aligned} \end{aligned}$$

We bound all terms at the right-hand side.

With $f_\varepsilon$, $\vec {h}_\varepsilon$ from (s1), using that $\Psi ^{\mathscr {V}_1}$ is a Riesz basis, we have that

5.10 $$\begin{aligned}&\Vert \mathbf{r}^{\big (\frac{1}{2}\big )}_1-{\tilde{\mathbf{r}}}^{\big (\frac{1}{2}\big )}_1\Vert \lesssim \varepsilon \nonumber \\&\quad +\Big \Vert \Big (\langle \Psi ^{\mathscr {V}_1}, N(\tilde{u})-f_\varepsilon - {{\mathrm{div}}}\vec {\tilde{\theta }}\rangle _{L_2(\Omega )}+\langle \Psi ^{\mathscr {V}_1}, (\vec {\tilde{\theta }}-\vec {h}_\varepsilon )\cdot \mathbf{n}\rangle _{L_2(\Gamma _N)}\Big )\big |_{\vee _{\mathscr {V}_1}{\setminus } \Lambda ^{\mathscr {V}_1}({\mathcal {T}}(\Lambda ,\varepsilon ),k)} \Big \Vert . \end{aligned}$$

From $N(\tilde{u})-f_\varepsilon - {{\mathrm{div}}}\vec {\tilde{\theta }} \in \mathcal {P}_m({\mathcal {T}}(\Lambda ,\varepsilon ))$, and $\vec {\tilde{\theta }}-\vec {h}_\varepsilon \in \mathcal {P}_m({\mathcal {T}}(\Lambda ,\varepsilon ))^n$, Proposition A.1 shows that the norm at the right-hand side of (5.10) is $\lesssim 2^{-k} \big \Vert v_1 \mapsto \int _\Omega \vec {\tilde{\theta }} \cdot \nabla v_1+(N(\tilde{u})-f_\varepsilon )v_1 \,dx -\int _{\Gamma _N} \vec {h}_\varepsilon \cdot \mathbf{n} v_1 \,ds \big \Vert _{\mathscr {V}_1'}$. Again by using (s1), we infer that

5.11 $$\begin{aligned} \Vert \mathbf{r}^{\big (\frac{1}{2}\big )}_1-{\tilde{\mathbf{r}}}^{\big (\frac{1}{2}\big )}_1\Vert \lesssim \varepsilon + 2^{-k} \big \Vert v_1 \mapsto \int _\Omega \vec {\tilde{\theta }} \cdot \nabla v_1+(N(\tilde{u})-f)v_1 \,dx -\int _{\Gamma _N} h v_1 \,ds \big \Vert _{\mathscr {V}_1'}.\nonumber \\ \end{aligned}$$

Thanks to $\nabla \tilde{r}_1^{(\frac{1}{2})} \in \mathcal {P}_m({\mathcal {T}}(\Lambda ^{\mathscr {V}_1}({\mathcal {T}}(\Lambda ,\varepsilon ),k)))^n$, an application of Proposition A.4 (first estimate) shows that

$$\begin{aligned} \Vert \mathbf{r}_{12}-{\tilde{\mathbf{r}}}_{12}\Vert&\lesssim 2^{-k/2}\Vert \tilde{r}_1^{\big (\frac{1}{2}\big )}\Vert _{\mathscr {V}_1} \eqsim 2^{-k/2} \Vert {\tilde{\mathbf{r}}}^{\big (\frac{1}{2}\big )}_{1}\Vert . \end{aligned}$$

Our assumptions on *N* show that $DN(\tilde{u})w$ is of the form $p_1(\tilde{u})w+\vec {p}_2(\tilde{u}) \cdot \nabla w$ for some piecewise polynomials $p_1$ and $p_2$ in $\tilde{u}$ and *x* w.r.t. $\{\omega :\omega \in \mathcal {O}_\Omega ,|\omega |=0\}$, where moreover $w \mapsto p_1(\tilde{u})w \in \mathcal {L}(\mathscr {U},\mathscr {V}_1')$, $\vec {w} \mapsto \vec {p}_2(\tilde{u}) \cdot \vec {w} \in \mathcal {L}(L_2(\Omega )^n,\mathscr {V}_1')$, uniformly in $\tilde{u}$ in a neighborhood of $u \in \mathscr {U}$. Consequently, applications of Propositions A.3–A.4 (second estimate) show that

$$\begin{aligned} \Vert \mathbf{r}_{11}-{\tilde{\mathbf{r}}}_{11}\Vert&\lesssim 2^{-k} \Vert p_1(\tilde{u}) \tilde{r}_{1}^{\big (\frac{1}{2}\big )}\Vert _{\mathscr {U}'}+2^{-k/2}\Vert \vec {p}_2(\tilde{u}) \tilde{r}_{1}^{\big (\frac{1}{2}\big )}\Vert _{L_2(\Omega )^n} \\&\lesssim 2^{-k/2} \Vert \tilde{r}_{1}^{\big (\frac{1}{2}\big )}\Vert _{\mathscr {V}_1} \eqsim 2^{-k/2} \Vert {\tilde{\mathbf{r}}}^{\big (\frac{1}{2}\big )}_1\Vert . \end{aligned}$$

Now use that $\Vert {\tilde{\mathbf{r}}}^{(\frac{1}{2})}_1\Vert \le \Vert \mathbf{r}^{(\frac{1}{2})}_1-{\tilde{\mathbf{r}}}^{(\frac{1}{2})}_1\Vert +\Vert \mathbf{r}^{(\frac{1}{2})}_1\Vert$, and $\Vert \mathbf{r}^{(\frac{1}{2})}_1\Vert \lesssim \big \Vert v_1 \mapsto \int _\Omega \vec {\tilde{\theta }} \cdot \nabla v_1+(N(\tilde{u})-f)v_1 \,dx -\int _{\Gamma _N} h v_1 \,ds \big \Vert _{\mathscr {V}_1'}$, to conclude that

5.12 $$\begin{aligned} \Vert \mathbf{r}_1-{\tilde{\mathbf{r}}}_1\Vert \lesssim 2^{-k/2}\Big (\varepsilon + \big \Vert v_1 \mapsto \int _\Omega \vec {\tilde{\theta }} \cdot \nabla v_1+(N(\tilde{u})-f)v_1 \,dx -\int _{\Gamma _N} h v_1 \,ds \big \Vert _{\mathscr {V}_1'}\Big ). \end{aligned}$$

Thanks to $\vec {\tilde{\theta }}-A \nabla \tilde{u}\in \mathcal {P}_m({\mathcal {T}}(\Lambda ))^n$ by (5.5), and $A^\top$ being piecewise polynomial, an application of Proposition A.4 shows that

5.13 $$\begin{aligned} \Vert \mathbf{r}_2-{\tilde{\mathbf{r}}}_2\Vert \lesssim 2^{-k/2} \Vert \vec {\tilde{\theta }}-A \nabla \tilde{u}\Vert _{L_2(\Omega )^n}. \end{aligned}$$

With $g_\varepsilon$ from (s1), using that $\Psi ^{\mathscr {V}_2}$ is a Riesz basis for $H^{-\frac{1}{2}}(\Gamma _D)$, we have that

5.14 $$\begin{aligned} \Vert \mathbf{r}^{\big (\frac{1}{2}\big )}_3-{\tilde{\mathbf{r}}}^{\big (\frac{1}{2}\big )}_3\Vert \lesssim \varepsilon + \Vert \langle \Psi ^{\mathscr {V}_2},\tilde{u} -g_\varepsilon \rangle _{L_2(\Gamma _D)}|_{\vee _{\mathscr {V}_2} {\setminus } \Lambda ^{\mathscr {V}_2}({\mathcal {T}}(\Lambda ,\varepsilon )\cap \Gamma _D,k)}\Vert . \end{aligned}$$

From $\tilde{u}|_{\Gamma _D}-g_\varepsilon \in \mathcal {P}_m({\mathcal {T}}(\Lambda ,\varepsilon )\cap \Gamma _D) \cap C(\Gamma _D)$, Proposition A.5 shows that the norm at the right-hand side of (5.14) is $\lesssim 2^{-k/2} \Vert \tilde{u}-g_\varepsilon \Vert _{H^{\frac{1}{2}}(\Gamma _D)}$. Again by using (s1), we infer that

5.15 $$\begin{aligned} \Vert \mathbf{r}^{\big (\frac{1}{2}\big )}_3-{\tilde{\mathbf{r}}}^{\big (\frac{1}{2}\big )}_3\Vert \lesssim \varepsilon +2^{-k/2} \Vert \tilde{u}-g\Vert _{H^{\frac{1}{2}}(\Gamma _D)}. \end{aligned}$$

Thanks to $\tilde{r}_3^{(\frac{1}{2})} \in \mathcal {P}_m( {\mathcal {T}}_{\Gamma _D}(\Lambda ^{\mathscr {V}_2}({\mathcal {T}}(\Lambda ,\varepsilon )\cap \Gamma _D,k)))$, Proposition A.6 shows that $\Vert \mathbf{r}_3-{\tilde{\mathbf{r}}}_3\Vert \lesssim 2^{-k/2}\Vert \tilde{r}_3^{(\frac{1}{2})}\Vert _{H^{-\frac{1}{2}}(\Gamma _D)} \eqsim 2^{-k/2}\Vert {\tilde{\mathbf{r}}}^{(\frac{1}{2})}_3\Vert$. Now use that $\Vert {\tilde{\mathbf{r}}}^{(\frac{1}{2})}_3\Vert \le \Vert \mathbf{r}^{(\frac{1}{2})}_3-{\tilde{\mathbf{r}}}^{(\frac{1}{2})}_3\Vert +\Vert \mathbf{r}^{(\frac{1}{2})}_3\Vert$, and $\Vert \mathbf{r}^{(\frac{1}{2})}_3\Vert \lesssim \Vert \tilde{u}-g\Vert _{H^{\frac{1}{2}}(\Gamma _D)}$ to conclude that

5.16 $$\begin{aligned} \Vert \mathbf{r}_3-{\tilde{\mathbf{r}}}_3\Vert \lesssim 2^{-k/2}\big (\varepsilon + \Vert \tilde{u}-g\Vert _{H^{\frac{1}{2}}(\Gamma _D)}\big ). \end{aligned}$$

By collecting the upper bounds (5.11)–(5.16) derived for all five terms at the right-hand side of (5.9), and by using Lemma 2.6 in combination with the least squares functional given in (5.2), the proof of the first statement is completed.

To bound the cost of the computations, we consider the computation of ${\tilde{\mathbf{r}}}^{(\frac{1}{2})}_1$. First, find a representation of $N(\tilde{u})-{{\mathrm{div}}}\vec {\tilde{\theta }}$ as an element of $\mathcal {P}_m({\mathcal {T}}(\Lambda ,\varepsilon ))$ by applying multi- to single-scale transforms. For each tile $\omega \in {\mathcal {T}}(\Lambda ^{\mathscr {V}_1}({\mathcal {T}}(\Lambda ,\varepsilon ),k))$, and for $\phi$ running over some basis of $\mathcal {P}_m(\omega )$, compute $\langle \phi , N(\tilde{u})-f-{{\mathrm{div}}}\vec {\tilde{\theta }}\rangle _{L_2(\omega )}$. From this, compute $[\langle \psi _\lambda ^{\mathscr {V}_1}, N(\tilde{u})-f-{{\mathrm{div}}}\vec {\tilde{\theta }}\rangle _{L_2(\Omega )}]_{\lambda \in \Lambda ^{\mathscr {V}_1}({\mathcal {T}}(\Lambda ,\varepsilon ),k)}$ by applying a transpose of a multi- to single-scale transform. Similar steps yield $[\langle \psi _\lambda ^{\mathscr {V}_1}, \vec {\tilde{\theta }}\cdot \mathbf{n}-h\rangle _{L_2(\Gamma _N)}]_{\lambda \in \Lambda ^{\mathscr {V}_1}({\mathcal {T}}(\Lambda ,\varepsilon ),k)}$. The total cost involved in computing ${\tilde{\mathbf{r}}}^{(\frac{1}{2})}_1$ is bounded by a multiple of $\# {\mathcal {T}}(\Lambda ,\varepsilon ) \lesssim \#\Lambda +\varepsilon ^{-1/s}$ operations.

Since fully analogous considerations apply to bounding the cost of the computations of ${\tilde{\mathbf{r}}}_1$, ${\tilde{\mathbf{r}}}_2$, ${\tilde{\mathbf{r}}}^{(\frac{1}{2})}_3$, and ${\tilde{\mathbf{r}}}_3$, the proof is completed. $\square$

## Numerical results

For $\Omega \subset \mathbb {R}^2$ being the L-shaped domain $(0,1)^2{\setminus } [\frac{1}{2},1)^2$, we consider the semi-linear boundary value problem6.1where, for simplicity, $f=1$ (to test our code we also tried some right hand sides corresponding to some fabricated polynomial solutions *u*). With $\mathscr {U}=H^1_0(\Omega )=\mathscr {V}$, $\mathscr {T}=L_2(\Omega )^2$, we applied the **awgm** (Algorithm 3.2), with $\mathbf{F}$ reading as $D\mathbf{Q}$, for the adaptive solution of $[\mathbf{u}^\top ,\varvec{{\theta }}^\top ]^\top$ from

$$\begin{aligned} \begin{aligned} D\mathbf{Q}([\mathbf{u}^\top ,\varvec{{\theta }}^\top ]^\top )=&\left[ \begin{array}{@{}l@{}} \langle \partial _1 \Psi ^{\mathscr {U}}, \partial _1 {u}-{\theta }_1 \rangle _{L_2(\Omega )}+\langle \partial _2 \Psi ^{\mathscr {U}}, \partial _2 {u}-{\theta }_2 \rangle _{L_2(\Omega )}\\ \langle \Psi ^{\mathscr {T}_1}, {\theta }_1- \partial _1 {u} \rangle _{L_2(\Omega )}\\ \langle \Psi ^{\mathscr {T}_2}, {\theta }_2 -\partial _2 {u} \rangle _{L_2(\Omega )}\\ \end{array}\right] \\&+\left[ \begin{array}{@{}l@{}} \langle \Psi ^{\mathscr {U}}, 3{{u}}^2 \Psi ^{\mathscr {V}} \rangle _{L_2(\Omega )}\\ \langle \Psi ^{\mathscr {T}_1},\partial _1 \Psi ^{\mathscr {V}} \rangle _{L_2(\Omega )}\\ \langle \Psi ^{\mathscr {T}_2},\partial _2 \Psi ^{\mathscr {V}} \rangle _{L_2(\Omega )} \end{array} \right] \big \langle \Psi ^{\mathscr {V}}, {{u}}^3-f-{{\mathrm{div}}}\vec {{\theta }} \big \rangle _{L_2(\Omega )}=\mathbf{0}, \end{aligned} \end{aligned}$$

where $$u:=\mathbf{u}^\top \Psi ^\mathscr {U}$$, $$\theta _i:=\varvec{\theta }_i^\top \Psi ^{\mathscr {T}_i}$$.

**Fig. 2:**
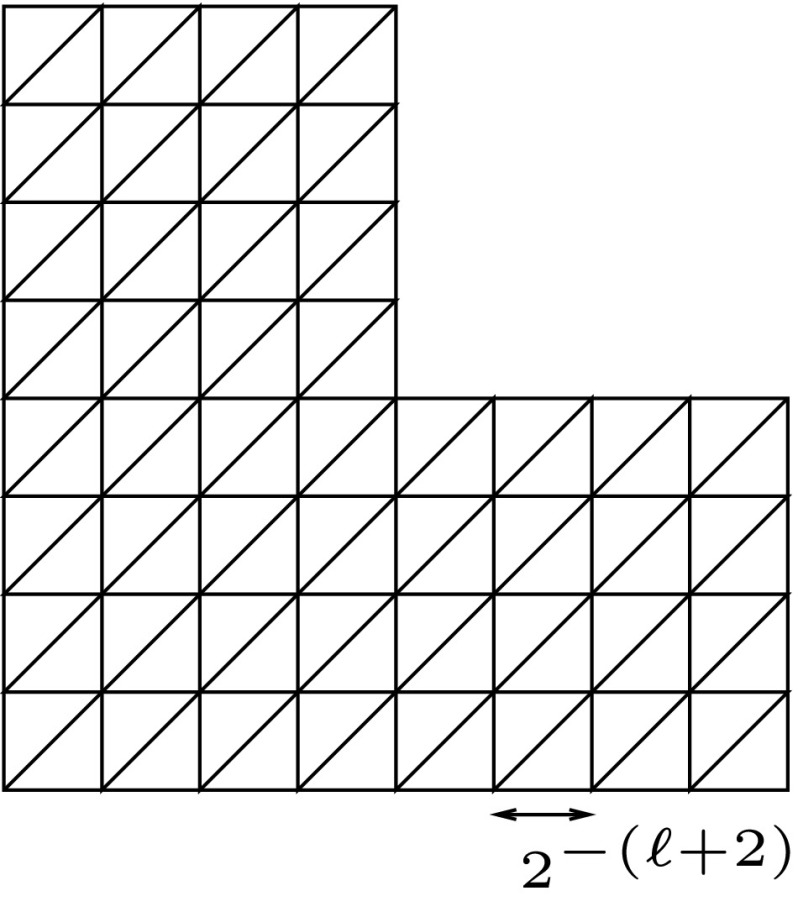
Meshes w.r.t. which the wavelets are piecewise polynomial

Here we equipped $\mathscr {T}_i$ ($i=1,2$) with the continuous piecewise linear three-point wavelet basis from [27], the space $\mathscr {V}$ with the same basis (obviously scaled differently, and with homogeneous boundary conditions incorporated), and $\mathscr {U}$ with a newly developed continuous piecewise quadratic wavelet basis. These bases can be applied on any polygon, and they satisfy all assumptions ($w_{1}$)–($w_{4}$). In particular all wavelets except possibly those ‘near’ the Dirichlet boundary have one vanishing moment. For each basis, to each wavelet that is not on the coarsest level we associate one parent on the next coarsest level according to Definition 5.4. For any $\ell \in \mathbb {N}_0$ the subsets of the bases consisting of all wavelets up to some level span exactly the space of continuous piecewise linears, continuous piecewise linears zero at $\partial \Omega$, or continuous piecewise quadratics zero at $\partial \Omega$, respectively, w.r.t. the subdivision of $\Omega$ as indicated in Fig. 2.

**Fig. 3:**
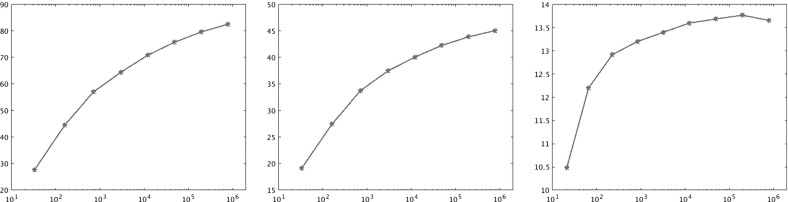
Condition numbers of $\langle \nabla \Psi ^\mathscr {U}_N,\nabla \Psi ^\mathscr {U}_N\rangle _{L_2(\Omega )^2}$, $\langle \nabla \Psi ^\mathscr {V}_N,\nabla \Psi ^\mathscr {V}_N\rangle _{L_2(\Omega )^2}$, and $\langle \Psi ^{\mathscr {T}_i}_N, \Psi ^{\mathscr {T}_i}_N\rangle _{L_2(\Omega )}$, where $\Psi ^*_N$ is the subset of all wavelets from $\Psi ^*$ up to some level, where *N* denotes its cardinality

On a bounded domain, the three-point basis has actually not be proven to be stable in $L_2(\Omega )$. Although alternative bases are available whose Riesz basis property has been proven, we opted for the three-point basis, because of its efficient implementation and because numerical results indicated that it is stable. In Fig. 3, numerically computed condition numbers are given of sets of all wavelets up to some level.

The continuous piecewise quadratic wavelets are biorthogonal ones with the ‘dual multiresolution analysis’ being the sequence of continuous piecewise linears, zero at the $\partial \Omega$, w.r.t. one additional level of refinement. Details of this basis construction will be reported elsewhere.

We performed the approximate evaluation of $D\mathbf{Q}(\cdot )$ according to (s1)–(s4) and Theorem 5.14 in Sect. 5.3 with some simplifications because of the current homogeneous Dirichlet boundary conditions and sufficiently smooth right-hand side [(s1) and (s4) are void, and in (s2) the boundary term is void]. Taking the parameter $k=1$, it turns out that the approximate evaluation is sufficiently accurate to be used in Step (R) of **awgm** (so we do not perform a loop), as well in the simple fixed point iteration (3.2) with damping $\omega =\frac{1}{4}$ that we use for Step (G). We took the parameter $\gamma$ in Step (G) equal to 0.15 (more precisely, for stopping the iteration we checked whether the norm of the approximate residual, restricted to $\Lambda _{i+1}$, is less or equal to $0.15 \Vert \mathbf{r}_i\Vert$).

For the bulk chasing, i.e. Step (B), we simply collected the indices of the largest entries of the approximate residual $\mathbf{r}_i$ until the norm of the residual restricted to those indices is not less than $0.4 \Vert \mathbf{r}_i\Vert$ (i.e. $\mu _1=0.4$), and then, after adding the indices from the current $\varvec{\Lambda }_i$ to this set, we expand it to an admissible set (cf. Definition 5.11). Although this simple procedure is neither guaranteed to satisfy Condition 3.4 nor (B) for some constant $0<\mu _0\le \mu _1$, we observed that it works satisfactory in practice.

**Fig. 4:**
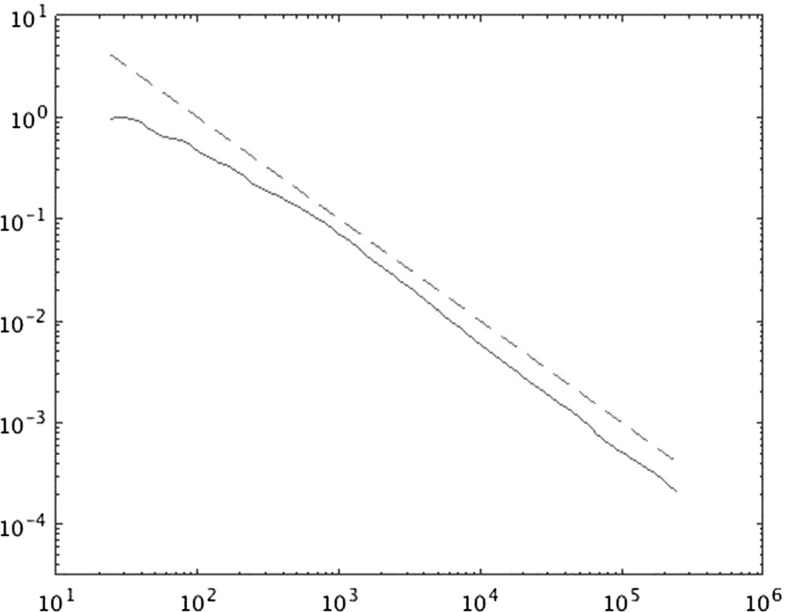
Norm of the (approximate) residual, normalized by the norm of the initial residual, generated by the **awgm** versus the total number of wavelets. The dotted line indicates the best possible slope $-\,1$

In view of the orders 3 and 2 of the bases for $\mathscr {U}$ and $\mathscr {T}$, and the fact the PDE is posed in $n=2$ space dimensions, the best possible convergence rate that can be expected is $\min (\frac{3-1}{2},\frac{2-0}{2})=1$. In Fig. 4, we show the norm of the approximate residual versus the total number of wavelets underlying the approximation for $(u,\vec {\theta })$.

**Fig. 5:**
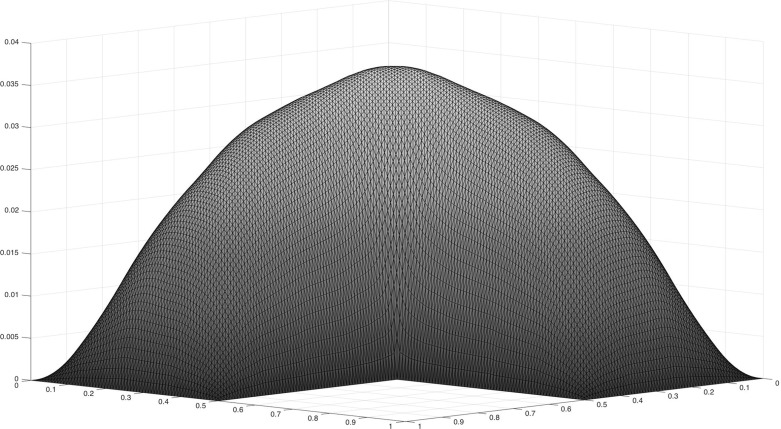
The approximation for *u* from the span of 202 wavelets

**Fig. 6:**
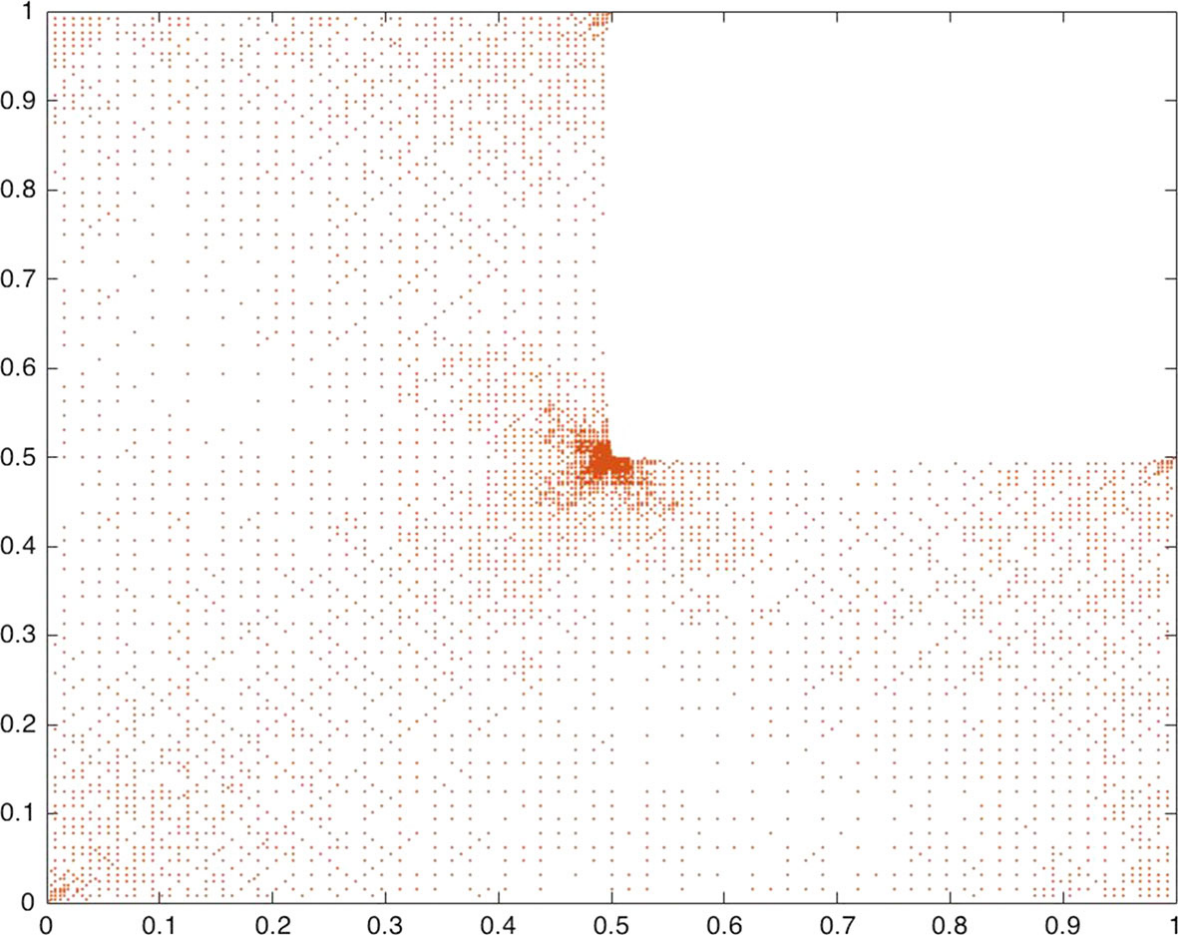
Centers of the supports of the first 10,366 wavelets for the approximation for *u* that were selected by the **awgm**

The norm of the approximate residual is proportional to the $\mathscr {U}\times \mathscr {T}$-norm of the error in the approximation for $(u,\vec {\theta })$. We conclude that it decays with the best possible rate. Moreover, we observed that the computing times scale linearly with the number of unknowns. Throughout the iteration, the number of wavelets for the approximation for *u* is of the same order as the number of wavelets for the approximation for $\vec {\theta }$. The maximum level that is reached at the end of the computations is 26 for *u* and 28 for $\vec {\theta }$. An approximate solution is illustrated in Fig. 5. Centers of the supports of the wavelets that were selected for the approximation for *u* are illustrated in Fig. 6.

Finally, in order to get an impression of the condition number of the bi-infinite linearized normal equations that eventually we are solving, we consider the Poisson equation, i.e. (6.1) without the $u^3$ term. We are interested in the spectral condition number of the ‘system matrix’ given by

6.2 $$\begin{aligned}&\left[ \begin{array}{lll} \langle \nabla \Psi ^\mathscr {U}, \nabla \Psi ^\mathscr {U}\rangle _{L_2(\Omega )^2} &{}\quad -\,\langle \partial _1 \Psi ^\mathscr {U}, \Psi ^{\mathscr {T}_1} \rangle _{L_2(\Omega )} &{}\quad -\,\langle \partial _2 \Psi ^\mathscr {U}, \Psi ^{\mathscr {T}_2} \rangle _{L_2(\Omega )}\\ -\,\langle \Psi ^{\mathscr {T}_1}, \partial _1 \Psi ^\mathscr {U}\rangle _{L_2(\Omega )} &{}\quad \langle \Psi ^{\mathscr {T}_1}, \Psi ^{\mathscr {T}_1} \rangle _{L_2(\Omega )} &{}\quad 0\\ -\langle \Psi ^{\mathscr {T}_2}, \partial _2 \Psi ^\mathscr {U}\rangle _{L_2(\Omega )} &{}\quad 0 &{}\quad \langle \Psi ^{\mathscr {T}_1}, \Psi ^{\mathscr {T}_1} \rangle _{L_2(\Omega )}\\ \end{array}\right] \nonumber \\&\quad +\left[ \begin{array}{lll} 0 &{}\quad 0 &{}\quad 0 \\ 0 &{}\quad \langle \Psi ^{\mathscr {T}_1}, \partial _1 \Psi ^\mathscr {V}\rangle _{L_2(\Omega )} \langle \partial _1 \Psi ^{\mathscr {V}}, \Psi ^{\mathscr {T}_1} \rangle _{L_2(\Omega )} &{}\quad \langle \Psi ^{\mathscr {T}_1}, \partial _1 \Psi ^\mathscr {V}\rangle _{L_2(\Omega )} \langle \partial _2 \Psi ^{\mathscr {V}}, \Psi ^{\mathscr {T}_2} \rangle _{L_2(\Omega )}\\ 0 &{}\quad \langle \Psi ^{\mathscr {T}_2}, \partial _2 \Psi ^\mathscr {V}\rangle _{L_2(\Omega )} \langle \partial _1 \Psi ^{\mathscr {V}}, \Psi ^{\mathscr {T}_1} \rangle _{L_2(\Omega )} &{}\quad \langle \Psi ^{\mathscr {T}_2}, \partial _2 \Psi ^\mathscr {V}\rangle _{L_2(\Omega )} \langle \partial _2 \Psi ^{\mathscr {V}}, \Psi ^{\mathscr {T}_2} \rangle _{L_2(\Omega )}\\ \end{array}\right] \end{aligned}$$

**Fig. 7:**
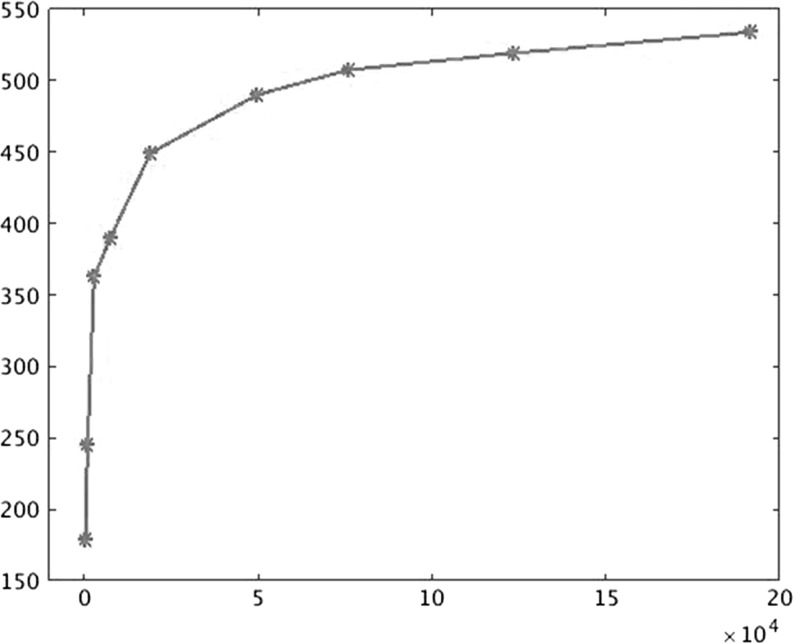
Condition numbers of the (approximate) Galerkin system matrices versus $\# \Lambda$

To that end we numerically approximated the condition numbers of the finite square blocks of rows and columns with indices in $\Lambda$, with $\Lambda$ running over the wavelet index sets that were created by the **awgm**. Even such a finite block cannot be evaluated exactly, because it still involves the infinite collection $\Psi ^\mathscr {V}$. Given a $\Lambda$, we restricted this collection to the wavelets with indices in $\Lambda ^\mathscr {V}(\mathcal {T}(\Lambda ),k)$ as defined by Proposition 5.6 and Definition 5.8, where, as always, we take $k=1$. The resulting matrix is exactly the one that we approximately invert in Step (G) by the fixed point iteration. The computed condition numbers are given in Fig. 7. We performed the same computation also for $k=2$, so with an enlarged set of wavelet indices from the basis $\Psi ^\mathscr {V}$, and found nearly indistinguishable results. We may conclude that for $\Lambda \rightarrow \infty$, the given numbers give accurate approximations for the condition number of the matrix in (6.2).

## Stationary Navier–Stokes equations

For $n \in \{2,3,4\}$, let $\Omega \subset \mathbb {R}^n$ be a bounded Lipschitz domain. The stationary Navier–Stokes equations in velocity–pressure formulation and with no-slip boundary conditions are given byIn order to obtain, in any case in the linear Stokes case, results that hold uniformly in $\nu >0$, one may equip the spaces for velocities and pressure with $\nu$-dependent norms. The equivalent, but notationally more convenient approach that we will follow is to keep the standard norms, but to make the substitutions $\vec {\breve{u}}=\sqrt{\nu }\, \vec {u}$, $\breve{p}=\frac{1}{\sqrt{\nu }} p$, $\vec {\breve{f}}=\frac{1}{\sqrt{\nu }} \vec {f}$, and $\breve{g}=\sqrt{\nu } \, g$. For convenience dropping the 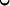-accents, the equations for the new unknowns read asIn variational form they read as finding 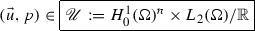 such that for some $(\vec {f},g) \in \mathscr {U}'$,($(\vec {v},q) \in$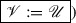.

It is known that $G:\mathscr {U}\rightarrow \mathscr {U}'$, and that a solution $(\vec {u},p)$ exists (see e.g. [24, Ch. IV]). Furthermore, *G* is two times differentiable with its second derivative being constant. We will *assume* that $DG(\vec {u},p) \in \mathcal {L}(\mathscr {U},\mathscr {U}')$ is a homeomorphism with its range, so that each of the conditions (i)–(iii) from Sect. 2 are satisfied. The latter is known to hold true, with its range being equal to $\mathscr {U}'$, when $\vec {f}$ is sufficiently small, in which case the solution $(\vec {u},p)$ is also unique (e.g. see [24, Ch. IV]). For the linear case, so without the term $\nu ^{-3/2} (\vec {u}\cdot \nabla )\vec {u}$, thanks to our re-scaling, $DG(\vec {u},p)=G \in \mathcal {L}\mathrm {is}(\mathscr {U},\mathscr {U}')$, and is independent of $\nu$.

Using the framework outlined in Sect. 2, we write this second order elliptic PDE as a first order system least squares problem. There are different possibilities to do so.

### Velocity–pressure–velocity gradient formulation

With 
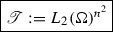
, we define

$$\begin{aligned} G_1 \in \mathcal {L}(\mathscr {T},\mathscr {U}'),\qquad G_2 \in \mathcal {L}(\mathscr {U},\mathscr {T}), \end{aligned}$$

by

$$\begin{aligned} G_2 (\vec {u},p)=\nabla \vec {u},\qquad (G_1 \underline{\theta })(\vec {v},q)=\int _\Omega \underline{\theta } : \nabla \vec {v} \,d x \end{aligned}$$

The results from Sect. 2 show that the solution $(\vec {u},p)$ can be found as the first components of the minimizer $(\vec {u},p, \underline{\theta }) \in \mathscr {U}\times \mathscr {T}$ of

7.1 $$\begin{aligned} \begin{aligned}&Q(\vec {u},p, \underline{\theta }):={\textstyle \frac{1}{2}}\Big (\big \Vert \vec {v} \mapsto \int _\Omega \underline{\theta } : \nabla \vec {v}-p {{\mathrm{div}}}\vec {v}+\nu ^{-3/2} (\vec {u}\cdot \nabla )\vec {u} \cdot \vec {v}-\vec {f}\cdot \vec {v}\Vert _{H^{-1}(\Omega )^n}^2\\&\quad +\Vert {{\mathrm{div}}}\vec {u}-g\Vert _{L_2(\Omega )}^2+\Vert \underline{\theta }- \nabla \vec {u}\Vert _{L_2(\Omega )^{n^2}}^2\Big ), \end{aligned} \end{aligned}$$

and so as the solution of the normal equations $DQ(\vec {u},p,\underline{\theta })=0$. Here we have used that on $H^1_0(\Omega )^n$, $\Vert {{\mathrm{div}}}\cdot \Vert _{(L_2(\Omega )/\mathbb {R})'}=\Vert {{\mathrm{div}}}\cdot \Vert _{L_2(\Omega )}$. Following [3], we call $\underline{\theta }=\nabla \vec {u}$ the velocity gradient. As follows from Sect. 2, these normal equations are well-posed in the sense that they satisfy (1)–(4). This gives us an alternative, effortless proof for [15, Thm. 3.1].

To deal with the ‘unpractical’ norm on $H^{-1}(\Omega )^n$, we equip $H^1_0(\Omega )^n$ with some wavelet Riesz basis

$$\begin{aligned} \Psi ^{(\hat{H}^1_0)^n}=\left\{ \psi _\lambda ^{(\hat{H}^1_0)^n}:\lambda \in \vee _{(\hat{H}^1_0)^n}\right\} , \end{aligned}$$

and replace, in the definition of *Q*, the norm on its dual by the equivalent norm defined by $\Vert \vec {h}(\Psi ^{(\hat{H}^1_0)^n})\Vert$ for $\vec {h} \in H^{-1}(\Omega )^n$.

Next, after equipping $* \in \{H^1_0(\Omega )^n,L_2(\Omega )/\mathbb {R},L_2(\Omega )^{n^2}\}$ with a Riesz basis $\Psi ^{*}=\{\psi _\lambda ^{*}:\lambda \in \vee _{*}\}$, and so $H^1_0(\Omega )^n\times L_2(\Omega )/\mathbb {R}\times L_2(\Omega )^{n^2}$ with

$$\begin{aligned} \Psi :=(\Psi ^{(H^1_0)^n},0_{L_2/\mathbb {R}},0_{L_2^{n^2}}) \cup (0_{(H^1_0)^n},\Psi ^{L_2/\mathbb {R}},0_{L_2^{n^2}}) \cup (0_{(H^1_0)^n},0_{L_2/\mathbb {R}},\Psi ^{L_2^{n^2}}), \end{aligned}$$

with index set $\vee :=\vee _{(H^1_0)^n}\cup \vee _{L_2/\mathbb {R}}\cup \vee _{L_2^{n^2}}$, we apply the **awgm** to the resulting system

$$\begin{aligned}&D\mathbf{Q}([\mathbf{{u}}^\top ,\mathbf{{p}}^\top ,\varvec{{\theta }}^\top ]^\top )=\left[ \begin{array}{@{}l@{}} \langle {{\mathrm{div}}}\, \Psi ^{(H^1_0)^n}, {{\mathrm{div}}}\, \vec {{u}} -g \rangle _{L_2(\Omega )}\\ 0_{\vee _{L_2/\mathbb {R}}}\\ 0_{\vee _{L_2^{n^2}}} \end{array} \right] \\&\quad +\,\left[ \begin{array}{@{}l@{}} \langle \nabla \Psi ^{(H^1_0)^n}, \nabla \vec {{u}}-\underline{{\theta }}\rangle _{L_2(\Omega )^{n^2}}\\ 0_{\vee _{L_2/\mathbb {R}}}\\ \langle \Psi ^{L_2^{n^2}}, \underline{{\theta }} - \nabla \vec {{u}}\rangle _{L_2(\Omega )^{n^2}} \end{array} \right] \\&\quad +\left[ \begin{array}{@{}l@{}} \langle \frac{(\vec {{u}}\cdot \nabla ) \Psi ^{(H^1_0)^n}+(\Psi ^{(H^1_0)^n}\cdot \nabla ) \vec {{u}}}{\nu ^{3/2}},\Psi ^{(\hat{H}^1_0)^n}\rangle _{L_2(\Omega )^n}\\ -\,\langle \Psi ^{L_2/\mathbb {R}},{{\mathrm{div}}}\,\Psi ^{(\hat{H}^1_0)^n} \rangle _{L_2(\Omega )} \\ \langle \Psi ^{L_2^{n^2}}, \nabla \Psi ^{(\hat{H}^1_0)^n} \rangle _{L_2(\Omega )^{n^2}} \end{array} \right] \Big \{\langle \Psi ^{(\hat{H}^1_0)^n},{\textstyle \frac{(\vec {{u}}\cdot \nabla ) \vec {{u}}}{\nu ^{3/2}}}-\vec {f}\rangle _{L_2(\Omega )^n}\\&\quad +\,\langle \nabla \Psi ^{(\hat{H}^1_0)^n}, \underline{\theta }\rangle _{L_2(\Omega )^{n^2}}- \langle {{\mathrm{div}}}\, \Psi ^{(\hat{H}^1_0)^n}, p\rangle _{L_2(\Omega )} \Big \} =0. \end{aligned}$$

To express the three terms in $\vec {v} \mapsto \langle \vec {v},\nu ^{-3/2}(\vec {{u}}\cdot \nabla ) \vec {{u}}-\vec {f}\rangle _{L_2(\Omega )^n}+ \langle \nabla \vec {v}, \underline{\theta }\rangle _{L_2(\Omega )^{n^2}}- \langle {{\mathrm{div}}}\vec {v}, p\rangle _{L_2(\Omega )} \in H^{-1}(\Omega )^n$ w.r.t. one dictionary, similarly to Sect. 1.4 we impose the additional, but in applications easily realizable conditions that

7.2 $$\begin{aligned} \Psi ^{L_2/\mathbb {R}} \subset H^1(\Omega ),\quad \Psi ^{L_2^{n^2}} \subset H({{\mathrm{div}}};\Omega )^n. \end{aligned}$$

Then for finitely supported approximations $[{\tilde{\mathbf{u}}}^\top ,{\tilde{\mathbf{p}}}^\top ,\varvec{\tilde{\theta }}^\top ]^\top$ to $[\mathbf{u}^\top ,\mathbf{p}^\top ,\varvec{\theta }^\top ]^\top$, for $(\vec {\tilde{u}},\tilde{p},\underline{\tilde{\theta }}):=[{\tilde{\mathbf{u}}}^\top ,{\tilde{\mathbf{p}}}^\top ,\varvec{\tilde{\theta }}^\top ] \Psi \in H^1_0(\Omega )^n \times H^1(\Omega ) \times H({{\mathrm{div}}};\Omega )^n$, we have 
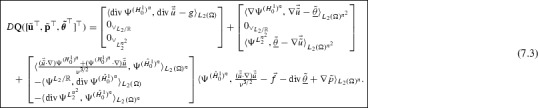
 Each of the terms ${{\mathrm{div}}}\vec {\tilde{u}}-g$, $\nabla \vec {\tilde{u}}-\underline{\tilde{\theta }}$ , $\nu ^{-3/2} (\vec {\tilde{u}}\cdot \nabla ) \vec {\tilde{u}}-\vec {f}-{{\mathrm{div}}}\underline{\tilde{\theta }}+\nabla \tilde{p}$ correspond, in strong form, to a term of the least squares functional, and therefore their norms can be bounded by a multiple of the norm of the residual, which is the basis of our approximate residual evaluation.

This approximate residual evaluation follows the same lines as with the elliptic problem from Sect. 5. Actually, things are easier here because we assume homogeneous boundary conditions. Selecting the Riesz bases for the Cartesian products $H^1_0(\Omega )^n$ and $L_2(\Omega )^{n^2}$ of canonical form, we assume that all scalar-valued bases $\Psi ^*$ for $*\in \{\hat{H}^1_0, H^1_0,L_2/\mathbb {R},L_2\}$ satisfy the assumptions that were made in Sect. 5.2, in particular ($w_{1}$)–($w_{4}$). Let $\Lambda :=\mathrm{supp}\,[{\tilde{\mathbf{u}}}^\top ,{\tilde{\mathbf{p}}}^\top ,\varvec{\tilde{\theta }}^\top ]^\top$ be admissible, i.e., $\Lambda \cap \vee _*$ are trees.

1. Find a tiling ${\mathcal {T}}(\varepsilon ) \subset \mathcal {O}_\Omega$, such that
  $$\begin{aligned} \inf _{\vec {f}_\varepsilon \in \mathcal {P}_m({\mathcal {T}}(\varepsilon ))^n,\,g_\varepsilon \in \mathcal {P}_m({\mathcal {T}}(\varepsilon ))/\mathbb {R}} \Vert \vec {f}-\vec {f}_\varepsilon \Vert _{H^{-1}(\Omega )^n} +\Vert g-g_\varepsilon \Vert _{L_2(\Omega )} \le \varepsilon . \end{aligned}$$
  If $[\mathbf{{u}}^\top ,\mathbf{{p}}^\top ,\varvec{{\theta }}^\top ]^\top \in \mathcal {A}^s$, then such a tiling exists with $\# {\mathcal {T}}(\varepsilon ) \lesssim \varepsilon ^{-1/s}$. Set ${\mathcal {T}}(\Lambda ,\varepsilon ):={\mathcal {T}}(\Lambda ) \oplus {\mathcal {T}}(\varepsilon )$.
2.
  - Approximate $$\mathbf{r}^{(\frac{1}{2})}_1:=\langle \Psi ^{(\hat{H}^1_0)^n},\nu ^{-3/2} (\vec {\tilde{u}}\cdot \nabla ) \vec {\tilde{u}}-\vec {f}-{{\mathrm{div}}}\underline{\tilde{\theta }}+\nabla \tilde{p} \rangle _{L_2(\Omega )^n}$$ by $${\tilde{\mathbf{r}}}^{(\frac{1}{2})}_1:=\mathbf{r}^{(\frac{1}{2})}_1|_{\Lambda ^{(\hat{H}^1_0)^n}({\mathcal {T}}(\Lambda ,\varepsilon ),k)}$$.
  - With $$\tilde{r}_1^{(\frac{1}{2})}:=({\tilde{\mathbf{r}}}^{(\frac{1}{2})}_1)^\top \Psi ^{(H^1_0)^n}$$, approximate
    $$\begin{aligned} \mathbf{r}_1=\left[ \begin{array}{@{}l@{}} \mathbf{r}_{11} \\ \mathbf{r}_{12}\\ \mathbf{r}_{13}\end{array} \right] := \left[ \begin{array}{@{}l@{}} \left\langle \frac{(\vec {\tilde{u}}\cdot \nabla ) \Psi ^{(H^1_0)^n}+(\Psi ^{(H^1_0)^n}\cdot \nabla ) \vec {\tilde{u}}}{\nu ^{3/2}},\tilde{r}_1^{(\frac{1}{2})}\right\rangle _{L_2(\Omega )^n}\\ -\,\left\langle \Psi ^{L_2/\mathbb {R}},{{\mathrm{div}}}\tilde{r}_1^{(\frac{1}{2})} \right\rangle _{L_2(\Omega )} \\ -\,\left\langle {{\mathrm{div}}}\, \Psi ^{L_2^{n^2}}, \tilde{r}_1^{(\frac{1}{2})} \right\rangle _{L_2(\Omega )^n} \end{array} \right] \end{aligned}$$
    by $${\tilde{\mathbf{r}}}_1:=\mathbf{r}_1|_{\Lambda ({\mathcal {T}}(\Lambda ^{(H^1_0)^n}({\mathcal {T}}(\Lambda ,\varepsilon ),k)),k)}$$.
3. Approximate
  $$\begin{aligned} \mathbf{r}_2=\left[ \begin{array}{@{}l@{}} \mathbf{r}_{21} \\ \mathbf{r}_{22}\\ \mathbf{r}_{23}\end{array} \right] :=\left[ \begin{array}{@{}l@{}} \langle {{\mathrm{div}}}\, \Psi ^{(H^1_0)^n}, {{\mathrm{div}}}\vec {\tilde{u}}-g \rangle _{L_2(\Omega )}\\ 0_{\vee _{L_2/\mathbb {R}}}\\ 0_{\vee _{L_2^{n^2}}} \end{array} \right] \text { by }{\tilde{\mathbf{r}}}_2:=\mathbf{r}_2|_{\Lambda ({\mathcal {T}}(\Lambda ,\varepsilon ),k)} \end{aligned}$$
4. Approximate
  $$\begin{aligned} \mathbf{r}_3=\left[ \begin{array}{@{}l@{}} \mathbf{r}_{31} \\ \mathbf{r}_{32}\\ \mathbf{r}_{33}\end{array} \right] := \left[ \begin{array}{@{}l@{}} \langle \nabla \Psi ^{(H^1_0)^n}, \nabla \vec {\tilde{u}}-\underline{\tilde{\theta }} \rangle _{L_2(\Omega )^{n^2}}\\ 0_{\vee _{L_2/\mathbb {R}}}\\ \langle \Psi ^{L_2^{n^2}}, \underline{\tilde{\theta }} - \nabla \vec {\tilde{u}}\rangle _{L_2(\Omega )^{n^2}} \end{array}\right] \text { by } {\tilde{\mathbf{r}}}_3:=\mathbf{r}_3|_{\Lambda ({\mathcal {T}}(\Lambda ,\varepsilon ),k)}. \end{aligned}$$

The same arguments (actually a subset) that led to Theorem 5.14 show the following theorem.

#### Theorem 7.1

For an admissible $\Lambda \subset \vee$, $[{\tilde{\mathbf{u}}}^\top ,{\tilde{\mathbf{p}}}^\top ,\varvec{\tilde{\theta }}^\top ]^\top \in \ell _2(\Lambda )$ with $(\vec {\tilde{u}},\tilde{p},\underline{\tilde{\theta }})$ sufficiently close to $(\vec {u},p,\underline{\theta })$, and an $\varepsilon >0$, consider the steps (s1)-(s4). With $s>0$ such that $[{\tilde{\mathbf{u}}}^\top ,{\tilde{\mathbf{p}}}^\top ,\varvec{\tilde{\theta }}^\top ]^\top \in \mathcal {A}^s$, it holds that

$$\begin{aligned}&\Vert D\mathbf{Q}([{\tilde{\mathbf{u}}}^\top ,{\tilde{\mathbf{p}}}^\top , \varvec{\tilde{\theta }}^\top ]^\top )-({\tilde{\mathbf{r}}}_1+{\tilde{\mathbf{r}}}_2+{\tilde{\mathbf{r}}}_3)\Vert \\&\quad \lesssim 2^{-k/2} (\Vert \vec {u}-\vec {\tilde{u}}\Vert _{H^1_0(\Omega )^n}+\Vert p-\tilde{p}\Vert _{L_2(\Omega )} +\Vert \underline{\theta }-\underline{\tilde{\theta }}\Vert _{L_2(\Omega )^{n^2}})+\varepsilon , \end{aligned}$$

where the computation of ${\tilde{\mathbf{r}}}_1+{\tilde{\mathbf{r}}}_2+{\tilde{\mathbf{r}}}_3$ requires ${\mathcal {O}}(\# \Lambda +\varepsilon ^{-1/s})$ operations. So by taking *k* sufficiently large, Condition 3.5* is satisfied.

We conclude that the **awgm** is an optimal solver for the stationary Navier–Stokes equations in the form $D\mathbf{Q}([\mathbf{{u}}^\top ,\mathbf{{p}}^\top ,\varvec{{\theta }}^\top ]^\top )=0$ resulting from the velocity–pressure–velocity gradient formulation. Obviously, we cannot claim or even expect that this holds true uniformly in a vanishing viscosity parameter $\nu$. This because in the limit already well-posedness of $DG(\vec {u},p)$ cannot be expected.

### Velocity–pressure–vorticity formulation

Restricting to $n \in \{2,3\}$, we set 
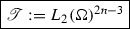
, and define

$$\begin{aligned} G_1 \in \mathcal {L}(\mathscr {T},\mathscr {U}'),\qquad G_2 \in \mathcal {L}(\mathscr {U},\mathscr {T}), \end{aligned}$$

by

$$\begin{aligned} G_2 (\vec {u},p)= {{\mathrm{curl}}}\vec {u},\qquad (G_1 \vec {\omega } )(\vec {v},q)=\int _\Omega \vec {\omega } \cdot {{\mathrm{curl}}}\vec {v} \,d x \end{aligned}$$

where for $n=2$, ${{\mathrm{curl}}}$ should be read as the scalar-valued operator $\vec {v} \mapsto \partial _x v_2-\partial _y v_1$. (and so $\vec {\omega }\cdot {{\mathrm{curl}}}\vec {u}$ as $\omega {{\mathrm{curl}}}\vec {u}$). The (formal) adjoint ${{\mathrm{curl}}}'$ equals ${{\mathrm{curl}}}$ for $n=3$, whereas for $n=2$ it is $v \mapsto [\partial _y v,-\partial _x v]^\top$.

Since a vector field in the current space $\mathscr {T}$ has $2n-3$ components, instead of $n^2$ as in the previous subsection, the first order system formulation studied in this subsection is more attractive. As we will see, later in its derivation it will be needed that $g=0$, i.e., ${{\mathrm{div}}}\vec {u}=0$.

Using that on $H_0^1(\Omega )^n \times H_0^1(\Omega )^n$, $\int _\Omega \nabla \vec {u} :\nabla \vec {v}-{{\mathrm{div}}}\vec {u} {{\mathrm{div}}}\vec {v}-{{\mathrm{curl}}}\vec {u} \cdot {{\mathrm{curl}}}\vec {v} \,dx=0$, the results from Sect. 2 show that the solution $(\vec {u},p)$ can be found as the first component of the solution in $\mathscr {U}\times \mathscr {T}$ of the system

$$\begin{aligned}&\vec {H}_1(\vec {u},p,\vec {\omega }):= \Big ( (\vec {v},q) \mapsto \int _\Omega \vec {\omega } \cdot {{\mathrm{curl}}}\vec {v}+ {{\mathrm{div}}}\vec {u} {{\mathrm{div}}}\vec {v}-p {{\mathrm{div}}}\vec {v} +\nu ^{-3/2}(\vec {u}\cdot \nabla ) \vec {u} \cdot \vec {v}\\&\quad +\,q ({{\mathrm{div}}}\vec {u}-g) -\vec {f}\cdot \vec {v} \,dx,\vec {\omega }-{{\mathrm{curl}}}\vec {u}\Big )=\vec {0} \end{aligned}$$

on $\mathscr {U}' \times \mathscr {T}$, being a minimizer of

$$\begin{aligned} \begin{aligned}&Q_1(\vec {u},p,\vec {\omega }):={\textstyle \frac{1}{2}}\Big (\big \Vert \vec {v} \mapsto \int _\Omega \vec {\omega } \cdot {{\mathrm{curl}}}\vec {v}+ {{\mathrm{div}}}\vec {u} {{\mathrm{div}}}\vec {v}-p {{\mathrm{div}}}\vec {v}\\&\quad +\,{\textstyle \frac{(\vec {u}\cdot \nabla ) \vec {u} \cdot \vec {v}}{\nu ^{3/2}}} -\vec {f}\cdot \vec {v} \,dx \Vert _{H^{-1}(\Omega )^n}^2\\&\quad +\,\Vert {{\mathrm{div}}}\vec {u}-g\Vert _{L_2(\Omega )}^2+\Vert \vec {\omega }- {{\mathrm{curl}}}\vec {u}\Vert _{L_2(\Omega )^{2n-3}}^2\Big ). \end{aligned} \end{aligned}$$

The function $\vec {\omega }={{\mathrm{curl}}}\vec {u}$ is known as the vorticity.

Since *G* satisfies (i)–(iii), $\vec {H}_1$ satisfies (a)–(c), and so by Lemma 2.6,

7.4 $$\begin{aligned} Q_1(\vec {\tilde{u}},\tilde{p},\vec {\tilde{\omega }}) \eqsim \Vert (\vec {\tilde{u}},\tilde{p}, \vec {\tilde{\omega }})-(\vec {u},{p},\vec {\omega })\Vert ^2_{\mathscr {U}\times \mathscr {T}} \end{aligned}$$

for $(\vec {\tilde{u}},\tilde{p},\vec {\tilde{\omega }})$ in a neighborhood of $(\vec {u},{p},\vec {{\omega }})$.

From here on, we assume that

$$\begin{aligned} g=0, \end{aligned}$$

so that the velocities component of the exact solution is divergence-free. This will allow us to get rid of the second order term $\nabla {{\mathrm{div}}}\vec {u}$ in the definition of $\vec {H}_1$. We define

$$\begin{aligned} \vec {H}_2(\vec {u},p,\vec {\omega }):= & {} \Big ( (\vec {v},q) \mapsto \int _\Omega \vec {\omega } \cdot {{\mathrm{curl}}}\vec {v} -p {{\mathrm{div}}}\vec {v} +{\textstyle \frac{(\vec {u}\cdot \nabla ) \vec {u} \cdot \vec {v}}{\nu ^{3/2}}}\\&\quad +\,q {{\mathrm{div}}}\vec {u} -\vec {f}\cdot \vec {v} \,dx,\vec {\omega }-{{\mathrm{curl}}}\vec {u}\Big ) \end{aligned}$$

with corresponding quadratic functional

$$\begin{aligned} \begin{aligned} Q_2(\vec {u},p,\vec {\omega })&:={\textstyle \frac{1}{2}}\Big (\big \Vert \vec {v} \mapsto \int _\Omega \vec {\omega } \cdot {{\mathrm{curl}}}\vec {v}-p {{\mathrm{div}}}\vec {v} +{\textstyle \frac{(\vec {u}\cdot \nabla ) \vec {u} \cdot \vec {v}}{\nu ^{3/2}}} -\vec {f}\cdot \vec {v} \,dx \Vert _{H^{-1}(\Omega )^n}^2\\&\qquad +\,\Vert {{\mathrm{div}}}\vec {u}\Vert _{L_2(\Omega )}^2+\Vert \vec {\omega }- {{\mathrm{curl}}}\vec {u}\Vert _{L_2(\Omega )^{2n-3}}^2\Big ). \end{aligned} \end{aligned}$$

Clearly the solution of $\vec {H}_1(\vec {u},p,\vec {\omega })=0$ is a solution of $\vec {H}_2(\vec {u},p,\vec {\omega })=0$ (a), and $\vec {H}_2$ is two times continuously differentiable (b). From $\Vert \vec {v} \mapsto \int _\Omega {{\mathrm{div}}}\vec {u} {{\mathrm{div}}}\vec {v}\,dx \Vert _{H^{-1}(\Omega )^n}$ $\lesssim \Vert {{\mathrm{div}}}\vec {u}\Vert _{L_2(\Omega )}$, one infers that $Q_1\lesssim Q_2$ by the triangle inequality, and analogously $Q_2\lesssim Q_1$. Thanks to (7.4), an application of Lemma 2.6 shows that $\vec {H}_2$ satisfies also (c). We conclude that $\vec {H}_2(\vec {u},p,\vec {\omega })$ is a well-posed first order system formulation of $G(\vec {u},p)=0$, and consequently, that $(\vec {u},p,\vec {\omega })$ can be found by solving the normal equations $DQ_2(\vec {u},p,\vec {\omega })=0$, which are well-posed in the sense that they satisfy (1)–(4). This gives us an alternative, effortless proof of [13, Thm. 2.1].

As usual, to deal with the ‘unpractical’ norm on $H^{-1}(\Omega )^n$, we equip $H^1_0(\Omega )^n$ with a wavelet Riesz basis

$$\begin{aligned} \Psi ^{(\hat{H}^1_0)^n}=\left\{ \psi _\lambda ^{(\hat{H}^1_0)^n}:\lambda \in \vee _{(\hat{H}^1_0)^n}\right\} , \end{aligned}$$

and replace, in the definition of $Q_2$, the norm on its dual by the equivalent norm $\Vert \vec {g}(\Psi ^{(H^1_0)^n})\Vert$ for $\vec {g} \in H^{-1}(\Omega )^n$.

Next, after equipping $* \in \{H^1_0(\Omega )^n, L_2(\Omega )/\mathbb {R}, L_2(\Omega )^{2n-3}\}$ with Riesz basis $\Psi ^{*}=\{\psi _\lambda ^{*} :\lambda \in \vee _{*}\}$, and so $H^1_0(\Omega )^n\times L_2(\Omega )/\mathbb {R}\times L_2(\Omega )^{2n-3}$ with

$$\begin{aligned} \Psi :=(\Psi ^{(H^1_0)^n},0_{L_2/\mathbb {R}},0_{L_2^{2n-3}}) \cup (0_{(H^1_0)^n},\Psi ^{L_2/\mathbb {R}},0_{L_2^{2n-3}}) \cup (0_{(H^1_0)^n},0_{L_2/\mathbb {R}},\Psi ^{L_2^{2n-3}}) \end{aligned}$$

with index set $\vee :=\vee _{(H^1_0)^n}\cup \vee _{L_2/\mathbb {R}}\cup \vee _{L_2^{2n-3}}$, we apply the **awgm** to the resulting system

$$\begin{aligned} \begin{aligned}&D\mathbf{Q}_2([\mathbf{{u}}^\top ,\mathbf{{p}}^\top ,\varvec{\omega }^\top ]^\top )=\left[ \begin{array}{@{}l@{}} \langle {{\mathrm{div}}}\, \Psi ^{(H^1_0)^n}, {{\mathrm{div}}}\vec {{u}} \rangle _{L_2(\Omega )}\\ 0_{\vee _{L_2/\mathbb {R}}}\\ 0_{\vee _{L_2^{2n-3}}} \end{array} \right] \\&\quad +\, \left[ \begin{array}{@{}l@{}} \langle {{\mathrm{curl}}}\Psi ^{(H^1_0)^n}, {{\mathrm{curl}}}\vec {{u}}-\vec {\omega }\rangle _{L_2(\Omega )^{2n-3}}\\ 0_{\vee _{L_2/\mathbb {R}}}\\ \langle \Psi ^{L_2^{2n-3}}, \vec {{\omega }} - {{\mathrm{curl}}}\vec {{u}}\rangle _{L_2(\Omega )^{2n-3}} \end{array} \right] \\&\quad +\, \left[ \begin{array}{@{}l@{}} \langle \frac{(\vec {{u}}\cdot \nabla ) \Psi ^{(H^1_0)^n}+(\Psi ^{(H^1_0)^n}\cdot \nabla ) \vec {{u}}}{\nu ^{3/2}},\Psi ^{(\hat{H}^1_0)^n}\rangle _{L_2(\Omega )^n}\\ -\langle \Psi ^{L_2/\mathbb {R}},{{\mathrm{div}}}\,\Psi ^{(\hat{H}^1_0)^n} \rangle _{L_2(\Omega )} \\ \langle \Psi ^{L_2^{2n-3}}, {{\mathrm{curl}}}\Psi ^{(\hat{H}^1_0)^n} \rangle _{L_2(\Omega )^{2n-3}} \end{array} \right] \Big \{\langle \Psi ^{(\hat{H}^1_0)^n},{\textstyle \frac{(\vec {{u}}\cdot \nabla ) \vec {{u}}}{\nu ^{3/2}}}-\vec {f}\rangle _{L_2(\Omega )^n} \\&\quad +\,\langle {{\mathrm{curl}}}\Psi ^{(\hat{H}^1_0)^n}, \vec {\omega }\rangle _{L_2(\Omega )^{2n-3}}- \langle {{\mathrm{div}}}\, \Psi ^{(\hat{H}^1_0)^n}, p\rangle _{L_2(\Omega )} \Big \} =0. \end{aligned} \end{aligned}$$

To express the three terms in $\vec {v} \mapsto \langle \vec {v},\nu ^{-3/2}(\vec {{u}}\cdot \nabla ) \vec {{u}}-\vec {f}\rangle _{L_2(\Omega )^n} + \langle {{\mathrm{curl}}}\vec {v}, \vec {\omega }\rangle _{L_2(\Omega )^{2n-3}}- \langle {{\mathrm{div}}}\vec {v}, p\rangle _{L_2(\Omega )}$ w.r.t. one dictionary, we impose the easily realizable conditions that

$$\begin{aligned} \Psi ^{L_2/\mathbb {R}} \subset H^1(\Omega ),\, \Psi ^{L_2^{2n-3}} \subset H({{\mathrm{curl}}};\Omega ) \end{aligned}$$

Then for finitely supported approximations $[{\tilde{\mathbf{u}}}^\top ,{\tilde{\mathbf{p}}}^\top ,\varvec{\tilde{\omega }}^\top ]^\top$ to $[\mathbf{u}^\top ,\mathbf{p}^\top ,\varvec{\omega }^\top ]^\top$, for $(\vec {\tilde{u}},\tilde{p},\vec {\tilde{\omega }}):=[{\tilde{\mathbf{u}}}^\top ,{\tilde{\mathbf{p}}}^\top ,\varvec{\tilde{\omega }}^\top ] \Psi \in H^1_0(\Omega )^n \times H^1(\Omega ) \times H({{\mathrm{curl}}}';\Omega )$, we have 
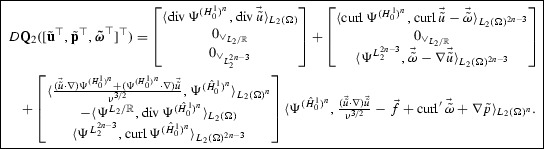


The design of an approximate residual evaluation follows analogous steps as in the previous subsection. Equipping Cartesian products with bases of canonical form, and assuming that the scalar-valued bases $\Psi ^*$ for $*\in \{\hat{H}^1_0, H^1_0,L_2/\mathbb {R},L_2\}$ satisfy ($w_{1}$)–($w_{4}$), and that $[{\tilde{\mathbf{u}}}^\top ,{\tilde{\mathbf{p}}}^\top ,\varvec{\tilde{\omega }}^\top ]^\top$ is supported on an admissible set, four steps fully analogous to (s1)–(s4) in the previous subsection define an approximation scheme that satisfies Condition 3.5*. We conclude that the **awgm** is an optimal solver for the stationary Navier–Stokes equations in the form $D\mathbf{Q}([\mathbf{{u}}^\top ,\mathbf{{p}}^\top ,\varvec{{\theta }}^\top ]^\top )=0$ resulting from the velocity–pressure–vorticity formulation. Again, also here we cannot claim or even expect that this holds true uniformly in a vanishing viscosity parameter $\nu$.

## Conclusion

We have seen that a well-posed (system of) 2nd order PDE(s) can always be formulated as a well-posed 1st order least squares system. The arising dual norm(s) can be replaced by the equivalent $\ell _2$-norm(s) of the wavelet coefficients of the functional. The resulting Euler–Lagrange equations, also known as the (nonlinear) normal equations, can be solved at the best possible rate by the adaptive wavelet Galerkin method. We developed a new approximate residual evaluation scheme that also for semi-linear problems satisfies the condition for optimal computational complexity, and that is quantitatively much more efficient than the usual **apply** scheme. Moreover, regardless of the order of the wavelets, it applies already to wavelet bases that have only one vanishing moment. As applications we discussed optimal solvers for first order least squares reformulations of 2nd order elliptic PDEs with inhomogeneous boundary conditions, and that of the stationary Navier–Stokes equations. In a forthcoming work, we will apply this approach to time-evolution problems.

## Appendix A. Decay estimates

We collect a number of decay estimates that have been used in the proof of Theorem 5.14. Recall the definition of the spaces $\mathscr {U}$ and $\mathscr {V}$ given at the beginning of Sect. 5.1.

The following proposition and subsequent lemma have been used to bound $\Vert \mathbf{r}^{(\frac{1}{2})}_1-{\tilde{\mathbf{r}}}^{(\frac{1}{2})}_1\Vert$. The presence of the boundary integral and the fact that the upper bound that is given depends on the norm of *g* as a whole, and not on norms of $g_1$ and $g_2$ requires a non-standard treatment.

### Proposition A.1

For a tiling ${\mathcal {T}} \subset \mathcal {O}_\Omega$, let $g \in \mathscr {V}_1'$ be of the form

$$\begin{aligned} g(v)=\int _{\Omega } g_1 v \,dx+\int _{\Gamma _{N}} \vec {g}_2 \cdot \mathbf{n}v \,ds, \end{aligned}$$

where $$g_1 \in \mathcal {P}_m(\mathcal {T})$$, $$\vec {g}_2 \in \mathcal {P}_m(\mathcal {T})^n$$. Then

$$\begin{aligned} \big \Vert g(\Psi ^{\mathscr {V}_1}) \big |_{\vee _{\mathscr {V}_1} {\setminus } \Lambda ^{\mathscr {V}_1}(\mathcal {T},k)}\big \Vert \lesssim 2^{-k} \Vert g\Vert _{\mathscr {V}_1'} \end{aligned}$$

(uniform in ${\mathcal {T}}$ and *g*).

### Proof

Since by assumption ($w_{4}$), for $\lambda \in \vee _{\mathscr {V}_1}$ with $|\lambda |>0$ either $\int _\Omega \psi _\lambda ^{\mathscr {V}_1} \,dx =0$ or $\mathrm{dist}(\mathrm{supp}\,\psi _\lambda ^{\mathscr {V}},\Gamma _D) \lesssim 2^{-|\lambda |}$, an application of Friedrich’s or Poincaré’s inequality shows that $\Vert \psi _\lambda ^{\mathscr {V}_1}\Vert _{L_2(\Omega )} \lesssim 2^{-|\lambda |} |\psi _\lambda ^{\mathscr {V}_1}|_{H^1(\Omega )}\eqsim 2^{-|\lambda |}$.

Since by ($w_{1}$)–($w_{2}$), each descendant $\omega ' \in \mathcal {O}_\Omega$ of $\omega \in {\mathcal {T}}$ with $|\omega '|>|\omega |+k$ is intersected by the supports of a uniformly bounded number of $\lambda \in \vee _{\mathscr {V}_1} {\setminus } \Lambda ^{\mathscr {V}_1}(\mathcal {T},k)$ with $|\lambda |=|\omega '|$, we have

A.1 $$\begin{aligned} \begin{aligned} \sum _{\lambda \in \vee _{\mathscr {V}_1} {\setminus } \Lambda ^{\mathscr {V}_1}(\mathcal {T},k)} \Big | \int _\Omega g_1 \psi _\lambda ^{\mathscr {V}_1} \, dx\Big |^2&\le \sum _{\lambda \in \vee _{\mathscr {V}_1} {\setminus } \Lambda ^{\mathscr {V}_1}(\mathcal {T},k)} \sum _{\omega \in {\mathcal {T}}} 4^{-|\lambda |} \Vert g_1\Vert ^2_{L_2(\omega \cap \mathrm{supp}\,\psi ^{\mathscr {V}_1}_\lambda )}\\&\lesssim 4^{-k} \sum _{\omega \in {\mathcal {T}}} 4^{-|\omega |} \Vert g_1\Vert _{L_2(\omega )}^2, \end{aligned} \end{aligned}$$

A standard homogeneity argument shows that for $\omega \in {\mathcal {T}}$ and $v \in H^1(\omega )$, $\Vert v\Vert _{L_2(\partial \omega )} \lesssim 2^{-|\omega |/2}(|v|_{H^1(\omega )}+2^{|\omega |}\Vert v\Vert _{L_2(\omega )})$, so that $\Vert \psi ^{\mathscr {V}_1}_\lambda \Vert _{L_2(\Gamma _N)}\lesssim 2^{-|\lambda |/2}$. Writing $\partial \omega \cap \Gamma _N$ as $\partial \omega _N$, the arguments that led to (A.1) show that

A.2 $$\begin{aligned} \begin{aligned} \sum _{\lambda \in \vee _{\mathscr {V}_1} {\setminus } \Lambda ^{\mathscr {V}_1}(\mathcal {T},k)} \Big | \int _{\Gamma _N} \vec {g}_2 \cdot \mathbf{n} \psi _\lambda ^{\mathscr {V}_1} \, ds\Big |^2&\lesssim \sum _{\lambda \in \vee _{\mathscr {V}_1} {\setminus } \Lambda ^{\mathscr {V}_1}(\mathcal {T},k)} \sum _{\omega \in {\mathcal {T}}} 2^{-|\lambda |} \Vert \vec {g}_2 \cdot \mathbf{n}\Vert ^2_{L_2(\omega _N \cap \mathrm{supp}\,\psi ^{\mathscr {V}_1}_\lambda )}\\&\lesssim 2^{-k} \sum _{\omega \in {\mathcal {T}}} 2^{-|\omega |} \Vert \vec {g}_2 \cdot \mathbf{n}\Vert _{L_2(\omega _N)}^2. \end{aligned} \end{aligned}$$

By combining (A.1), (A.2) with Lemma A.2, the proof is completed unless $\mathscr {U}=\mathscr {V}_1=H^1(\Omega )/\mathbb {R}$. In the latter case, define $\bar{g}_1:=g_1-{{\mathrm{meas}}}(\Omega )^{-1} g(\mathbb {1})$ and $\bar{g}(v):=\int _{\Omega } \bar{g}_1 v \,dx+\int _{\Gamma _{N}} \vec {g}_2 \cdot \mathbf{n}v \,ds$. From $g(\Psi ^{\mathscr {V}_1}) \big |_{\vee _{\mathscr {V}_1} {\setminus } \Lambda ^{\mathscr {V}_1}(\mathcal {T},k)}=\bar{g}(\Psi ^{\mathscr {V}_1}) \big |_{\vee _{\mathscr {V}_1} {\setminus } \Lambda ^{\mathscr {V}_1}(\mathcal {T},k)}$, and $\Vert g\Vert _{\mathscr {V}_1'}=\Vert \bar{g}\Vert _{\mathscr {V}_1'}$, applications of (A.1), (A.2) and that of Lemma A.2 to $\bar{g}$ complete the proof in this case. $\square$

### Lemma A.2

In the situation of Proposition A.1, with additionally $g(\mathbb {1})=0$ when $\mathscr {U}=\mathscr {V}_1=H^1(\Omega )/\mathbb {R}$, it holds that

$$\begin{aligned} \sum _{\omega \in {\mathcal {T}}} 4^{-|\omega |} \Vert g_1|_{\omega }\Vert _{L_2(\omega )}^2 + 2^{-|\omega |} \Vert \vec {g}_2\cdot \mathbf{n}|_{\omega }\Vert _{L_2(\partial \omega \cap \Gamma _{N})}^2 \lesssim \Vert g\Vert _{\mathscr {V}'}^2. \end{aligned}$$

### Proof

Thanks to the uniform shape regularity condition, for any $\omega \in \mathcal {O}_\Omega$, there exists a $V_\omega \subset H^1_0(\omega )$ such that

$$\begin{aligned} \Vert v\Vert _{H^1(\omega )}&\lesssim 2^{|\omega |} \Vert v\Vert _{L_2(\omega )} \quad (v \in V_\omega ),\\ \Vert p\Vert _{L_2(\omega )}&\lesssim \sup _{0 \ne v \in V_\omega } \frac{\int _\omega p v\,dx}{\Vert v\Vert _{L_2(\omega )}} \quad (p \in \mathcal {P}_m(\omega )). \end{aligned}$$

For each $\omega \in {\mathcal {T}}$, select $v_\omega \in V_\omega$ with $\Vert g_1|_{\omega }\Vert _{L_2(\omega )} \Vert v_\omega \Vert _{L_2(\omega )} \lesssim \int _\omega g_1|_{\omega } v_\omega \,dx$, and $\Vert v_\omega \Vert _{L_2(\omega )} = 4^{-|\omega |} \Vert g_1|_{\omega }\Vert _{L_2(\omega )}$. Then, with $v=\sum _{\omega \in {\mathcal {T}}} v_\omega \in H^1_0(\Omega )$, we have $\sum _{\omega \in {\mathcal {T}}} 4^{-|\omega |} \Vert g_1|_{\omega }\Vert _{L_2(\omega )}^2 \lesssim \int _\Omega g_1 v \,dx$. By combining this with

$$\begin{aligned} \Vert v\Vert _{H^1(\Omega )}^2=\sum _{\omega \in {\mathcal {T}}} \Vert v_\omega \Vert _{H^1(\omega )}^2 \lesssim \sum _{\omega \in {\mathcal {T}}} 4^{|\omega |} \Vert v_\omega \Vert _{L_2(\omega )}^2= \sum _{\omega \in {\mathcal {T}}} 4^{-|\omega |} \Vert g_1|_{\omega }\Vert _{L_2(\omega )}^2, \end{aligned}$$

and $g(v)=\int _{\Omega } g_1 v \,dx$, we arrive at $\sqrt{\sum _{\omega \in {\mathcal {T}}} 4^{-|\omega |} \Vert g_1|_{\omega }\Vert _{L_2(\omega )}^2} \lesssim \frac{g(v)}{\Vert v\Vert _{H^1(\Omega )}} \le \Vert g\Vert _{\mathscr {V}'}$, with the last inequality being valid when $H^1_0(\Omega ) \subset \mathscr {V}_1$.

Otherwise, when $\mathscr {V}_1=H^1(\Omega )/\mathbb {R}$, we take $\bar{v}=v-\frac{\int _\Omega v \,dx}{{{\mathrm{meas}}}(\Omega )} \mathbb {1} \in \mathscr {V}$. Then $\Vert \bar{v}\Vert _{H^1(\Omega )} \lesssim \Vert v\Vert _{H^1(\Omega )}$, $g(v)=g(\bar{v})$ by assumption, and so $\frac{g(v)}{\Vert v\Vert _{H^1(\Omega )}}\lesssim \frac{g(\bar{v})}{\Vert \bar{v}\Vert _{H^1(\Omega )}}\le \Vert g\Vert _{\mathscr {V}'}$.

For bounding the second term in the statement of the lemma, for a tile $\omega \in \mathcal {O}_\Omega$ we write $\partial \omega \cap \Gamma _N$ as $\partial \omega _N$. Thanks to the uniform shape regularity condition, for any $\omega \in \mathcal {O}_\Omega$ with ${{\mathrm{meas}}}(\partial \omega _N)>0$, there exists a $V_\omega \subset \{v \in H^1(\omega ):v|_{\partial \omega {\setminus } \partial \omega _N}=0\}$ such that

$$\begin{aligned}&\Vert v\Vert _{H^1(\omega )} \lesssim 2^{|\omega |/2} \Vert v\Vert _{L_2(\partial \omega _N)} \quad (v \in V_\omega ),\\&\quad \Vert \vec {p}\cdot \mathbf{n}\Vert _{L_2(\partial \omega _N)} \lesssim \sup _{0 \ne v \in V_\omega } \frac{\int _{\partial \omega _N} \vec {p}\cdot \mathbf{n} v\,ds}{\Vert v\Vert _{L_2(\partial \omega _N)}} \quad (\vec {p} \in \mathcal {P}_m(\omega )^n),\\&\quad V_\omega \perp _{L_2(\omega )} \mathcal {P}_m(\omega ). \end{aligned}$$

For each $\omega \in {\mathcal {T}}$ with ${{\mathrm{meas}}}(\partial \omega _{N})>0$, select $v_\omega \in V_\omega$ for which $\Vert \vec {g}_2|_{\omega }\cdot \mathbf{n}\Vert _{L_2(\partial \omega _N)} \Vert v_\omega \Vert _{L_2(\partial \omega _N)} \lesssim \int _{\partial \omega _N} \vec {g}_2|_{\omega } \cdot \mathbf{n} v_\omega \,ds$, and $\Vert v_\omega \Vert _{L_2(\partial \omega _N)}=$ $2^{-|\omega |} \Vert \vec {g}_2 \cdot \mathbf{n}|_{\omega }\Vert _{L_2(\partial \omega _N)}$. For the other $\omega \in {\mathcal {T}}$, set $v_\omega =0$. Then, for the function $v=\sum _{\omega \in {\mathcal {T}} } v_\omega \in \{w \in H^1(\Omega ):w|_{\Gamma _D}=0\}$, we have

$$\begin{aligned} \sum _{\omega \in {\mathcal {T}} } 2^{-|\omega |} \Vert \vec {g}_2 \cdot \mathbf{n}|_{\omega }\Vert _{L_2(\partial \omega _N)}^2 \lesssim \int _{\Gamma _N} \vec {g}_2\cdot \mathbf{n} v \, ds. \end{aligned}$$

By combining this with

$$\begin{aligned} \Vert v\Vert _{H^1(\Omega )}^2=\sum _{\omega \in {\mathcal {T}}} \Vert v_\omega \Vert _{H^1(\omega )}^2 \lesssim \sum _{\omega \in {\mathcal {T}}} 2^{|\omega |} \Vert v_\omega \Vert _{L_2(\partial \omega _N)}^2=\sum _{\omega \in {\mathcal {T}}} 2^{-|\omega |} \Vert \vec {g}_2 \cdot \mathbf{n}|_{\omega }\Vert _{L_2(\partial \omega _N)}^2, \end{aligned}$$

and $g(v)=\int _{\Gamma _N} \vec {g}_2 \cdot \mathbf{n} v \,dx$, we arrive at

$$\begin{aligned} \sqrt{\sum _{\omega \in {\mathcal {T}}} 2^{-|\omega |} \Vert g_2|_{\omega }\cdot \mathbf{n}\Vert _{L_2(\partial \omega _N)}^2} \lesssim \frac{g(v)}{\Vert v\Vert _{H^1(\Omega )}} \le \Vert g\Vert _{\mathscr {V}'}, \end{aligned}$$

in the case that $\{w \in H^1(\Omega ):w|_{\Gamma _D}=0\} \subset \mathscr {V}_1$.

Otherwise, when $\mathscr {V}_1=H^1(\Omega )/\mathbb {R}$, we take $\bar{v}=v-\frac{\int _\Omega v \,dx}{{{\mathrm{meas}}}(\Omega )} \mathbb {1} \in \mathscr {V}$. Then $\Vert \bar{v}\Vert _{H^1(\Omega )} \lesssim \Vert v\Vert _{H^1(\Omega )}$, $g(v)=g(\bar{v})$ by assumption, and so $\frac{g(v)}{\Vert v\Vert _{H^1(\Omega )}}\lesssim \frac{g(\bar{v})}{\Vert \bar{v}\Vert _{H^1(\Omega )}}\le \Vert g\Vert _{\mathscr {V}'}$. $\square$

An easy version of the proof of Proposition A.1 shows the following result, which has been used to bound $\Vert \mathbf{r}_{11}-{\tilde{\mathbf{r}}}_{11}\Vert$ in the proof of Theorem 5.14.

### Proposition A.3

For a tiling ${\mathcal {T}} \subset \mathcal {O}_\Omega$, and $g \in P_m(\mathcal {T})$, it holds that

$$\begin{aligned} \big \Vert \langle \Psi ^{\mathscr {U}},g\rangle _{L_2(\Omega )}\big |_{\vee _{\mathscr {U}} {\setminus } \Lambda ^{\mathscr {U}}(\mathcal {T},k)}\big \Vert \lesssim 2^{-k} \Vert g\Vert _{\mathscr {U}'} \end{aligned}$$

(uniform in ${\mathcal {T}}$ and *g*).

The statements from the following proposition have been used to bound the terms $\Vert \mathbf{r}_{11}-{\tilde{\mathbf{r}}}_{11}\Vert$ (first statement) and $\Vert \mathbf{r}_2-{\tilde{\mathbf{r}}}_2\Vert$ (both statements) in the proof of Theorem 5.14.

### Proposition A.4

For a tiling ${\mathcal {T}} \subset \mathcal {O}_\Omega$, $g \in P_m(\mathcal {T})$, $1 \le q \le n$, and $\vec {g} \in P_m(\mathcal {T})^n$, it holds that

$$\begin{aligned} \big \Vert \langle \Psi ^{\mathscr {T}_q},g\rangle _{L_2(\Omega )}\big |_{\vee _{\mathscr {T}_q} {\setminus } \Lambda ^{\mathscr {T}_q}(\mathcal {T},k)}\big \Vert&\lesssim 2^{-k/2} \Vert g\Vert _{L_2(\Omega )} \\ \big \Vert \langle \nabla \Psi ^{\mathscr {U}},\vec {g}\rangle _{L_2(\Omega )^n}\big |_{\vee _{\mathscr {U}} {\setminus } \Lambda ^{\mathscr {U}}(\mathcal {T},k)}\big \Vert&\lesssim 2^{-k/2} \Vert \vec {g}\Vert _{L_2(\Omega )^n} \end{aligned}$$

(uniform in ${\mathcal {T}}$, *g*, and $\vec {g}$).

### Proof

Since by assumption ($w_{2}$), for $\lambda \in \vee _{{\mathscr {T}_q}} {\setminus } \Lambda ^{{\mathscr {T}_q}}(\mathcal {T},k)$, $\mathrm{supp}\,\psi ^{{\mathscr {T}_q}}_\lambda$ has non-empty intersection with a uniformly bounded number of $\omega \in {\mathcal {T}}$, we have

A.3 $$\begin{aligned} \sum _{\lambda \in \vee _{{\mathscr {T}_q}} {\setminus } \Lambda ^{{\mathscr {T}_q}}(\mathcal {T},k)} \big |\sum _{\omega \in {\mathcal {T}}} \langle \psi ^{{\mathscr {T}_q}}_\lambda , g\rangle _{L_2(\omega )}\big |^2 \lesssim \sum _{\omega \in {\mathcal {T}}} \sum _{\lambda \in \vee _{{\mathscr {T}_q}} {\setminus } \Lambda ^{{\mathscr {T}_q}}(\mathcal {T},k)} |\langle \psi _\lambda ^{{\mathscr {T}_q}}, g\rangle _{L_2(\omega )}|^2. \end{aligned}$$

Given $\omega \in {\mathcal {T}}$ and $\ell \in \mathbb {N}_0$, we set

$$\begin{aligned} \Lambda _{\omega ,\ell }^{(1)}&=\{\lambda \in \vee _{{\mathscr {T}_q}}:|\lambda |=\ell ,\,\mathrm{supp}\,\psi _\lambda ^{{\mathscr {T}_q}} \subset \omega ,\,\psi _\lambda ^{{\mathscr {T}_q}} \text { has a vanishing moment}\}\\ \Lambda _{\omega ,\ell }^{(2)}&=\{\lambda \in \vee _{{\mathscr {T}_q}}{\setminus } \Lambda _{\omega ,\ell }^{(1)} :|\lambda |=\ell ,\,{{\mathrm{meas}}}(\mathrm{supp}\,\psi _\lambda ^{{\mathscr {T}_q}} \cap \omega )>0 \}. \end{aligned}$$

For $\lambda \in \Lambda _{\omega ,\ell }^{(2)}$, we estimate

A.4 $$\begin{aligned} |\langle \psi ^{{\mathscr {T}_q}}_\lambda , g\rangle _{L_2(\omega )}| \le \Vert \psi ^{{\mathscr {T}_q}}_\lambda \Vert _{L_1(\Omega )} \Vert g\Vert _{L_\infty (\omega )} \lesssim 2^{-\ell n /2} 2^{|\omega | n/2}\Vert g\Vert _{L_2(\omega )}. \end{aligned}$$

Using that $\# \Lambda _{\omega ,\ell }^{(2)} \lesssim 2^{(\ell -|\omega |)(n-1)}$ (cf. ($w_{4}$)), we infer that

A.5 $$\begin{aligned} \sum _{\ell >|\omega |+k} \sum _{\lambda \in \Lambda _{\omega ,\ell }^{(2)}} |\langle \psi _\lambda ^{{\mathscr {T}_q}}, g\rangle _{L_2(\omega )}|^2 \lesssim 2^{-k} \Vert g\Vert ^2_{L_2(\omega )}. \end{aligned}$$

Using that for $\lambda \in \Lambda _{\omega ,\ell }^{(1)}$, $\psi _\lambda ^{{\mathscr {T}_q}}$ has a vanishing moment, we find that

A.6 $$\begin{aligned} |\langle \psi ^{{\mathscr {T}_q}}_\lambda , g\rangle _{L_2(\omega )}| \lesssim \Vert \psi ^{{\mathscr {T}_q}}_\lambda \Vert _{L_1(\Omega )} 2^{-\ell } \Vert g\Vert _{W_\infty ^1(\omega )} \lesssim 2^{-\ell n/2} 2^{-\ell } 2^{|\omega |} 2^{|\omega |n/2} \Vert g\Vert _{L_2(\omega )}. \end{aligned}$$

From $\# \Lambda _{\omega ,\ell }^{(1)} \lesssim 2^{(\ell -|\omega |)n}$, we obtain

A.7 $$\begin{aligned} \sum _{\ell >|\omega |+k} \sum _{\lambda \in \Lambda _{\omega ,\ell }^{(1)}} |\langle \psi _\lambda ^{{\mathscr {T}_q}}, g\rangle _{L_2(\omega )}|^2 \lesssim 4^{-k} \Vert g\Vert ^2_{L_2(\omega )}. \end{aligned}$$

The proof of the first inequality follows from (A.3), (A.5), and (A.7).

To prove the second inequality, for any $\lambda \in \vee _{\mathscr {U}}$, similar to (A.4) we have

$$\begin{aligned} |\langle \nabla \psi ^{\mathscr {U}}_\lambda , \vec {g}\rangle _{L_2(\omega )^n}| \le \Vert \psi ^{\mathscr {U}}_\lambda \Vert _{W^1_1(\Omega )} \Vert \vec {g}\Vert _{L_\infty (\omega )^n} \lesssim 2^{-|\lambda | n /2} 2^{|\omega | n/2}\Vert \vec {g}\Vert _{L_2(\omega )^n}. \end{aligned}$$

When $\psi ^{\mathscr {U}}_\lambda$ has a vanishing moment and $\mathrm{supp}\,\psi ^{\mathscr {U}}_\lambda \subset \omega$, we have

$$\begin{aligned} |\langle \nabla \psi ^{\mathscr {U}}_\lambda , \vec {g}\rangle _{L_2(\omega )^n}|&= |\langle \psi ^{\mathscr {U}}_\lambda , {{\mathrm{div}}}\vec {g}\rangle _{L_2(\omega )}| \lesssim \Vert \psi ^{\mathscr {U}}_\lambda \Vert _{L_1(\Omega )} 2^{-|\lambda |} \Vert \vec {g}\Vert _{W_\infty ^2(\omega )^n}\\&\lesssim 2^{-|\lambda | n/2} 4^{-|\lambda |} 4^{|\omega |} 2^{|\omega |/2} \Vert \vec {g}\Vert _{L_2(\omega )^n} \end{aligned}$$

replacing (A.6). From these two estimates, the second inequality follows similarly to the first one. $\square$

The following proposition has been used to bound the term $\Vert \mathbf{r}^{(\frac{1}{2})}_3-{\tilde{\mathbf{r}}}^{(\frac{1}{2})}_3\Vert$ in the proof of Theorem 5.14.

### Proposition A.5

For a boundary tiling ${\mathcal {T}}_{\Gamma _D} \subset \mathcal {O}_{\Gamma _D}$, and $g \in \mathcal {P}_m({\mathcal {T}}_{\Gamma _D}) \cap C(\Gamma _D)$,

$$\begin{aligned} \Vert \langle \Psi ^{\mathscr {V}_2},g\rangle _{L_2(\Gamma _D)}|_{\vee _{\mathscr {V}_2}{\setminus } \Lambda ^{\mathscr {V}_2}({\mathcal {T}}_{\Gamma _D},k)}\Vert \lesssim 2^{-k/2}\Vert g\Vert _{H^{\frac{1}{2}}(\Gamma _D)}. \end{aligned}$$

### Proof

Using (5.8), similar to (A.1)

$$\begin{aligned} \begin{aligned} \sum _{\lambda \in \vee _{\mathscr {V}_2}{\setminus } \Lambda ^{\mathscr {V}_2}({\mathcal {T}}_{\Gamma _D},k)} \Big | \int _{\Gamma _D} g \psi _\lambda ^{\mathscr {V}_2} \, ds\Big |^2&\le \sum _{\lambda \in \vee _{\mathscr {V}_2}{\setminus } \Lambda ^{\mathscr {V}_2}({\mathcal {T}}_{\Gamma _D},k)} \sum _{\omega \in {\mathcal {T}}_{\Gamma _D}} 2^{-|\lambda |} \Vert g\Vert ^2_{H^1(\omega \cap \mathrm{supp}\,\psi ^{\mathscr {V}_2}_\lambda )}\\&\lesssim 2^{-k} \sum _{\omega \in {\mathcal {T}}_{\Gamma _D}} 2^{-|\omega |} \Vert g\Vert _{H^1(\omega )}^2 \lesssim 2^{-k} \Vert g\Vert ^2_{H^{\frac{1}{2}}(\Gamma _D)}, \end{aligned} \end{aligned}$$

by an application of an inverse inequality. $\square$

The following proposition has been used to bound the term $\Vert \mathbf{r}_3-{\tilde{\mathbf{r}}}_3\Vert$ in the proof of Theorem 5.14.

### Proposition A.6

For a boundary tiling ${\mathcal {T}}_{\Gamma _D} \subset \mathcal {O}_{\Gamma _D}$, and $g \in \mathcal {P}_m({\mathcal {T}}_{\Gamma _D})$,

$$\begin{aligned} \Vert \langle \Psi ^{\mathscr {U}},g\rangle _{L_2(\Gamma _D)}|_{\vee _{\mathscr {U}}{\setminus } \Lambda ^{\mathscr {U}}({\mathcal {T}}_{\Gamma _D},k)}\Vert \lesssim 2^{-k/2}\Vert g\Vert _{H^{-\frac{1}{2}}(\Gamma _D)}. \end{aligned}$$

### Proof

In the proof of Proposition A.1, we saw that $\Vert \Psi _\lambda ^{\mathscr {V}_1}\Vert _{L_2(\Gamma _N)} \lesssim 2^{-|\lambda |/2}$. The same arguments show that $\Vert \Psi _\lambda ^\mathscr {U}\Vert _{L_2(\Gamma _D)} \lesssim 2^{-|\lambda |/2}$. Consequently, similar to (A.1),

$$\begin{aligned} \begin{aligned} \sum _{\lambda \in \vee _{\mathscr {U}}{\setminus } \Lambda ^{\mathscr {U}}({\mathcal {T}}_{\Gamma _D},k)} \Big | \int _{\Gamma _D} g \psi _\lambda ^{\mathscr {U}} \, ds\Big |^2&\le \sum _{\lambda \in \vee _{\mathscr {U}}{\setminus } \Lambda ^{\mathscr {U}}({\mathcal {T}}_{\Gamma _D},k)} \sum _{\omega \in {\mathcal {T}}_{\Gamma _D}} 2^{-|\lambda |} \Vert g\Vert ^2_{L_2(\omega \cap \mathrm{supp}\,\psi ^{\mathscr {U}}_\lambda )}\\&\lesssim 2^{-k} \sum _{\omega \in {\mathcal {T}}_{\Gamma _D}} 2^{-|\omega |} \Vert g\Vert _{L_2(\omega )}^2 \lesssim 2^{-k}\Vert g\Vert ^2_{H^{-\frac{1}{2}}(\Gamma _D)}, \end{aligned} \end{aligned}$$

where the last inequality follows from analogous arguments as were applied in the first paragraph of the proof of Lemma A.2. $\square$

## Footnotes

1 Indeed, for an admissible ${\bar{\mathbf{u}}}$ with $\Vert \mathbf{u}-{\bar{\mathbf{u}}}\Vert \le \varepsilon$ and $\#\,\mathrm{supp}\,{\bar{\mathbf{u}}} \lesssim \varepsilon ^{-1/s}$, take $f_\varepsilon =-\bar{u}''$ and use $\Vert f+\bar{u}''\Vert _{H^{-1}(\Omega )} \eqsim \Vert u-\bar{u}\Vert _{H^1(0,1)}\eqsim \Vert \mathbf{u}-{\bar{\mathbf{u}}}\Vert$.

2 For general non-affine $\vec {H}$, $\vec {H}^\mathrm{h}$ should be read as the Fréchet derivative $D\vec {H}(u,\theta )$.

3 Actually, in the current setting its analysis is more straightforward, because the residuals are measured in $L_2(0,1)$ instead of in $H^{-1}(0,1)$.
